# SEACells infers transcriptional and epigenomic cellular states from single-cell genomics data

**DOI:** 10.1038/s41587-023-01716-9

**Published:** 2023-03-27

**Authors:** Sitara Persad, Zi-Ning Choo, Christine Dien, Noor Sohail, Ignas Masilionis, Ronan Chaligné, Tal Nawy, Chrysothemis C. Brown, Roshan Sharma, Itsik Pe’er, Manu Setty, Dana Pe’er

**Affiliations:** 1 Computational and Systems Biology Program, Sloan Kettering Institute, Memorial Sloan Kettering Cancer Center, New York, NY USA; 2 Department of Computer Science, Fu Foundation School of Engineering & Applied Science, Columbia University, New York, NY USA; 3 Basic Sciences Division, Fred Hutchinson Cancer Center, Seattle, WA USA; 4 Computational Biology Program, Public Health Sciences Division and Translational Data Science IRC, Fred Hutchinson Cancer Center, Seattle, WA USA; 5 Human Oncology and Pathogenesis Program, Memorial Sloan Kettering Cancer Center, New York, NY USA; 6 Department of Pediatrics, Memorial Sloan Kettering Cancer Center, New York, NY USA; 7 Howard Hughes Medical Institute, New York, NY USA

**Keywords:** Computational models, Gene regulatory networks

## Abstract

Metacells are cell groupings derived from single-cell sequencing data that represent highly granular, distinct cell states. Here we present single-cell aggregation of cell states (SEACells), an algorithm for identifying metacells that overcome the sparsity of single-cell data while retaining heterogeneity obscured by traditional cell clustering. SEACells outperforms existing algorithms in identifying comprehensive, compact and well-separated metacells in both RNA and assay for transposase-accessible chromatin (ATAC) modalities across datasets with discrete cell types and continuous trajectories. We demonstrate the use of SEACells to improve gene–peak associations, compute ATAC gene scores and infer the activities of critical regulators during differentiation. Metacell-level analysis scales to large datasets and is particularly well suited for patient cohorts, where per-patient aggregation provides more robust units for data integration. We use our metacells to reveal expression dynamics and gradual reconfiguration of the chromatin landscape during hematopoietic differentiation and to uniquely identify CD4 T cell differentiation and activation states associated with disease onset and severity in a Coronavirus Disease 2019 (COVID-19) patient cohort.

## Abstract (web summary)

Single-cell aggregation with archetypes provides intermediate granularity for downstream analyses.

## Main

A fundamental disconnect currently exists between the cellular resolution of single-cell genomics data and the cluster-level resolution of most analyses. To overcome the sparsity and noise of these data, tens of thousands of cells are typically summarized by a small set of clusters. Clustering also makes it feasible to analyze large single-cell RNA sequencing (scRNA-seq) datasets. As projects such as the Human Cell Atlas^1^ and the Human Tumor Atlas Network^2^ scale to millions of cells, even routine dimensionality reduction and visualization tasks struggle with computational complexity. Sparsity and noise are especially problematic in single-cell assay for transposase-accessible chromatin sequencing (scATAC-seq) data, which capture only trinary zygosity states at a few thousand of the hundreds of thousands of open chromatin regions in any individual cell (Supplementary Fig. 1), rendering aggregation essential.

**Fig. 1:**
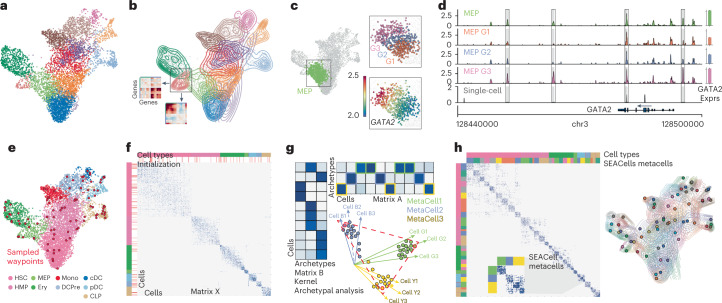
Overview of the SEACells algorithm for cell state identification from single-cell data. **a**, scRNA-seq UMAP of 6,800 CD34^+^ sorted hematopoietic stem and progenitor cells (HSPCs). Cells are colored by cluster. **b**, Contour plots of each cluster highlight density and indicate the presence of multiple cell states within each cluster. Inset: gene–gene covariance matrices reveal that each state includes multiple distinct gene expression programs. **c**, Left: UMAP indicating the MEP (megakaryocyte-erythroid progenitor) cluster. Right: when the MEP cluster is divided into three equal-sized bins based on developmental progression (top, G1 to G3), it reflects imputed expression of *GATA2* (known driver of MEP lineage) (bottom). **d**, Coverage plots showing *GATA2* accessibility in all MEPs (top), in a single MEP cell (bottom) and in the three bins in **c**. Right: expression of *GATA2* in corresponding cells. Highlighted peaks demonstrate how accessibility dynamics track with expression dynamics. Information about dynamics is masked at the cluster level, whereas peak identification in single cells is too noisy. **e**, UMAP as in **a**, colored by cell type. The SEACells algorithm for metacell identification is initialized by waypoints (large red circles), a subset of cells sampled to uniformly cover the phenotypic landscape. **f**, Cell-to-cell affinity matrix computed using an adaptive Gaussian kernel. Cells are sorted by cell types (top annotation row). Second annotation row shows the SEACells initialization. **g**, Schematic of kernel archetypal analysis. The kernel matrix is decomposed into the archetype matrix B and embedding matrix A. Metacell membership is identified based on column-wise maximal values across the matrix A. Inset: Kernel archetypal analysis partitions cells into clusters of highly similar cells, making it ideally suited to the identification of robust cell states. **h**, Left: cell–cell affinity matrix from **f** but ordered by metacell assignment. Right: contour plot overlying UMAP from **e**, highlighting the distribution of metacells; cells and contours are colored by metacell assignment.

A typical cluster, however, is not homogenous (Fig. 1a,b). Moreover, single-cell data have been shown to reside on a continuum^3–6^. For instance, binning the expression of *GATA2*, a driver of erythroid fate, in one cluster of erythroid precursor cells^7^ demonstrates gradual cell state changes within each bin (Fig. 1c). The accessibility landscape of the *GATA2* locus suggests that its expression dynamics are enabled by gradual opening of regulatory elements (Fig. 1d). Such dynamics are lost in discrete cluster-level analysis.

The concept of metacells^8^—groups of cells that represent distinct cell states, whereby within-metacell variation is due to technical rather than biological sources—was proposed as a way of maintaining statistical utility while maximizing effective data resolution^8^. Metacells are far more granular than clusters and are optimized for homogeneity within cell groups rather than for separation between clusters. However, existing approaches^8–10^ fail on scATAC-seq data; aggressively cull outliers (particularly inappropriate for disease studies, which are often driven by rare cell populations); and are poorly distributed across the phenotypic space. Consequently, metacells are not routinely used in single-cell analysis, and scATAC-seq data have remained underused.

Here we present single-cell aggregation of cell states (SEACells), a graph-based algorithm that uses kernel archetypal analysis to compute metacells. We demonstrate that SEACells metacells provide robust, comprehensive characterizations of scRNA-seq cell states and that they successfully describe chromatin cell states at resolutions that enable the inference of regulatory elements underlying gene expression. Our metacells achieve a sweet spot between signal aggregation and cellular resolution, and they capture cell states across the phenotypic spectrum, including rare states. We further show that our metacells retain subtle biological differences between samples that are removed as batch effects by alternative methods and, thus, provide a better starting point than sparse individual cells for data integration. SEACells provides a toolkit for gene regulatory inference from scATAC-seq data and an effective statistic for integrating single-cell data from large cohorts.

## Results

### SEACells identifies metacells across the phenotypic manifold

SEACells seeks to aggregate single cells into metacells that represent distinct cellular states, in a manner agnostic to data modality. Using a count matrix as input, it provides per-cell weights for each metacell, per-cell hard assignments to each metacell and the aggregated counts for each metacell as output. Notably, our approach captures the full spectrum of cell states in the data, including rarer states. We base SEACells on a few key assumptions: (1) single-cell profiling data can be approximated by a lower-dimensional manifold (phenotypic manifold); (2) much of the observed variability across cells is due to incomplete sampling; and (3) most cells can be assigned to a finite set of cell states, each characterized by a distinct combination of active gene regulatory programs.

SEACells takes advantage of graph-based algorithms for manifold learning that have been proven to capture the cell state landscape in single-cell genomics data faithfully and robustly^3,5,6,11–13^. The algorithm first constructs a nearest neighbor graph representing the phenotypic manifold. It then applies archetypal analysis^14,15^ to iteratively refine metacells. Finally, it aggregates counts into a set of output metacells. Manifold construction is tailored to each data modality, after which the algorithm can proceed in a data-type-agnostic fashion (Supplementary Fig. 2). We use CD34^+^ cells from early human hematopoiesis to demonstrate our method (Fig. 1). We use minimum–maximum sampling^4^ for initialization, which identifies a set of representative cell states that are distributed uniformly across the phenotypic manifold (Fig. 1e) and is particularly adept at dealing with density differences, thus ensuring the capture of rare states. These sampled cells are waypoints (multiple per cell type) that define clear structure in the neighbor graph; however, the cell states themselves remain somewhat diffuse (Fig. 1f).

**Extended Data Fig. 1:**
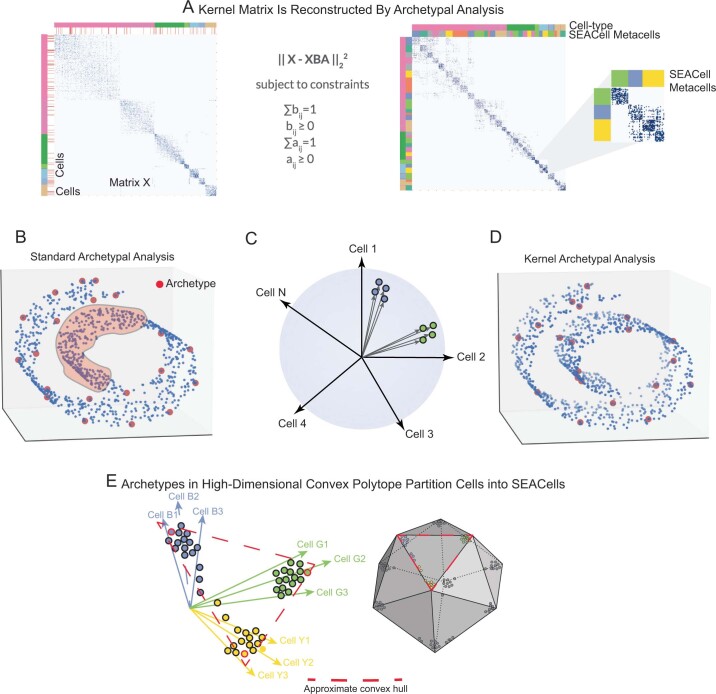
Kernel archetypal analysis to identify metacells. **A**. The kernel matrix (left) is decomposed into two the archetype matrix B and embedding matrix A. Metacell membership is identified based on column-wise maximal values across the matrix A. Right: The kernel matrix but ordered by metacell assignment. **B**. Standard archetypal analysis which uses linear convex hull approximation leads to identification of archetypes at the boundaries with no archetypes in internal regions of the manifold (highlighted region). **C**. The use of cell-cell similarity kernels to describe single-cell data casts highly similar cells into tiny clusters along a cone emanating from the origin. **D**. Kernel archetypal analysis opens up the interior regions and thus archetypes are identified across the manifold. **E**. The greater number of archetypes that result from kernel archetypal analysis, in addition to the use of a Gaussian kernel, creates a large number of highly similar cell ‘pockets’, each representing a unique biological state.

To refine metacells, we employ kernel archetypal analysis (Fig. 1g, Extended Data Fig. 1a and Methods). Archetypal analysis^16^ is a robust matrix decomposition technique that has been applied to the data matrix to identify extreme cell states at the boundaries of cellular phenotypic space^14^. Instead, we apply archetypal analysis to the cell–cell similarity kernel matrix. This kernel projects cells into a higher-dimensional space wherein two cells are alike only if they share neighbors and the distances to the shared neighbors are similar. The stricter similarity conditions imposed by this transformation projects highly similar cells into tiny clusters, such that boundary cells are the most similar to every other cell in their cluster, making archetypes in kernel space good representatives of each unique cellular state. Kernel archetypal analysis, thus, partitions cells into tight clusters of highly similar cells (Fig. 1g and Extended Data Fig. 1), conferring tight blocks along the diagonal of the cell–cell similarity matrix that represent distinct cell states (Fig. 1h).

### SEACells metacells represent accurate and robust cell states

**Fig. 2:**
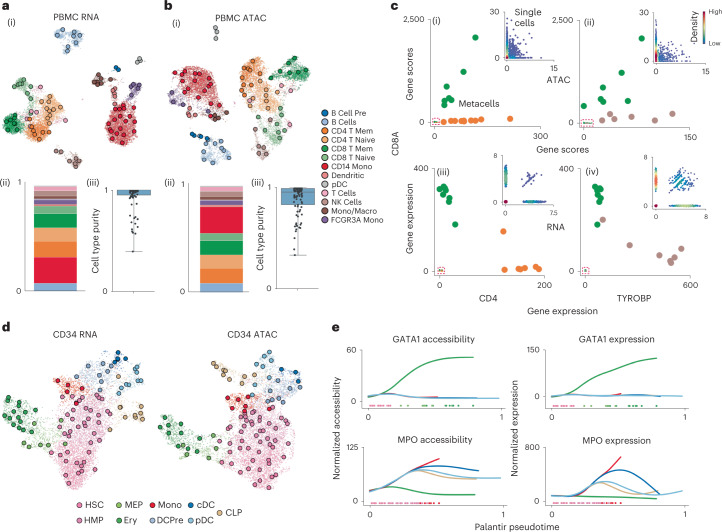
SEACells metacells accurately identify cell states and outperform alternative approaches. **a**, (i) UMAP of human PBMCs in a 10x Genomics multiome dataset^17^, derived using RNA data, highlighting cell types and SEACells metacells. (ii) Distribution of metacells per cell type for the RNA modality. (iii) Distribution of cell type purity (frequency of the most represented cell type in each metacell). High purity represents a more accurate metacell. Boxes and line represent interquartile range (IQR) and median, respectively; whiskers represent ±1.5× IQR. **b**, UMAP, metacell and cell type purity distributions of human PBMCs as in **a**, using ATAC data from the multiome dataset. **c**, Metacell accessibility (i) and expression (iii) of *CD4* and *CD8A* accurately distinguish CD8 (green) and CD4 (orange) T cell compartments. Metacell accessibility (ii) and expression (iv) of *TYROBP* and *CD8A* distinguish NK (brown) and CD8 (green) T cells. Insets: Corresponding single-cell accessibility is too sparse to achieve the same distinction. **d**, UMAPs of CD34^+^ HSPCs highlighting cell types and the SEACells metacells independently constructed from RNA (left) and ATAC (right) data. **e**, Accessibility (left) and expression (right) of *GATA1* (erythroid factor) and *MPO* (myeloid factor) along the Palantir pseudotime axis representing hematopoietic differentiation. Palantir was run on RNA aggregates using ATAC metacells and accurately recapitulates dynamics.

We first evaluated the performance of SEACells on a public multiome (simultaneous scRNA-seq and ATAC-seq) dataset of peripheral blood mononuclear cells (PBMCs)^17^ from 10x Genomics, as a well-studied system with distinct cell populations. We found that SEACells metacells are comprehensive, well distributed among cell types and exhibit a high degree of cell type purity in both RNA and ATAC data (Fig. 2a,b and Methods). Furthermore, reciprocal projections of RNA and ATAC metacells demonstrate that metacells of different modalities are highly concordant (Supplementary Fig. 3a,b and Methods).

Metacells help overcome sparsity, which is extreme in scATAC-seq data. We found that each SEACells metacell provides a more complete molecular characterization than individual cells—for example, by revealing accessibility at known marker genes for major cell types. Accessibility and expression from metacells, but not most individual cells, can accurately distinguish between lymphoid subsets (Fig. 2c and Supplementary Fig. 3c,d). Metacells, thus, comprise pure cell types; they are granular enough to distinguish states within cell types; and they can be queried with classical immune markers.

To test SEACells in a trajectory setting, we collected a multiome dataset of 6,800 hematopoietic stem and progenitor cells (HSPCs) from healthy bone marrow sorted for pan-HSPC marker CD34 (Methods). Similar to PBMCs, metacells are well distributed across all cell types and span the RNA and ATAC phenotypic manifolds (Fig. 2d). To determine whether metacell resolution is sufficient to recover gene expression dynamics that are lost in clustering, we applied the Palantir trajectory algorithm^4^ directly to metacells. Palantir recovered the known expression and accessibility dynamics of key hematopoietic genes (Supplementary Fig. 4). As a further challenge, we ran Palantir on aggregated RNA from metacells computed on the ATAC modality (Fig. 2e and Supplementary Fig. 4). The fidelity of captured gene trends reinforces that SEACells metacells overcome sparsity while retaining dynamics in systems with continuous state transitions.

**Extended Data Fig. 2:**
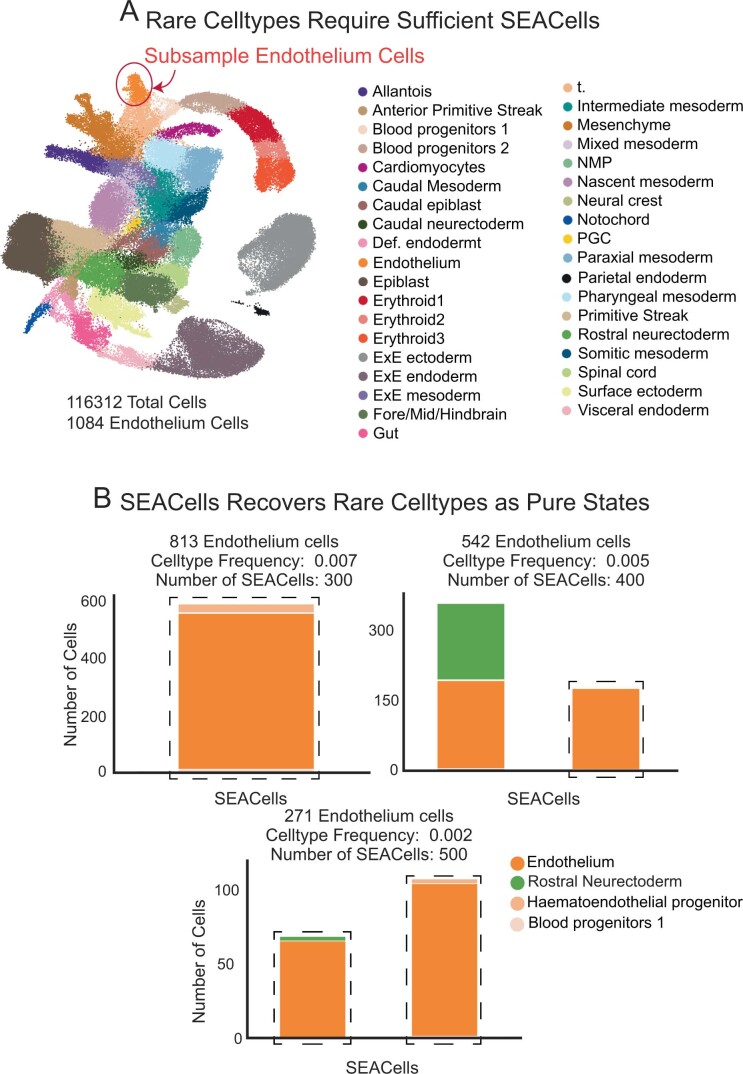
Sensitivity of SEACells algorithm to detect rare cell-types. **A**. UMAP of the mouse gastrulation atlas data^1^, with endothelial cells highlighted. **B**. Bar plots showing the cell-type composition of metacells containing at least 50% endothelial cells with various subsampling of endothelial cells from the mouse gastrulation data.

We used the CD34^+^ bone marrow and PBMC datasets to assess the robustness of SEACells (Methods). SEACells results in high-confidence partitioning of cells into distinct metacells (Supplementary Fig. 5), which are consistent across different initializations (Supplementary Fig. 6a) and numbers of SEACells (Supplementary Fig. 6b), for both RNA and ATAC modalities, based on normalized mutual information (NMI) score^18^. Another key performance metric is the ability to capture rare cell states. SEACells was able to accurately recover rare cell types, such as plasmacytoid dendritic cells (pDCs) and B cell precursors, in the PBMC RNA and ATAC modalities (Fig. 2a,b). To further test the ability of SEACells to identify rare intermediate cell states in continuous trajectories, we generated a second multiome dataset representing the full span of human hematopoiesis and found that SEACells can identify metacells in the diverse low-density regions that represent rare intermediate cells (Methods and Supplementary Fig. 7). As an additional assessment of the ability of SEACells to identify rare cell states, we systematically downsampled the mouse gastrulation atlas^19^ (Extended Data Fig. 2a) and recovered metacells that are exclusively composed of cell types comprising less than 0.2% of the total population (Extended Data Fig. 2b), demonstrating the sensitivity of SEACells.

### SEACells empowers gene regulatory inference

Gene regulation can be inferred by identifying putative transcription factor (TF) binding motifs within ATAC-seq read count peaks, which represent open or accessible chromatin regions. scATAC-seq provides many observations (cells) with the potential to infer more complex gene regulatory models at fine resolution^20–22^, but data sparsity has severely restricted its utility by restricting most analyses to cluster resolution. We surmised that SEACells metacells provide an ideal tradeoff between fine resolution and sufficient coverage to overcome sparsity for diverse gene regulatory inference tasks.

A typical SEACells metacell contains 1.2 million reads, a substantial improvement over the 25,000 reads in an individual cell, but still far fewer than the 50 million reads in a typical bulk sample. To improve the signal-to-noise ratio in ATAC peak calling, we took advantage of the characteristic ATAC-seq fragment length distribution (Supplementary Fig. 8a)^23^, in which the first and second modes represent nucleosome-free (NFR) fragments (likely enriched for TF binding events) and nucleosomes, respectively. Peaks called using all fragments tend to resolve regulatory elements poorly (Supplementary Fig. 8b). In contrast, we found that using NFR fragments alone identifies fewer peaks overall, but these are enriched for potentially TF-bound open chromatin and include many peaks that are obscured when considering all fragments (Supplementary Fig. 8b,c). Regulatory element identification, thus, benefits from using NFR fragments rather than all fragments.

**Fig. 3:**
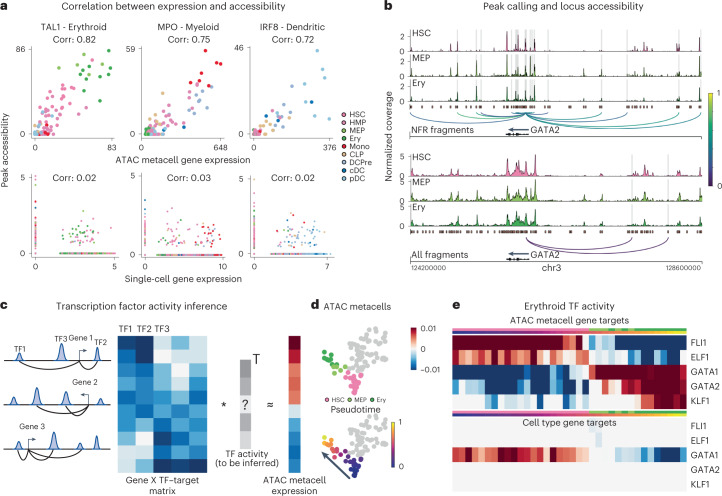
SEACells empowers a gene regulatory toolkit. **a**, Spearman correlation between ATAC metacell-aggregated (top) or single-cell (bottom) gene expression and accessibility of the most correlated peak in *TAL1* (erythroid), *MPO* (myeloid) and *IRF* (dendritic) marker genes, computed on CD34 multiome data. Each metacell and single cell is colored based on cell type. **b**, Accessibility landscape of erythroid factor *GATA2* in HSCs, MEPs and erythroid cells (Ery) using NFR (top) or all ATAC (bottom) fragments. Restricting chromatin accessibility analysis to NFR fragments improves peak resolution and the association of regulatory elements with genes. Arcs are colored by peak–gene Spearman correlation (color values between 0 and 1 at right), determined using SEACells ATAC metacells. Highlighted peaks correlate significantly with *GATA2* expression (two-sided nominal *P* < 0.1, empirical null distribution). **c**, Left: To construct a TF–target matrix for TF activity inference, motif scores of motifs within peaks are weighted by peak–gene correlations, as identified using SEACells metacells, for each gene. Right: The SEACells-derived TF–target matrix is used to predict the expression profile of a metacell and to infer TF activities per metacell. **d**, UMAPs highlighting metacells of the erythroid lineage, colored by pseudotime. **e**, Top: activities of top TFs across metacells of the erythroid lineage using the SEACells-derived TF–target matrix as input. Bottom: activities of the same TFs derived using the cell-type-specific TF–target matrix derived from pseudobulk ATAC profiles.

The next task in regulatory inference is to associate each gene with the elements that regulate it. The correlation between accessibility and expression across cells has been used to predict the peak set that regulates each gene using either multiome^20^ or integrated scRNA-seq and scATAC-seq data^24^, but data sparsity precludes robust correlation at the single-cell level. Using SEACells metacells from the CD34^+^ bone marrow ATAC data, we computed correlations between gene expression and accessibility of each NFR peak within ±100 kb of each gene in a core hematopoietic gene set^5^. Accessibility of the most correlated peak using ATAC metacells faithfully tracks with gene expression, representing a substantial improvement over single-cell correlation (Fig. 3a and Supplementary Fig. 9). For example, the correlation between peak accessibility and expression in metacells for key erythroid lineage regulator *TAL1* is 0.82, and cells on the erythroid trajectory exhibit the highest values, whereas the correlation is 0.03 at the single-cell level, with no distinction among erythroid cells (Fig. 3a).

**Extended Data Fig. 3:**
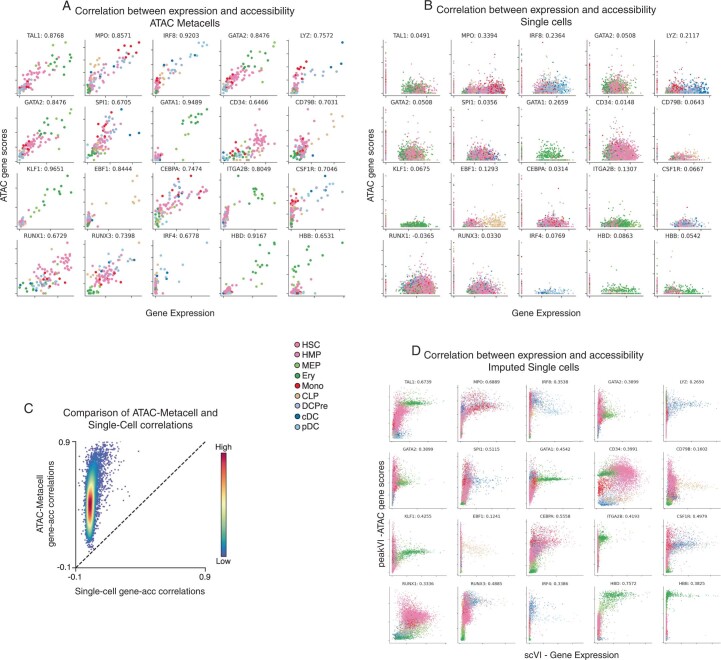
Comparison of ATAC gene scores using SEACells metacells and single cells. **A**. Relationship between metacell-aggregated gene expression and ATAC gene scores for a selection of key hematopoietic genes, computed on the CD34^+^ multiome data (Metacells computed on ATAC modality). Gene scores for metacells were computed by aggregating peaks that correlate significantly with expression. Spearman correlations appear next to the gene symbol. **B**. Same as in (A), but at single-cell level. Gene scores for single-cell data were computed using ArchR. **C**. Spearman correlations between gene expression and ATAC gene scores, plotted for metacells against single cells. Genes are colored by density. **D**. Correlation between scVI^2^-imputed gene expression and gene scores derived following peakVI^3^ imputation of peak accessibility for the genes in (A).

To build a comprehensive map of regulatory elements, we identified all peaks significantly correlated with a gene compared to GC-content-matched peaks sampled from the data^20^ (Methods). For the key erythroid factor *GATA2*, single-cell data recover only two of 11 associations detected using metacells (Fig. 3b). To systematically explore the accuracy of predicted peak–gene associations, we computed gene scores^24^ by aggregating the accessibility of all significantly correlated peaks and comparing them to gene expression (Methods). SEACells gene scores are substantially better correlated than scores derived using the aggregate of all correlated peaks for both unimputed (Extended Data Fig. 3a–c) and imputed (Extended Data Fig. 3d) single-cell data^25^. SEACells metacells, thus, clearly identify *cis*-regulatory elements that are significantly correlated with expression and likely regulate the corresponding gene.

**Extended Data Fig. 4:**
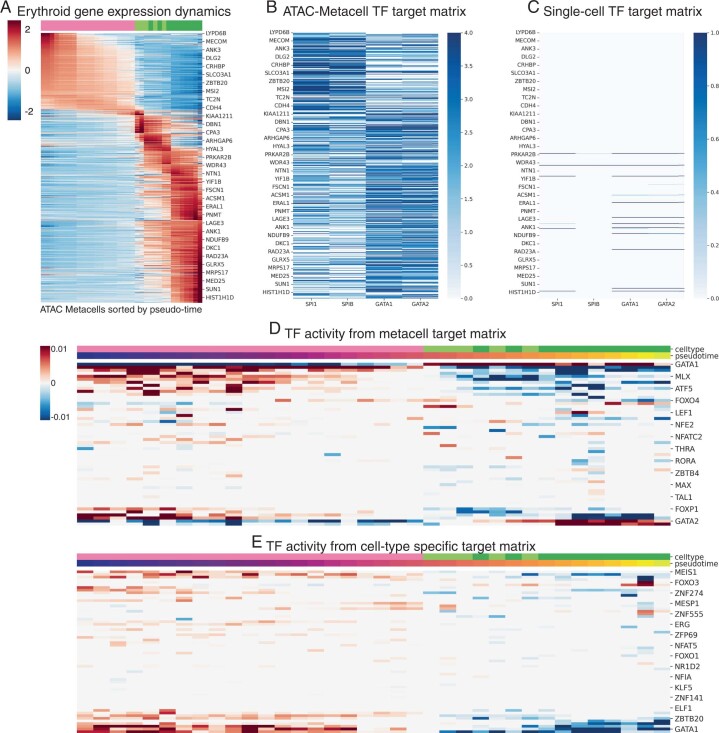
Inference of TF activities along erythroid lineage. **A**. z-scored expression of erythroid lineage metacells. The gene set was determined using differential expression between cell-types at the single-cell level. Each column represents a metacell along the erythroid lineage. **B**. TF-target matrix for SPI, SPIB, GATA1 and GATA2 for the same set of genes in (A). **C**. Same as (B), using the peak-gene associations derived using single cells. **D**. TF activities derived by applying the lasso regression approach (Methods) to the metacell derived TF-target matrix. **E**. TF activities derived by applying the lasso regression approach (Methods) to a TF-target matrix derived using cell-type-specific peaks.

As a proof of principle for regulatory network inference using SEACells, we devised a simple procedure to infer TF activities. We first determined the enriched motifs present in each peak and summarized the motif scores in peaks associated with each gene to construct a TF–gene target matrix (Fig. 3c and Methods). We then predicted expression in each metacell as a function of this matrix using lasso regression^26^ and employed a feature ranking procedure^27^ to determine the TF subset that best explains the expression profile of each metacell. We applied this procedure to our CD34^+^ multiome data to identify the key TFs along the erythroid lineage (Fig. 3d,e and Extended Data Fig. 4a). TF–target matrices constructed using single-cell associations are extremely sparse and unreliable compared to matrices constructed using ATAC metacells (Extended Data Fig. 4b,c). Using metacell TF–target matrices to predict expression in each metacell and infer the regulatory activity of each TF (Extended Data Fig. 4d), we successfully recovered activation by known erythroid regulators, such as *GATA1* (ref. ^28^), *GATA2* (ref. ^4^) and *KLF1* (ref. ^7^), and downregulation of stem cell regulators, such as *FLI1* (ref. ^29^) and *ELF1* (ref. ^29^) (Fig. 3e). In contrast, the common approach of creating TF–target matrices using pseudobulk profiles of cell type clusters failed to accurately recover well-known erythroid regulators (Fig. 3e and Extended Data Fig. 4e). Furthermore, our approach generalizes to other major hematopoietic lineages (Supplementary Fig. 10) and successfully identifies top regulators, demonstrating that the peak–gene associations identified using SEACells provide a robust input for regulatory network inference.

**Extended Data Fig. 5:**
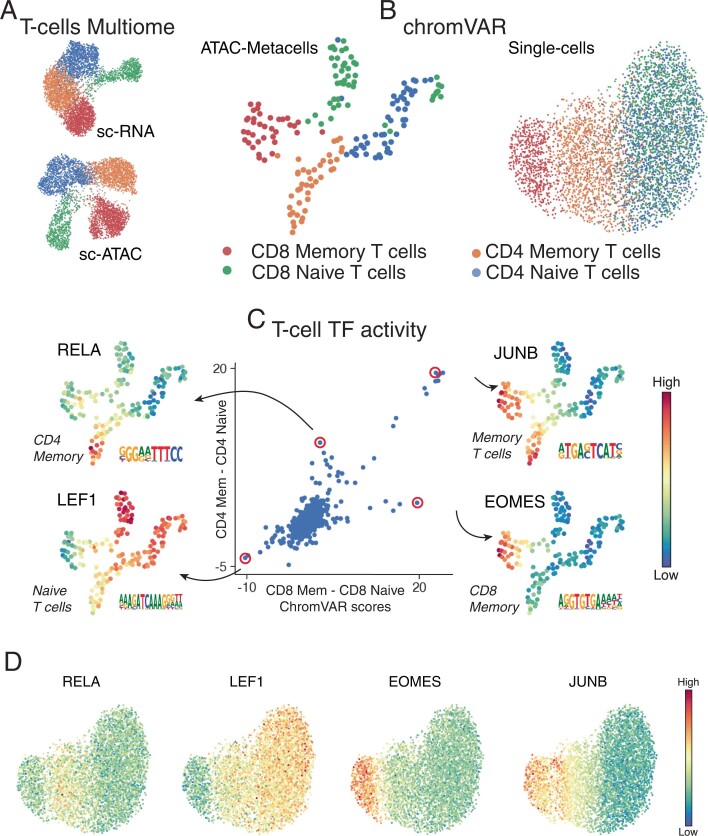
Single-cell chromVAR scores for T-cell subsets. **A**. RNA and ATAC UMAPs of the T-cell subset from the PBMC multiome dataset. **B**. UMAPs derived from chromVAR scores computed using single cells or metacell aggregates. All peaks were used for chromVAR analysis. Metacell chromVAR scores accurately recapitulate differences between T-cell subsets, whereas single-cell chromVAR scores fail to distinguish CD4^+^ and CD8^+^ T-cells. **C**. chromVAR score distributions can be used to identify key TFs that define different T-cell compartments. Each dot represents a TF. X-axis shows the difference between SEACells metacell chromVAR scores between the two CD8^+^ compartments. Y-axis shows the difference between SEACells metacell chromVAR scores between the two CD4^+^ compartments. **D.** UMAPs of T-cell subsets from PBMC multiome data (as in B) colored by single-cell chromVAR scores of key T-cell factors.

Another common strategy for overcoming sparsity is to compute a TF activity score by aggregating all peaks associated with a particular TF (for example, chromVAR^30^). To demonstrate that metacells can improve TF activity inference, we determined chromVAR scores for all T cell subsets (CD4, CD8 naive and memory) using the PBMC multiome dataset (Extended Data Fig. 5a). chromVAR scores provide an alternate data representation, useful for all downstream analyses, including clustering and visualization. Indeed, chromVAR scores using metacells accurately distinguish all T cell subsets, whereas single-cell chromVAR scores fail to distinguish CD8 and CD4 (Extended Data Fig. 5b). We identified several known compartment-specific TFs that likely drive cell states within these T cell subsets, including *JUNB*^31^, *LEF1* (ref. ^32^), *EOMES*^33^ and *RELA*^34^, whereas single-cell chromVAR scores for these factors do not distinguish the same populations (Extended Data Fig. 5c,d). Our results show that SEACells substantially improves the regulatory toolkit for analyzing and interpreting scATAC-seq data, including widely used tools such as chromVAR.

### SEACells outperforms metacell approaches for RNA and ATAC

Baran et al.^8^ introduced and effectively articulated the metacell concept. Their MetaCell algorithm was demonstrated on healthy systems and designed around massively parallel single-cell RNA sequencing (MARS-seq) data, which has a high instance of extreme values^35^, so it culls outliers aggressively. However, rare cells often drive disease and regeneration. We found that MetaCell^8^ discards more than one-third of all cells in lung adenocarcinoma scRNA-seq data^36^ (Supplementary Fig. 11a,b), and MetaCell-2 (ref. ^10^) behaves similarly. Another approach, SuperCell^9^, is effectively a very fine clustering strategy that adapts widely used community detection algorithms to generate many small clusters.

**Fig. 4:**
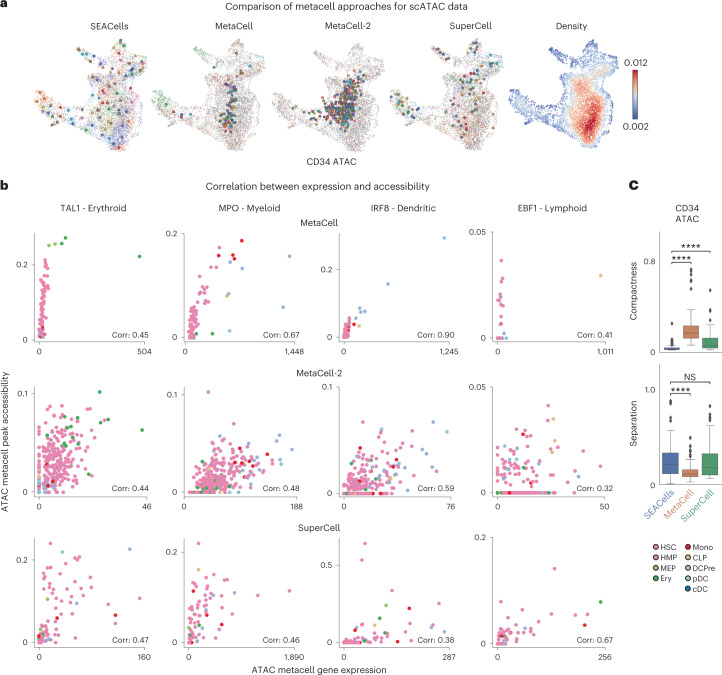
SEACells outperforms existing methods in cell state representation and correlation of expression and accessibility. **a**, ATAC modality UMAPs of CD34^+^ bone marrow (as in Fig. 2d), colored by metacell aggregates identified by the specified method or colored by cell density. Dots, cells; circles, metacells. **b**, Pearson correlation between metacell-aggregated gene expression and accessibility of the most correlated peak in *TAL1* (erythroid gene), *MPO* (myeloid gene), *IRF* (dendritic gene) and *EBF1* (lymphoid gene) using the CD34^+^ bone marrow ATAC metacells called by MetaCell (top), MetaCell-2 (middle) or SuperCell (bottom). **c**, Metacell metrics measured in the ATAC modality of CD34^+^ bone marrow multiome data. Top: metacell compactness (average diffusion component standard deviation; Methods). A lower score indicates more compact metacells. Bottom: metacell separation (distance between nearest metacell neighbor in diffusion space; Methods). Greater separation indicates better performance. Comparisons were carried out on all metacells (*n* = 86). Two-sided Wilcoxon rank-sum test; NS: *P* > 0.05, ****P < 0.0001. Boxes and line represent interquartile range (IQR) and median, respectively; whiskers represent ±1.5× IQR. NS, not significant.

We benchmarked these algorithms using ATAC and RNA modalities from the CD34^+^ bone marrow and PBMC datasets. Because both MetaCell and SuperCell require a gene count matrix, we aggregated peaks in the gene body to derive a count matrix for ATAC-seq data. SEACells was the only algorithm to identify metacells that cover the entire phenotypic landscape (Fig. 4a and Supplementary Fig. 12), likely due to its minimum–maximum sampling strategy. For ATAC, all other approaches neglected the majority of cell states by focusing metacells on cell-dense regions; they failed to represent important lymphoid and myeloid subpopulations in bone marrow and to identify coherent cell states in PBMCs (Fig. 4a). SuperCell severely undersampled metacells in low-density regions (Supplementary Fig. 12a) and did not accurately recover the distinction between different T cell states.

**Extended Data Fig. 6:**
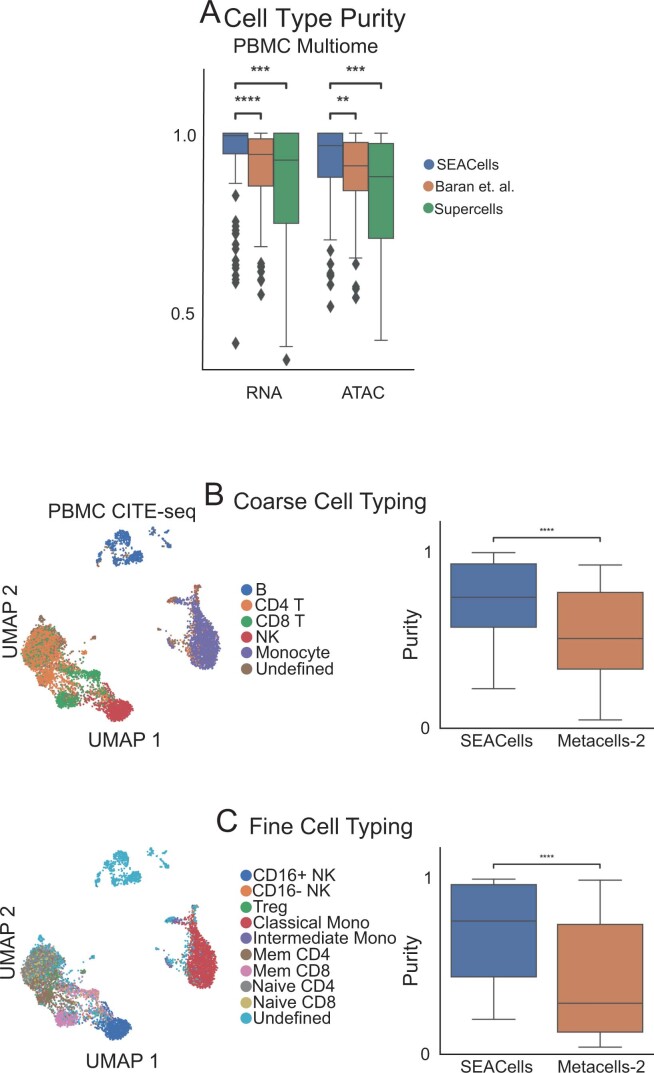
Comparison of RNA metacells to surface protein defined cell-types. **A**. Metacell cell-type purity (fraction of the maximally represented cell-type amongst the cells assigned to a metacell) computed by different methods on PBMC data. Wilcoxon rank-sum test was used to assess the significance of differences (** 0.001 < P < 0.01, *** 0.0001 < P < 0.0001, **** P < 0.0001). **B**. Left: UMAPs of 10x Genomics PBMC CITE-seq dataset^5^, colored by coarse cell-types. Right: Comparison of cell-type purity between SEACells and MetaCell-2 metacells. Metacells were identified using the RNA modality whereas cell types were determined using cell-surface protein profiles. **C**. Same as (A), for finer resolution cell-types. Box plots display median, 25^th^(Q1) and 75^th^ (Q3) percentiles; whiskers extend to the furthest datapoint within the range 1.5 *(Q3-Q1); points beyond that are denoted as outliers. Number of metacells = 109.

We evaluated the purity of well-separated cell types in PMBC data and found that metacells of both modalities show substantially greater purity from SEACells than other methods (Extended Data Fig. 6a). We observed similar differences in PBMC cellular indexing of transcriptomes and epitopes by sequencing (CITE-seq) data^37^, using surface protein measurements as ground truth (Extended Data Fig. 6b,c). Notably, peak accessibility and gene expression are also much better correlated in metacells from SEACells (Supplementary Fig. 9a) than other methods (Fig. 4b and Supplementary Fig. 13).

**Extended Data Fig. 7:**
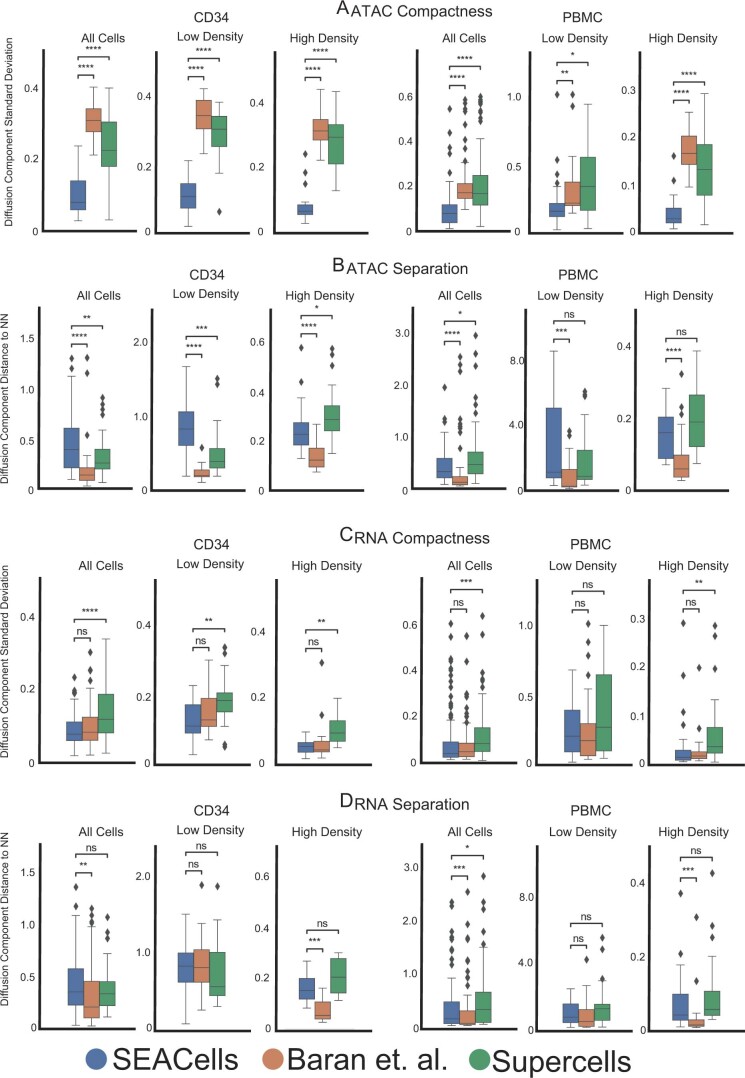
Performance of different approaches in achieving metacell compactness and separation. **A**. Metacell compactness (average diffusion component standard deviation; Methods) measured in the ATAC modality of CD34^+^ and PBMC multiome data. A lower score indicates more compact metacells. Number of metacells = 86 (CD34), 98 (PBMC). **B**. Metacell separation (distance between nearest metacell neighbor in diffusion space; Methods) measured in the ATAC modality of CD34^+^ and PBMC multiome data. Larger separation indicates better performance. Number of metacells = 86 (CD34), 98 (PBMC). **C**. Metacell compactness measured in the RNA modality of CD34^+^ and PBMC multiome data. Number of metacells = 65 (CD34), 98 (PBMC). **D**. Metacell separation measured in the RNA modality of CD34^+^ and PBMC multiome data. Larger separation indicates better performance. Number of metacells = 65 (CD34), 98 (PBMC). Comparisons were carried out on all metacells, or metacells in low-density or high-density regions. Two-sided Wilcoxon rank-sum test; ns: *P* > 0.05, * 0.01 < *P* < 0.05, ** 0.001 < *P* < 0.01, *** 0.0001 < *P* < 0.0001, **** *P* < 0.0001. Box plots display median, 25^th^(Q1) and 75^th^ (Q3) percentiles; whiskers extend to the furthest datapoint within the range 1.5 *(Q3-Q1); points beyond that are denoted as outliers.

An ideal metacell is also compact (it exhibits low variance among constituent cells) and well separated (it remains distant from cells of a neighboring metacell). We defined metrics for compactness and separation and found that SEACells exhibits superior performance in both modalities, for the two benchmarking datasets (Supplementary Note 3, Fig. 4c, Extended Data Fig. 7 and Supplementary Fig. 14). Collectively, our results show that metacells generated by SEACells better represent the catalog of cell states present in the data and are more homogenous, compact and well separated than alternative methods across both RNA and ATAC modalities.

### SEACells reveals accessibility dynamics in differentiation

**Fig. 5:**
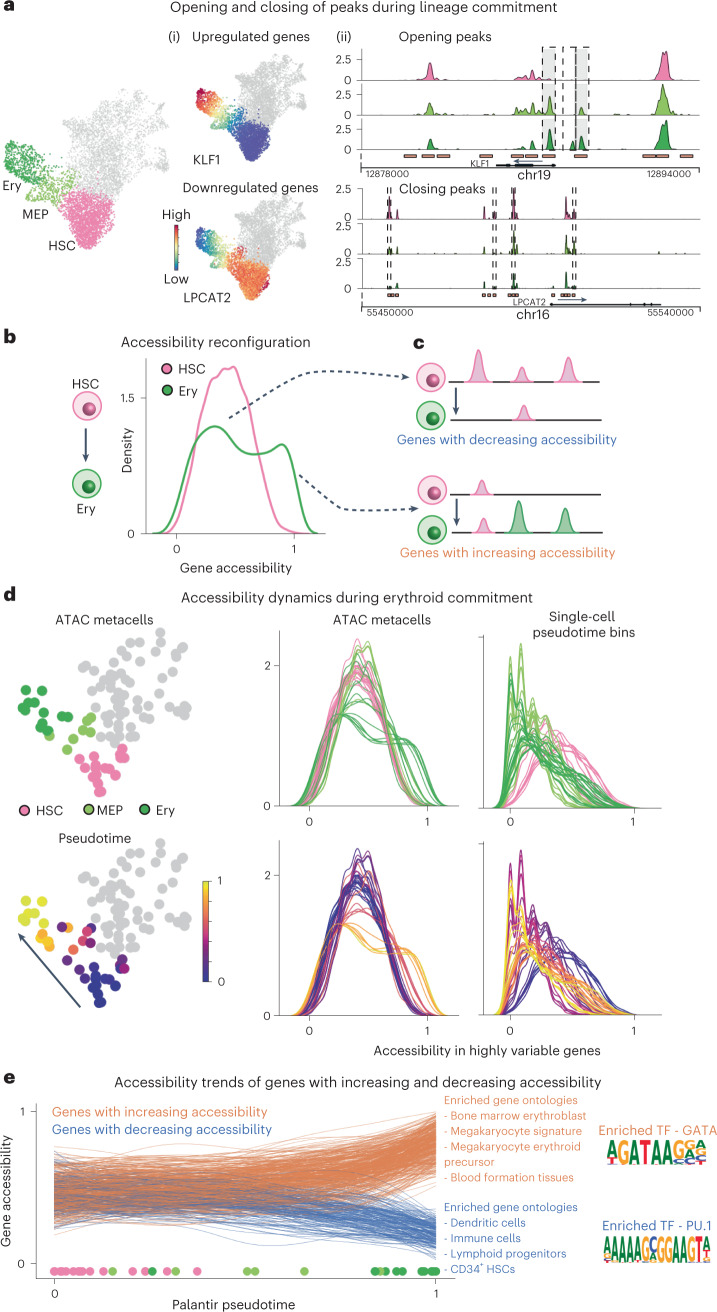
Charting chromatin accessibility of hematopoietic differentiation using SEACells metacells. **a**, Differentiation along a particular lineage involves upregulation of lineage-defining genes and downregulation of stemness genes or genes that define alternative lineages. Left: RNA modality UMAP of CD34 bone marrow, with erythroid lineage cells highlighted. Middle: UMAPs colored by expression of erythroid gene *KLF1* and stem gene *LPCAT2*, which are upregulated and downregulated, respectively, during erythroid differentiation. Right: accessibility landscapes of *KLF1* (top) and *LPCAT2* (bottom), aggregated by cell type, during erythroid differentiation. **b**, Distribution of gene accessibility for all highly regulated genes, for HSCs and for erythroid cells (Ery). Unimodal gene accessibility in HSCs is reconfigured to a bimodal distribution during erythroid differentiation. **c**, Schematic representation of observed peak dynamics. Bimodally distributed gene accessibility results from a subset of genes losing open peaks (top) and another subset gaining open peaks (bottom). **d**, Chromatin accessibility distribution of highly regulated genes in all metacells along the erythroid lineage (left, middle). Each line represents a metacell, colored by its stage (top) and pseudotime (bottom). The emergence of bimodality is gradual and continuous. Right: Signal is poorly defined when using single-cell pseudotime bins rather than metacells. **e**, Accessibility dynamics of genes that gain (orange) and lose (blue) open peaks during differentiation from HSCs to erythroid cells. Trajectory was computed using Palantir, with each line representing a fit gene trend and circles at bottom depicting pseudotime values of each respective metacell. Middle: results of Gene Ontology analysis using immune cell gene signatures. Right: Opening peaks are enriched for GATA motifs, and closing peaks are enriched for PU.1, master regulators of erythroid and myeloid fates, respectively.

**Extended Data Fig. 8:**
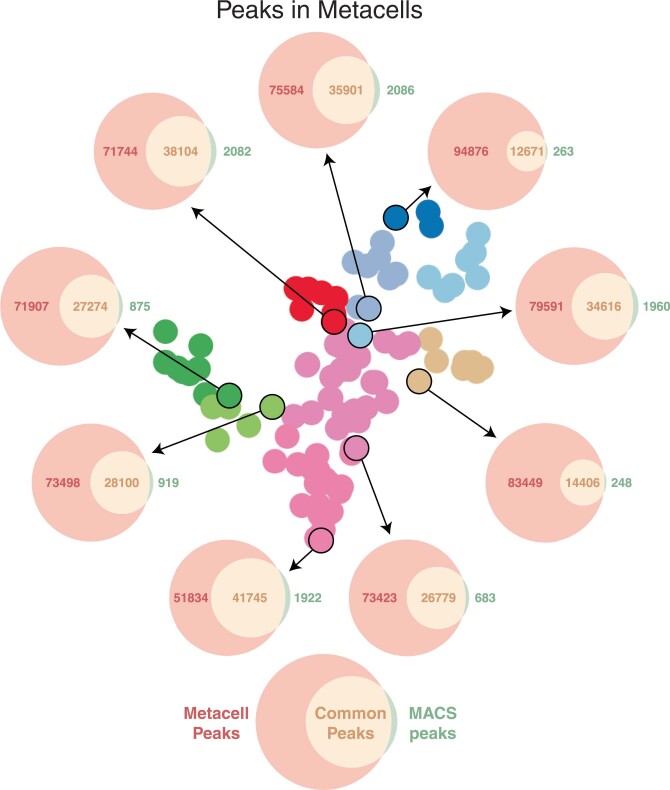
Metacell peak calling. Each Venn diagram depicts the number of peaks in a metacell based on *de novo* peak calling using MACS2 on the metacell ATAC fragments (green), open peaks called using Poisson statistics on ATAC fragments from all cells in the sample (red), and the intersection set (orange). *De novo* peak calling always leads to fewer peaks.

Hematopoietic differentiation is characterized by the upregulation of lineage-defining genes and the downregulation of stemness genes, driven by changes in chromatin accessibility that enable or impede TF binding (Fig. 5a). Stem cells exhibit extensive priming of lineage gene regulatory elements, whereby enhancers are accessible for lineage-specific expression^18,33,34^. We used SEACells to better elucidate how the permissive epigenomic landscape of hematopoietic stem cells (HSCs) dynamically reconfigures to a sharply restricted landscape in differentiated cells. We identified open elements in each ATAC metacell (Extended Data Fig. 8) and then defined the fraction of gene-associated peaks (Methods) that are open in each metacell, from 0 (all peaks closed) to 1 (all peaks open), as a metric of gene accessibility. Our accessibility scores track with gene expression for key lineage-specific genes (Supplementary Fig. 15a,b).

We next examined the accessibility of all highly regulated genes across cell types. HSCs follow a unimodal distribution centered at 0.5, whereas, for differentiating cells, genes that define the cell’s lineage gain peaks, and those defining alternative lineages lose peaks (Fig. 5b,c). The resulting bimodality of differentiated cells is most clearly observed in the erythroid lineage (Fig. 5b). All other lineages exhibit long-tailed distributions (Supplementary Fig. 16a,b), but a similar analysis on unsorted bone marrow mononuclear cells^21^ revealed more pronounced bimodality (Supplementary Fig. 16c,d), indicating that the lack of clear bimodality in other lineages was due to our CD34-sorted data retaining too few mature cells.

We focused on accessibility dynamics in the erythroid lineage. We first applied Palantir^5^ to SEACells metacells using the RNA modality to determine a pseudotime ordering and then examined the accessibility dynamics of highly regulated genes in each metacell along the pseudotemporal order (Fig. 5d and Methods). This analysis reveals that epigenomic reconfiguration is itself gradual and continuous—an observation that is not apparent using single-cell pseudotime bins (Fig. 5d). Moreover, the gradual opening and closing of regulatory elements diverge at lineage-specific loci; genes with increasing accessibility in the erythroid lineage establish erythroid cell identity and function, whereas those with decreasing accessibility are enriched for HSC and diverse other lineage genes, in further support of epigenomic poising in HSCs (Fig. 5e). Finally, the enrichment of TF motifs in peaks gained and lost in erythroid differentiation predicts a role for *GATA2* and *PU.1*, respectively (Fig. 5e and Methods), consistent with the known mutual antagonism of these factors in the decision between erythroid and myeloid lineages^38^.

Our results demonstrate that SEACells metacells enable the modeling of gene accessibility dynamics during differentiation, including the reconfiguration of the hematopoietic chromatin landscape.

### SEACells facilitates single-cell cohort integration

Large consortia are generating single-cell datasets of up to tens of millions of cells and hundreds of individuals^39–44^, which harbor substantial batch effects related to sample and collection site. Despite enormous progress in data integration approaches^45–48^, biological variation between individuals is often impossible to distinguish from technical noise, due in large part to the sparsity of single-cell data. By aggregating highly similar cells into robust, well-defined biological states, metacells provide per-sample summary statistics that better preserve subtle biological differences and distinguish them from batch effects. We used a dataset of over 175,000 PBMC cells from 23 healthy donors and 17 patients with critical Coronavirus Disease 2019 (COVID-19)^49^ to demonstrate how using SEACells metacells as input to data integration offers marked improvements over using single cells.

**Fig. 6:**
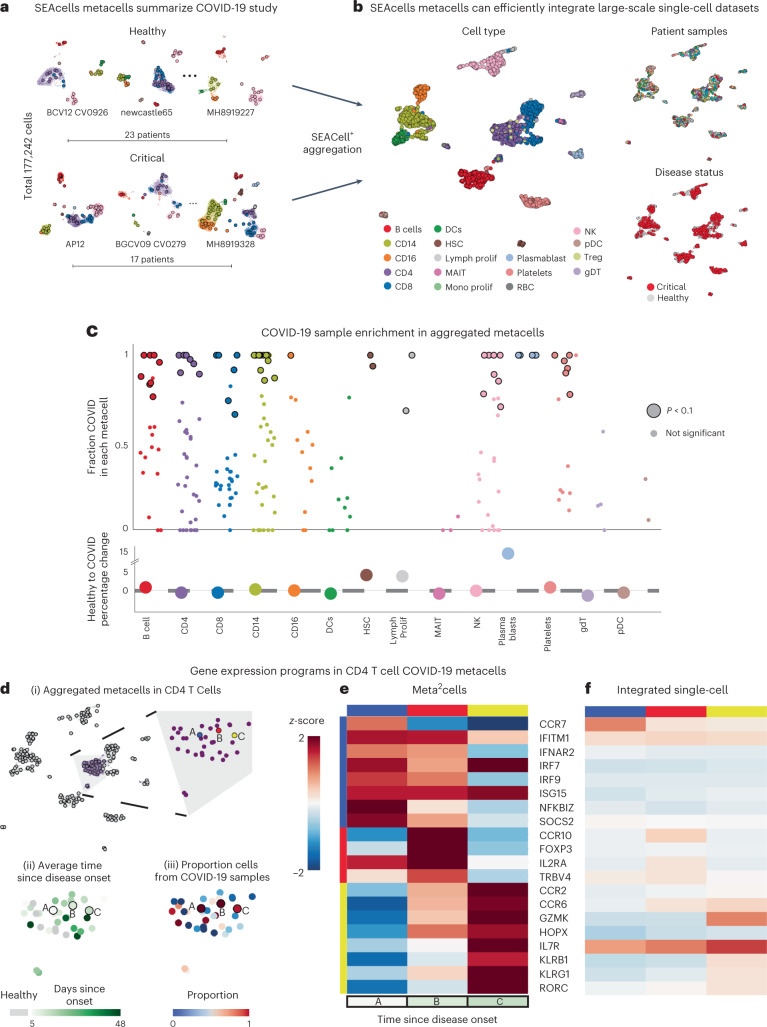
SEACells metacells identify dysregulated states in patients with COVID-19. **a**, UMAPs showing PBMC profiles and respective metacells for a subset of healthy individuals and patients with critical COVID-19 (ref. ^49^). Dots, cells; circles, metacells. Cells and metacells are colored by cell type. **b**, UMAPs showing metacells from different patients integrated using Harmony^45^. Metacells are colored by cell type (left), sample (top right) or disease status (bottom right). **c**, Top: differential abundance of SEACells metacell states in patients with COVID-19 compared to healthy individuals, computed using a permutation test (Methods). Significantly differential metacells are plotted as enlarged circles. Bottom: difference in proportion of cells derived from patients with COVID-19 compared to healthy individuals, analyzed at the cell type level. Nominal *P* value derived from an empirical null distribution. **d**, (i) UMAP of metacell aggregates (meta^2^cells). Right: zoom-in on CD4 T cell meta^2^cells. Three meta^2^cells enriched in patients with COVID-19 compared to healthy individuals are highlighted. (ii) Same as (i), with meta^2^cells colored by time since disease onset. (iii) Same as (i), with meta^2^cells colored by proportion of COVID-19 cells. **e**, Expression patterns of T cell activation and differentiation enriched in highlighted CD4 T cell meta^2^cells. **f**, Expression patterns of the same genes in (iv) derived using pseudo-metacells by aggregating cells in the Harmony^45^ batch-corrected low-dimensional space.

**Extended Data Fig. 9:**
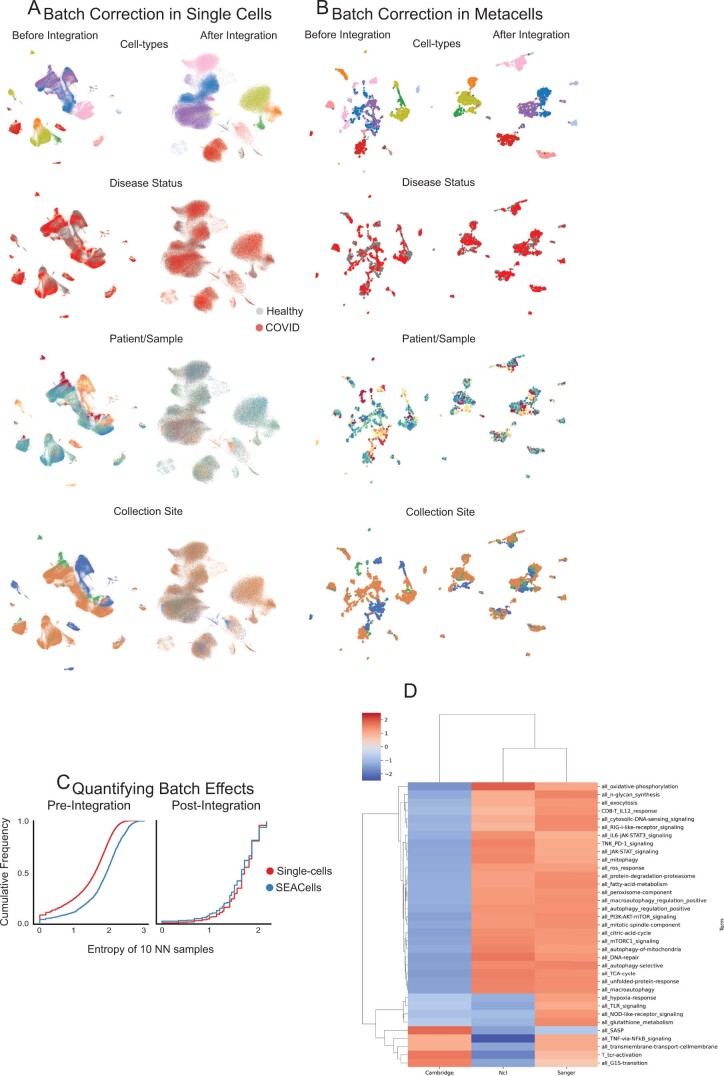
Large-scale data integration using SEACells. **A**. UMAPs of single-cell data from healthy donors and critical COVID-19 patients before (left) and after (right) batch correction and data integration using Harmony^4^. **B**. Same as (A) for SEACells metacells instead of single cells. **C**. Cumulative distribution showing the entropy of samples among 10 nearest neighbors before integration (left) and after integration (right). **D**. GSEA normalized enrichment scores for each T-cell pathway (Supplementary Table 1) for differentially expressed genes among CD4^+^ T cells from different sites of sample collection.

We first identified metacells in each sample (Fig. 6a and Supplementary Fig. 17) and verified that metacell states are consistent across healthy donors and across patients with COVID-19 (Supplementary Fig. 18 and Methods). We used metacell gene expression counts for downstream data integration^45^, clustering^50^ and uniform manifold approximation and projection (UMAP) visualization (Fig. 6b). Sample-level batch effects are severe before integration but substantially lower in metacells compared to single cells (Extended Data Fig. 9). Although data integration eliminated sample-level batch effects in both single cells and metacells (Extended Data Fig. 9c), the site of sample collection, originally noted as a severe technical artifact^49^, remained a strong confounding variable in metacells, particularly in CD4^+^ T cells (Extended Data Fig. 9b). To investigate, we examined differential expression between CD4^+^ T cell metacells collected at different sites and observed coherent biological responses relevant for these cell types, supporting the existence of meaningful biological differences between sites that should not be removed (Extended Data Fig. 9d and Methods). We note that each site collected samples at different timepoints in disease progression, providing a likely explanation for the observed biological differences and demonstrating that SEACells preserves biological signal in the presence of substantial technical noise.

Metacells also improve the computational efficiency of analyses, such as dimensionality reduction and clustering, which are rapidly becoming infeasible for very large single-cell datasets. We applied SEACells across the entire COVID-19 atlas^49^, spanning 119 samples and more than 600,000 cells from healthy controls and diverse COVID-19 stages (Supplementary Fig. 19 and Methods). This dataset was summarized by ~8,000 metacells, which exhibit high cell type purity (Supplementary Fig. 19b,c), and required orders of magnitude less compute time than computation at the single-cell level. Scalability is particularly important when existing analyses need to be rerun to incorporate new data; a one-time investment in metacell assignment avoids compounding the near-exponential increases in runtime associated with adding cells, for each single-cell-level analysis (Supplementary Fig. 20).

### SEACells identifies T cell response dynamics in COVID-19

**Extended Data Fig. 10:**
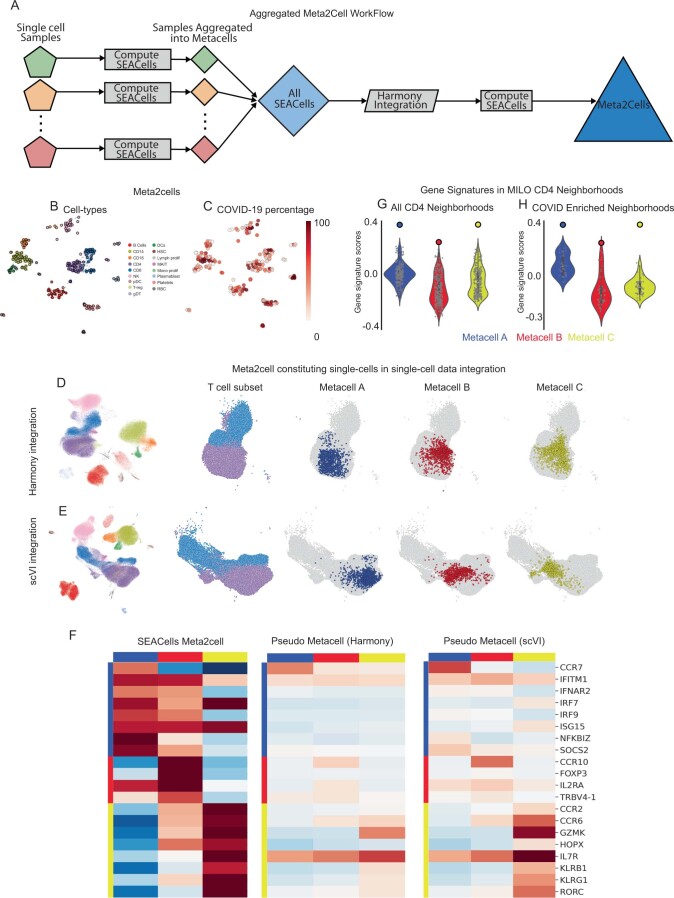
Metacell aggregates enable comparison across conditions. **A**. Workflow used to generate metacell aggregates, or meta^2^cells, that span across healthy and COVID-19 samples. **B**. UMAPs showing meta^2^cells, colored by cell type. **C**. Same as (B), colored by percentage of cells derived from COVID-19 patients. **D**. Left: Single-cell UMAPs of the healthy and COVID-19 samples following Harmony^4^ integration. UMAPs on the right illustrate the UMAP region with the T-cell subsets and the cells constituting meta^2^cells highlighted in Fig. 6D. **E**. Same as (D), with UMAPs derived using scVI^5^ latent space. **F**. Expression patterns of key T-cell defining genes in meta^2^cells from Fig. 6D, pseudo-metacells derived using Harmony and scVI. Pseudo-metacells were derived using a neighborhood of 1078, 1161, and 1119 cells for metacells A, B and C respectively from the median position of batch-corrected low-dimensional space (see Methods). **G, H**. Gene signature scores for the CD4^+^ metacells in Fig. 6D. Violin plots represent signature scores for Milo neighborhoods, solid dots represent scores for the SEACells metacells. Scores are computed for all CD4^+^ T-cell Milo neighborhoods (G) and for the subset of neighborhoods enriched in COVID-19 (H). Number of metacells = 276. Violin plots display median, 25^th^(Q1) and 75^th^ (Q3) percentiles; whiskers extend to the furthest datapoint within the range 1.5 *(Q3-Q1); points beyond that are denoted as outliers.

We next examined whether SEACells can help identify state changes between healthy donors and patients with severe COVID-19. We pooled metacells from all donors and re-applied SEACells to derive metacell aggregates, or ‘meta^2^cells’, representing states across all samples (Extended Data Fig. 10a–c). Each meta^2^cell is a combination of healthy and COVID-19 metacells, such that the fraction of COVID-19 cells can be visualized for each state. Our results reveal a spectrum of metacell states, from those specific to healthy donors to those exclusive to COVID-19 (Extended Data Fig. 10c), prompting us to develop a permutation test to identify cell states that differ significantly between conditions (Fig. 6c and Methods). Analysis at the cell type level, by contrast, masks the extensive heterogeneity in individual states (Fig. 6c).

We focused on CD4^+^ T cells, which differentiate into distinct subsets upon activation and differentiation^51,52^, using differential gene expression analysis at the metacell level to identify cell-state-defining genes. Within CD4^+^ T cell meta^2^cells, this analysis revealed a fine-grained trajectory of phenotypes enriched in patients with critical COVID-19, with T cell phenotypes that correspond meaningfully with disease stage (Fig. 6d,e). For example, a meta^2^cell enriched in patients soon after infection (metacell A) contains cells in an early activation state distinguished by the expression of NF-κB response genes, IFN-α receptor subunit *IFNAR2* and downstream interferon-stimulated genes (*IRF7*, *IRF9*, *ISG15* and *IFITM1*), reflecting T cell responsiveness to type I IFN, a cytokine associated with viral infections and severe acute respiratory syndrome coronavirus 2 (SARS-CoV-2) pathology^53^ (Fig. 6e). A meta^2^cell enriched in patients with COVID-19 approximately 10 days after symptom onset (metacell B) comprises Foxp3^+^ Treg cells expressing the chemokine receptor gene *CCR10*, suggesting recruitment to the inflamed lung or mucosal epithelium and a role in regulating inflammation^54^ (Fig. 6e). Finally, a meta^2^cell enriched in patients with persistent severe COVID-19 at day 13 (metacell C) contains cells that express hallmark T_H_17 genes (*RORC* and *CCR6*), reflecting a shift toward type III inflammation. Aggregated metacell states are, thus, highly consistent with the known temporal dynamics of gene expression during T cell response to infection.

By contrast, single-cell data integration did not preserve neighborhoods that constitute CD4^+^ T cell metacells or recover the signal for disease progression (Extended Data Fig. 10d,e). Furthermore, aggregating cells in batch-corrected low-dimensional embeddings (Methods) did not produce the characteristic expression patterns of disease-associated CD4^+^ T cell metacells (Fig. 6f and Extended Data Fig. 10f). Differential abundance testing^55^ at the single-cell level also failed to recover these dynamics (Extended Data Fig. 10g,h). Our results demonstrate that SEACells can capture biologically meaningful CD4^+^ T cell subsets, highlighting the transition from the spectrum of active to quiescent differentiated states during a multi-day viral infection. We postulate that, although data integration methods aim to make samples more similar without distinguishing batch from biological signal, aggregating data into metacells on the per-sample level provides robust capture of true biological variation between samples. SEACells can facilitate the development of integration approaches that use the summary statistics encoded in metacells to better distinguish biological signal from technical noise.

## Discussion

SEACells identifies robust, reproducible metacells that overcome sparsity while retaining the rich heterogeneity of single-cell data. SEACells metacells are more compact, better separated and more evenly distributed across the full cell state landscape than metacells generated by existing methods. They greatly improve integration across samples and scaling analysis to large cohort-based datasets. Critically, only SEACells is currently able to derive cell states from scATAC-seq data in an accurate and comprehensive manner, greatly empowering gene regulatory inference.

SEACells performance is due to (1) its representation of single-cell phenotypes using an adaptive Gaussian kernel to accurately capture the major sources of variation in the data; (2) its use of maximum–minimum sampling for initialization to ensure even representation of cell states across phenotypic space, regardless of cell densities; and (3) its application of kernel archetypal analysis for identifying highly interpretable metacells. The adaptive kernel and maximum–minimum sampling make SEACells particularly adept at robustly identifying rare cell states, which often represent critical populations that drive biology or disease.

Whereas gene scores, open regulatory elements and correlations between gene expression and chromatin accessibility cannot be determined robustly at the single-cell level, they can be computed for individual metacells. Such improvements in fundamental ATAC analysis, which currently occurs at the cluster level due to extreme sparsity, greatly empower our ability to infer top regulators driving differentiation and enable more sophisticated regulatory network inference, promising wide utility for SEACells metacells in single-cell chromatin profiling data.

SEACells provides a scalable solution for integrating large datasets from cohorts. Metacells can be computed separately for each sample, rendering the integration of additional cohort members resource efficient. Despite considerable progress, current integration approaches are not equipped to distinguish batch effects from subtle biological differences between individuals. Computing metacells at the sample level provides a more robust representation of sample-specific biology, thus serving as better input for data integration. The development of approaches to estimate gene–gene covariances of the dozens of cells within each metacell will help to define metacells as parameterized distributions and spur the development of data integration methods that use this information. As the COVID-19 data demonstrate, sample-level sufficient statistics provided by SEACells are well suited to compare disease states between healthy and normal as well as more nuanced disease states, such as progression. SEACells identified COVID-19-enriched CD4^+^ T cell states that are removed by typical batch correction and undetected at the single-cell level. SEACells metacells serve as robust cell state inputs that facilitate the distinction of biological signal from batch effect—features that enabled our discovery of the T cell state continuum.

## Methods

### SEACells algorithm

SEACells is an algorithm for defining metacells—groups of cells that represent singular cell states—from single-cell data. The SEACells algorithm assumes that biological systems consist of well-defined and finite sets of cell states defined by covarying patterns of gene expression. Observed single-cell data are assumed to be sparse and noisy measurements of these cell states (current state-of-the-art single-cell measurement technologies can capture only <10% of transcripts or <5% of open chromatin regions). Despite the high degree of noise, cells sampled from the same states are assumed to have closely related phenotypes, due to gene expression patterns and regulatory mechanisms that define the cell states. SEACells aims to aggregate closely related cells into metacells that represent them, thereby overcoming single-cell data sparsity. scATAC-seq data are particularly limited in utility due to sparsity. SEACells metacells also provide a scalable representation that efficiently handles large-scale single-cell data. Although clustering is widely used to overcome sparsity, clustering masks the substantial heterogeneity present in the data (Fig. 1a–d). SEACells metacells achieve a resolution that retains heterogeneity while overcoming the sparsity of single-cell data.

The inputs to SEACells are (1) raw count matrices (for example, transcript counts for RNA, peak or bin counts for ATAC); (2) a low-dimensional representation of the data derived using modality-appropriate pre-processing, such as principal component analysis (PCA) for RNA; and (3) the number of metacells to be identified. As output for downstream analyses, SEACells produces groupings of cells that represent metacells, aggregated metacells-by-feature raw counts matrices and soft assignments representing groups of highly related cells.

The algorithm is freely available at https://github.com/dpeerlab/SEACells, in a repository that includes documentation and tutorials for computing metacells and gene expression—peak accessibility correlations, ATAC gene scores, open peaks in metacells, gene accessibility scores and TF activity inference using multiome or integrated RNA and ATAC data.

SEACells comprises five main steps, which are summarized below and elaborated in the following sections.

1. Construct a *k*-nearest neighbor (KNN) graph using Euclidean distances between cells, computed in the lower-dimensional embedded space, to represent the phenotypic manifold.
2. Derive an affinity matrix of cell-to-cell similarities using the nearest neighbor graph. Distances in the graph are transformed to similarities using an adaptive Gaussian kernel to account for the considerable cell density differences in a typical phenotypic manifold^56^. The affinity or kernel matrix (Fig. 1f) encodes the non-linear relationships between cells.
3. Use the kernel matrix as the input for archetypal analysis (Fig. 1g and Extended Data Fig. 1a). Whereas archetypal analysis has typically been applied to the data matrix, we apply it to our kernel matrix, which partitions cells into clusters of highly similar cells and enables the characterization of the entire phenotypic manifold, making it ideally suited to identify robust cell states (Extended Data Fig. 1b–e). Archetypal analysis decomposes the data into an archetype matrix comprising linear combinations of cells that represent cell states on the phenotypic manifold and a membership matrix that reconstructs single cells as linear combinations of archetypes (Fig. 1g and Extended Data Fig. 1a). This procedure partitions the data in such a way that the cell–cell similarity matrix has tight block structure along the diagonal; each partition is a group of cells that best represents a cell state and defines a metacell. The number of metacells is specified as an input to archetypal analysis.
4. Label groupings identified through archetypal analysis as SEACells metacells and aggregate single-cell raw counts accordingly to derive a metacell-by-feature count matrix.
5. Normalize count matrices, which can be used for all downstream single-cell analytical tasks, such as clustering, visualization, data integration, trajectory inference and ATAC-seq-based regulatory inference.

#### Nearest neighbor graph construction

##### Low-dimensional embedding

SEACells requires a low-dimensional representation of single-cell data and uses the Euclidean distance between cells in this embedding to construct the KNN graph. Neighbor graphs are typically computed in lower-dimensional embeddings of single-cell data owing to their extreme sparsity and noise, which results from low molecule capture rates. We recommend the use of PCA for scRNA-seq and singular value decomposition (SVD) for scATAC-seq, as is standard in the field. More generally, a low-dimensional embedding can be derived by using appropriate pre-processing and normalization steps for the data modality of interest (Supplementary Fig. 2). This allows us to be both flexible to data type and robust to the extensive degree of sparsity and noise in data types, such as scRNA-seq and scATAC-seq. We used the following pre-processing steps adapted to the characteristics of each technology.

##### PCA for scRNA-seq

Following standard practice, we perform three main pre-processing steps using the scanpy^57^ package: (1) normalize library size by dividing raw counts by total molecules per cell; (2) log-transform with a pseudocount of 0.1; and (3) select highly variable genes. Based on our previous observations for PBMCs and CD34^+^ bone marrow datasets, we chose 2,500 highly variable genes for analysis. This number should be adapted to ensure that all the heterogeneity is captured in the dataset of interest. Principal components (PCs) are computed from these highly variable genes, with the number of PCs being selected based on proportion of variance explained (typically 50).

##### SVD for scATAC-seq

We used the ArchR package^24^ to pre-process scATAC-seq data. Fragment counts for each cell were computed in 500 base genome bins and normalized using TF-IDF^58^, and SVD was applied to normalized counts to derive a low-dimensional embedding. Like PCA, the number of components was selected based on the proportion of variance explained (typically 30). As previously observed^24^, despite normalization, the first SVD component shows high correlation with number of fragments per cell (correlation > 0.97) and is excluded from downstream analysis.

##### Nearest neighbor graph

A KNN graph is constructed using Euclidean distance in the low-dimensional embedding (PCA or SVD), with single cells represented by nodes that are each connected to their most similar neighbors. The nearest neighbor graph can be represented as a matrix *D* ∈ *R*^*n X n*^, where *n* is the number of cells. *D*_*ij*_ represents distance between cells *i* and *j* if they are neighbors and *D*_*ij*_ = 0 otherwise. The graph serves as input for constructing the cell–cell kernel matrix. As default, 50 neighbors are used for the KNN graph, and we previously demonstrated that the kernel matrix construction is robust to a reasonable range of number of nearest neighbors^4^.

##### Other single-cell data types, including multimodal data

The procedures for computing peak–gene associations, gene scores and gene accessibility assume the availability of either multimodal data or integrated RNA and ATAC modalities. Several approaches have been developed for data integration across modalities^12,59^, and the low-dimensional representations derived using multimodal data can be used to compute SEACells metacells. Given the kernel representation, SEACells can also be applied to other modalities, such as CUT&Tag^60,61^, or other single-cell chromatin modification measurements^62^ with appropriate pre-processing. All that is required is a reliable distance metric between cells, which can be Euclidean distance in alternative embeddings.

#### Construction of the affinity kernel matrix

We transform the *distances* in the neighbor graph to *similarities* between neighboring cells. Gaussian kernels provide a typical approach for this transformation but assume that densities in underlying data are approximately uniform. Single-cell data, however, show remarkable variability in data densities (Supplementary Fig. 7), and low-density regions or rare cell types, such as stem cells, often represent the most meaningful biology. We previously demonstrated that an adaptive kernel using neighbor distance as the scaling factor for each cell, rather than a fixed parameter, represents phenotypic similarities very faithfully^15,16^ and, thus, employ an adaptive (width) Gaussian kernel to determine similarities between cells in SEACells (Fig. 1f). The adaptive kernel corrects for densities using the distance to the *l-*th (*l* < *k*) nearest neighbor as a scaling factor—that is, the scaling factor of cell *i* is given by *σ*_*i*_ = distance to *l-*th nearest neighbor.

The adaptive Gaussian kernel is then given by

$$M\left( {x_i,x_j} \right) = \frac{1}{{\sqrt {2\pi \left( {\sigma _i + \sigma _j} \right)} }}exp\left( { - \frac{1}{2}\frac{{\left( {x_i - x_j} \right)^T\left( {x_i - x_j} \right)}}{{\sigma _i + \sigma _j}}} \right)$$

where *x*_*i*_ is the low-dimensional embedding corresponding to cell *i*—that is, PCA for scRNA-seq and SVD for scATAC-seq. *M* ∈ *R*^*n X n*^ is the affinity matrix. *M*_*ij*_ represents the similarity between cells *i* and *j* if they are mutual neighbors and *M*_*ij*_ = 0 otherwise, and *n* is the number of cells. In other words, the Gaussian kernel transforms the cells from low-dimensional space (dimension = *n* × *p*) to a kernel space (dimension = *n × n*) such that cells are both observations and features, and the ‘phenotype’ of an observation (cell) is defined by the neighborhood similarity structure of that cell in the original low-dimensional space.

In this kernel space, two cells (*x* and *y*) are embedded close to each other if they satisfy two conditions:

1. *x* and *y* share neighbors in the PCA space
2. the similarity scores among the neighbors of *x* and *y* are similar

Two cells in this transformed dimensional space will be similar to each other only if they share neighbors *and* the distances to the shared neighbors are similar, imposing stricter similarity conditions between cells.

#### Kernel archetypal analysis

##### Overview and optimization function

The adaptive Gaussian kernel matrix, $M \in R^{n\;X\;n}$, serves as input to archetypal analysis. Archetypal analysis^16^ performs a linear decomposition of the kernel matrix. The goal is to identify a specified number of archetypes, each of which is a linear combination of the data points represented by the archetype matrix (matrix B in Fig. 1g and Extended Data Fig. 1a). The data points themselves are represented as a linear combination of the archetypes in a membership matrix (matrix A in Fig. 1g and Extended Data Fig. 1a) to reconstruct the kernel matrix. The number of archetypes is substantially lower than the number of data points, and the lower dimensionality of the archetype and membership matrices creates an information bottleneck, ensuring an optimal decomposition of the data^15^. The weighted assignments of cells to archetypes are contained in the membership matrix, which can be used to derive cell partitions that are aggregated to metacells (Fig. 1h). The linear nature of archetypal analysis ensures maximal interpretability and identification of metacells.

Archetypal analysis decomposes the kernel matrix as *M* ≈ *ZA*—that is, the kernel matrix *M* is represented as a convex combination of a latent archetype matrix $Z \in R^{n \times s}$ and cell membership matrix $A \in R^{s \times n}$, where $s \ll n$ is the number of archetypes. As these latent archetypes are unknown a priori, they are themselves defined as convex combinations *Z* = *MB* of the kernel matrix, *M* and archetype weight matrix $B \in R^{n \times s}$. To ensure that data points are convex combinations of archetypes, and vice versa, weight matrices *A* and *B* must be column-stochastic, such that their entries are non-zero and columns sum to 1.

Formally, for entries $a_{ij} \in A$ and $b_{ij} \in B$,

$$a_{ij} \ge 0,\;\;\forall \;j = 1...n\mathop {\sum}\limits_{i = 1}^s {a_{ij} = 1}$$

$$b_{ij} \ge 0,\;\;\forall \;i = 1...n\mathop {\sum}\limits_{j = 1}^s {b_{ij} = 1}$$

Taken together, the objective of archetypal analysis is to find matrices *A*, *B*, such that product *MBA* forms a faithful reconstruction of the original kernel matrix *M*.

The objective of kernel archetypal analysis is to minimize squared reconstruction error (SRE) as follows:

$$\begin{array}{l}\mathop {{min}}\limits_{A,B} SRE = \left\| {M - MBA} \right\|^2 = tr\left[ {M^TM - 2M^TMBA + A^TB^TM^TM} \right]\\ \end{array}$$

The number of archetypes, *s*, representing the number of metacells, is a parameter. See Supplementary Note 1.1 for intuition on why kernel archetypal analysis best is suited for metacells.

##### Optimization algorithm for metacell identification

Archetypes are an approximation of the convex hull—that is, they represent the vertices of a convex polytope that encapsulates most of the data (Extended Data Fig. 1e). As a linear combination of data points, archetypes do not necessarily represent measured data points themselves, and each cell is expressed as a linear combination of the inferred archetypes (Fig. 1g). To aid interpretability and facilitate downstream analysis, metacells are constructed by (1) computing binarized assignments of cells to archetypes (of the *A* matrix) and (2) aggregating single cells assigned to each metacell by summing over raw counts (Fig. 1g). This summarized metacell matrix is significantly less sparse and noisy and can be used for more robust downstream analysis.

The objective function for kernel archetypal analysis involves optimizing the non-convex product *AB* and, thus, has many local minima. The objective function is, however, convex in *A* given a fixed *B* matrix and vice versa. Therefore, alternating minimization of weight matrices *A* and *B* is used to make the problem of solving archetypal analysis more tractable. Given this, we use the Frank–Wolfe updates to optimize each weight matrix in turn, as described in ref. ^15^.

##### Initialization

As archetypal analysis is a non-convex problem, solutions depend on the initialization of archetype and cell assignments^16^. Given the density differences in the phenotypic manifold, random sampling of cells will lead to significant overrepresentation of initial points in the high-density regions and severe underrepresentation of cells in the biologically critical low-density regions. Therefore, we employ maximum–minimum sampling of waypoints, as previously described and implemented^4^, to initialize archetypal analysis. Given a set of waypoints, each additional waypoint is chosen to maximize the distance to the current set—that is, maximize the minimum distance to any of the points in the current set. This ensures that waypoints are uniformly distributed across the phenotypic manifold irrespective of density (Fig. 1e). We first derive a diffusion map embedding using the adaptive Gaussian kernel *M*. As previously demonstrated^4^, each diffusion component (DC) represents an axis of biological variation in the data. Waypoints are sampled from each component and pooled for initialization. The number of components can be chosen by the eigengap statistic, although, in practice, we observed that the first ten DCs typically account for biological variability in the data.

Because maximum–minimum sampling is performed using each DC, there can be redundancy in the cells sampled (that is, the same cell may be sampled for multiple components). Therefore, a pre-specified proportion of waypoints (less than or equal to 1) is selected by maximum–minimum sampling, and the remaining are computed using a greedy column subset selection approach^63^. The column subset selection is a fast and greedy algorithm that seeks to identify representative columns from a large dataset by minimizing an objective function, which measures reconstruction error of the data matrix. Thus, SEACells is initialized by selecting cells that are more likely to be representative of other cells in the dataset.

Waypoints are used to initialize the matrix *B*, after which matrix *A* is updated, and the process is repeated until convergence (see Supplementary Note 1.2 for convergence criteria).

#### Metacell construction

##### Analysis of metacell assignment certainty

Metacells are identified by binarizing the assignment matrix *A*. Cell assignment weights are determined by first zero-ing out ‘trivial’ assignment weights (< 0.05) as a form of regularization and then normalizing the weights for each cell. The proportion of cells with maximal assignment weight less than 0.5 (gray), between 0.5 and 0.8 (red), between 0.8 and 0.9 (yellow) and, finally, greater than 0.9 (green) are shown in Supplementary Fig. 5. The overwhelming majority of cells have highs confidence assignments.

#### Metacell annotation and normalization

Metacells are annotated as belonging to the most frequent cell type among the constituent cells. Metacell raw counts can be normalized in a manner analogous to single-cell data normalization. Metacell counts are divided by the total counts per metacell and then multiplied by the median of the total metacell counts to avoid numerical issues. The data are then log-transformed using a pseudocount of 0.1.

##### Note about number of metacells

The number of metacells is specified as a SEACells parameter. We have determined that SEACells is robust to a wide range of number of metacells (Supplementary Fig. 6a,b). We currently use a heuristic default of one metacell per 75 single cells in the dataset under consideration. However, the appropriate number is largely dependent on biological structure in the data. For example, a dataset profiling 10,000 cells from a homogeneous cell line will be expected to encode less biological structure than a similar-sized dataset from a more complex biological system, such as a tumor or differentiating tissue. Thus, we recommend examining initialization to ensure that cell states span the entire phenotypic manifold. An additional heuristic, which can be used after optimization, is the number of metacells associated with each cell (with non-trivial weight). Ideally, each cell should be strongly associated with only one or two others, except in the case of highly continuous cell state trajectories. When a surplus of metacells is specified, the number of cells partially assigned to multiple metacells increases (Supplementary Fig. 6c–f). This distribution can be examined for a possible need to reduce the number of metacells and has been implemented as a function in the SEACells GitHub package.

### Toolkit for scATAC-seq analysis

A broad array of powerful tools has been developed for interpreting open chromatin data from bulk ATAC-seq data. However, these tools cannot be applied directly to single-cell data because of their sparsity. SEACells metacells are aggregates of tightly related cells and are, thus, substantially less sparse while faithfully retaining the heterogeneity and structure of the data. Here we describe a robust toolkit for scATAC-seq data adapted from bulk data analysis tools.

#### Peak calling

Peak calling was performed using ArchR^24^. ArchR first clusters single-cell data and uses the MACS2 peak caller^64^ to identify peaks separately for each cluster. Each peak is then resized to 500 bases with the peak summit at the center, and overlapping peaks across different clusters are merged. The merged peaks are again resized to 500 bases.

ATAC-seq data provide a profile of open chromatin regions spanning TF binding regions and nucleosomes in non-repressed regions. The fragment size distribution of ATAC-seq data contains characteristic modes that reflect the diversity of this information (Supplementary Fig. 8a). Because the first mode represents NFRs, we altered the ArchR pipeline to identify peaks using only the NFR fragments (fragment length < 147) rather than use the default of all fragments. This change leads to substantially more sensitive identification of regulatory elements (Supplementary Fig. 8b,c).

The modified ArchR pipeline is available at https://github.com/peerlab/ArchR.

#### Peak–gene associations and gene scores

Although the use of NFR fragments improves the sensitivity of called peaks, not all identified peaks represent TF binding events that regulate gene expression (for example, structural factors such as CTCF also contribute to ATAC-seq signal). Studies have proposed using the correlation of peak accessibility and gene expression from multiome or integrated ATAC and RNA data to identify peaks that likely regulate the expression of the gene^20^. SEACells metacells provide an ideal resolution to compute these associations, which are unreliable when computed using sparse single-cell data. We use metacells identified using the ATAC modality for building the peak–gene associations.

We adopted the procedure outlined by Ma et al.^20^ to identify significant peak–gene associations. For each gene, Pearson correlations were computed for each peak within 100 kb upstream and 100 kb downstream of the gene, using the normalized metacell expression and normalized ATAC accessibility. To assess the significance of the peak–gene correlation, an empirical background of 100 peaks was sampled that matched the GC content and accessibility of the peak under consideration. Peaks were binned into 100 bins separately based on GC content and accessibility to sample the empirical background. Any peak with nominal *P* < 1 × 10^−1^ was considered a significant peak–gene association. Peaks identified using NFR fragments were used for this analysis. The aggregate accessibility of all peaks associated with a gene was used to determine the metacell gene score.

For single-cell comparisons, normalized single-cell expression and normalized single-cell accessibility were used for determining peak–gene associations. Gene scores for single-cell ATAC were computed using the ArchR defaults.

#### Inference of TF activity using metacells

To use the peak–gene associations, we provide a simple gene regulatory network (GRN) approach for TF activity inference, used to identify key TFs that relate to different cell types (Fig. 3c,d, Extended Data Fig. 4 and Supplementary Fig. 10).

FIMO^65^ was used for motif identification in all peaks, based on the cisBP human v2 motif set^66^. For each gene *g*, we identified the subset of peaks, *Sg*, whose accessibility correlates with expression of a proximal gene (*P* < 0.1, correlation > 0.1), using SEACells metacells. We then constructed a TF–target matrix $G \in R^{n\;X\;m}$, where *n* is the number of genes, and *m* is the number of TFs using the FIMO scores within gene–peak correlations (Fig. 3c). Specifically for a gene *g* and TF *t*, we define

$$G_{gt} = \frac{{\mathop {\sum}\nolimits_{k \in Sg} {c_{kg} \ast F_{kt}} }}{{\mathop {\sum}\nolimits_{k \in Sg} {c_{kg}} }},$$

where *c*_*kg*_ is the Pearson correlation between accessibility of peak *k* and gene *g* across all metacells, and *F*_*kt*_ is the FIMO score for transcription *t* in peak *k*. The TF–target matrix *G* is used to infer TF activities using lasso regression (Fig. 3c). Specifically, we use lasso regression^26^ to predict the expression profile of each metacell along the lineage as a function of *G*:

$$min_{w_s}\mathop {\sum}\nolimits_g {\left( {y_g - w_s \cdot G_g} \right)} ^2 + \lambda \mathop {\sum}\nolimits_t {\left| {w_{st}} \right|} ,$$

where $y_g \in R^{nX1}$ is the expression profile of a metacell; $w_s \in R^{1Xm}$ is the inferred vector of TF weights for metacells *s*; and *λ* is the regularization parameter. The regularization parameter is chosen by ten-fold cross-validation. The L1 penalty of lasso regression pushes most of the TF coefficients to 0 and has been extensively used in previous studies to identify regulators of gene expression^27,67,68^.

We then computed TF activities using the lasso regression coefficients. Specifically, we computed a TF activity matrix $M \in R^{m\;X\;s}$, where *s* is the number of metacells, as follows:

$$\begin{array}{l}M_{ts} = \left( \mathop {\sum}\nolimits_g \left( {y_g - \mathop {\sum}\nolimits_{i = 1..m} {\left( {w_{si} \ast G_{gi}} \right)} } \right)^2 \right.\\\left.- \mathop {\sum}\nolimits_g {\left( {y_g - \mathop {\sum}\nolimits_{i = 1..m\;s.t\;i < > m} {\left( {w_{si} \ast G_{gi}} \right)} } \right)^2} \right) \ast sign\left( {w_{st}} \right)\end{array}$$

In other words, TF activity is measured as the increase in the mean prediction error when the TF is excluded from the inferred model. The activity is weighted by the sign of the coefficient to indicate directionality of regulation (positive means upregulation and negative means downregulation of targets). Our previous studies demonstrated that the scores inferred using prediction error are more representative of TF activities than the regression coefficients themselves because each TF has a variable number of targets^27^.

#### chromVAR using SEACells metacells and single cells

chromVAR^30^ is a widely used tool for predicting TF activity from scATAC-seq data. It provides a per-cell deviation score for a TF by computing whether the peaks predicted to contain its binding motif have greater accessibility compared to a GC-matched background peak set. The algorithm was run using default parameters and the chromVAR ‘human_pwms_v2’ motif database. chromVAR scores were computed using aggregated fragment counts for metacells and single-cell fragment counts for single-cell data. Similarly to the single-cell data analysis, chromVAR scores were first reduced to 50 PCs using knee-point analysis. PCs then served as input to UMAPs for visualization.

#### Metacell peak calling

Identification of the set of open regulatory elements is practically implausible at single-cell level due to noise and sparsity. SEACells metacells, however, provide enough fragments per cell state to enable the identification of open regulatory elements in each state. We observed that de novo peak calling in each metacell results in loss of sensitivity (Extended Data Fig. 8). Therefore, we use the peaks identified by ArchR across all cells as an atlas to determine the subset of peaks open in each metacell.

A procedure inspired by MACS2 is used to identify open regulatory elements in metacells because the peaks themselves were called by MACS2. The fragments mapping to peaks are modeled as a Poisson distribution. The mean of the Poisson distribution for a metacell *s* is estimated using^64^

$$\lambda = \frac{{Width\left( {peaks} \right)\; \ast \;Total\;fragments\;in\;s}}{{Effective\;genome\;length }}$$

Because all the widths are identical, the first term of the numerator is set to 500. Rather than use the whole genome length as the denominator, *effective genome length* was set to be *num. of peaks × 5,000*, a more stringent local estimate of the mean as proposed in MACS2. For a peak *p* in metacell *s* with *n* fragments, *λ* is used to estimate the *P* value of observing more than *n* fragments, and *p* is considered open in *s* if *P* < 1 × 10^−2^.

We noticed that some of the ATAC metacells had low overall fragment counts; therefore, we computed fragments per peak and total fragments from the two nearest metacells. We apply this procedure for all metacells to avoid any biases.

#### Gene accessibility scores

Gene accessibility scores for a gene and metacell are defined as the fraction of gene-associated peaks that are open (Fig. 4b). Gene accessibility scores range from 0 (all correlated peaks closed) to 1 (all correlated peaks open).

$$\begin{array}{l}Gene\;Accessibility\left( {Gene\;g,\;Metacell\;m} \right) \\= \frac{{No.\;of\;open\;peaks\;in\;s\;correlated\;with\;expression\;of\;gene\;g}}{{No.\;of\;peaks\;correlated\;with\;expression\;of\;gene\;g}}\end{array}$$

### Multiome data generation

#### CD34^+^ bone marrow cells

Cryopreserved bone marrow stem/progenitor CD34^+^ cells from a healthy donor were purchased from AllCells (ABM022F) and stored in vapor phase nitrogen. The sample was immediately thawed at 37 °C in a water bath for 2 minutes with gentle shaking, and vial contents (1 ml) were transferred to a 50-ml conical tube. To prevent osmotic lysis and ensure gradual loss of cryoprotectant, 1 ml of warm medium (IMDM with 10% FBS supplement) was added dropwise after washing the storage vial while gently shaking the tube. Then, the cell suspension was serially diluted five times with 1:1 volume additions of warm complete growth medium with 2-minute wait between additions. The final ~32-ml volume of cell suspension was pelleted at 300*g* for 5 minutes. After removing the supernatant, cells were washed once with 10 ml of warm media and twice in ice-cold 1× PBS with 0.04% (w/v) BSA supplement to remove traces of medium. Cell concentration and viability were determined with a Countess II Automatic Cell Counter using the 0.4% trypan blue staining method.

Single Cell Multiome ATAC + Gene Expression was performed with a 10x Genomics system using Chromium Next GEM Single Cell Multiome Reagent Kit A (cat. no. 1000282) and ATAC Kit A (cat. no. 1000280) following the Chromium Next GEM Single Cell Multiome ATAC + Gene Expression Reagent Kit user guide and demonstrated protocol—Nuclei Isolation for Single Cell Multiome ATAC + Gene Expression Sequencing. In brief, 200,000 cells (viability 95%) were lysed for 4 minutes and resuspended in diluted nuclei buffer (10x Genomics, PN-2000207). Lysis efficiency and nuclei concentration were evaluated on a Countess II Automatic Cell Counter by trypan blue staining. In total, 9,660 nuclei were loaded per transposition reaction, targeting recovery of 6,000 nuclei after encapsulation. After transposition, reaction nuclei were encapsulated and barcoded. Next-generation sequencing libraries were constructed following the user guide, which were sequenced on an Illumina NovaSeq 6000 system.

#### T-cell-depleted bone marrow cells

Cryopreserved bone marrow cells from a healthy donor were purchased from AllCells (ABM007F) and stored in vapor phase nitrogen. The sample was immediately thawed at 37 °C in a water bath for 2 minutes with gentle shaking, and vial contents (1 ml) were transferred to a 50-ml conical tube. To prevent osmotic lysis and ensure gradual loss of cryoprotectant, 1 ml of warm medium (IMDM with 10% FBS supplement) was added dropwise after washing the storage vial while gently shaking the tube. Then, the cell suspension was dropwise diluted to 15 ml by the addition of warm complete growth medium. The final 15-ml volume of cell suspension was pelleted at room temperature, 400*g* for 5 minutes. After removing the supernatant, cells were washed once with 1 ml of Cell Staining Buffer (CSB) (BioLegend, 420201), centrifuged again at 400*g* for 5 minutes at 4 °C and resuspended in 100 µl of CSB. Concentration and viability were determined with a Countess II Automated Cell Counter using the 0.4% trypan blue staining method. Cells were incubated with Human TruStain FcX (Fc Receptor Blocking Solution) (BioLegend, 422301) for 10 minutes at 4 °C. After blocking, cells were stained with CD3 monoclonal antibody (UCHT1) (PE-Cyanine7, eBioscience, 25-0038-42) 1:100 for 20 minutes at 4 °C. Cells were washed twice with CSB before fluorescence-activated cell sorting (FACSymphony S6, BD Biosciences) where CD3^−^ cells were collected. Sorted cells were concentrated, and count and viability were determined with a Countess II Automated Cell Counter using trypan blue staining.

Single Cell Multiome ATAC + Gene Expression was performed with a 10x Genomics system as described above. In total, 300,000 cells (viability 95%) were used, and 16,100 nuclei were loaded per transposition reaction, targeting recovery of 10,000 nuclei after encapsulation.

We applied standard data processing procedures for both the newly generated data and the publicly available datasets. Further details are available in Supplementary Note 2.

### Application of SEACells

#### Metacell identification

SEACells was applied with default parameters to PBMC and CD34^+^ bone marrow datasets. The numbers of metacells were chosen as outlined in the ‘Note about number of metacells’ subsection, resulting in (1) 100 PBMC multiome, (2) 85 CD34 bone marrow multiome, (3) 100 T-cell-depleted bone marrow multiome and (4) 270 bone marrow mononuclear cell scATAC-seq metacells. SEACells was applied separately for the RNA and ATAC modalities of each multiome dataset using the PCA and SVD representations, respectively. Metacell raw counts for different datasets were determined as described in the ‘Metacell construction’ subsection. Metacell counts were normalized as described in the ‘Metacell annotation and normalization’ subsection.

#### Comparison of metacells from two modalities using PBMC multiome data

We used the paired nature of multiome data to determine how consistently metacells were identified between the two modalities. The clearly separated cell types in the PBMC multiome dataset were used for this analysis to verify whether relationships between metacells within and across cell types were consistent between the two data modalities. We checked whether single-cell groups derived using the ATAC modality could be applied to the RNA modality and retain cell type consistency.

We first computed the aggregated RNA metacell matrix and then a second aggregated gene expression using the single-cell groups from the ATAC modality instead of the RNA modality. We jointly normalized the two aggregated matrices, identified highly variable genes, computed PCs and visualized data using UMAPs (Supplementary Fig. 3a). No batch correction was used for this analysis. We repeated the same procedure using aggregated peak counts from ATAC and RNA metacells (Supplementary Fig. 3b).

#### Peak calling, gene scores and gene accessibility in the CD34^+^ bone marrow dataset

Peak calling, peak–gene associations, gene score computation and gene accessibility scores were determined as described in the ‘Toolkit for scATAC analysis’ subsection.

Because only scATAC is available for the bone marrow mononuclear cell dataset, peak–gene associations identified using the CD34 multiome dataset were used for the gene accessibility analysis.

### Robustness of SEACells algorithm

Owing to its more challenging continuous nature, we used the CD34 bone marrow data to assess the robustness of the SEACells algorithm.

#### Robustness to different initializations

Because the maximum–minimum sampling procedure relies on a random seed, we first tested the robustness of SEACells to different initializations. We compared the consistency of cluster labels across runs using the NMI score^18^, which is widely used to quantitatively evaluate the accuracy of clustering algorithms. We computed the NMI score (using the sklearn implementation sklearn.metrics.normalized_mutual_info_score) across five random initializations and found that the NMI score is generally 0.8 or higher (1 is best) across all datasets (Supplementary Fig. 6a).

#### Robustness to different numbers of metacells

The robustness to number of metacells was determined using the CD34^+^ RNA modality and NMI score, according to the same procedures outlined above (Supplementary Fig. 6b). We generally find strong reproducibility in SEACells assignment across varying numbers of SEACells.

#### Sensitivity of SEACells to detect rare cell types

To systematically assess the sensitivity of SEACells to capture rare cell states, we performed a downsampling experiment using the mouse gastrulation dataset from ref. ^19^. We subsampled different fractions of endothelial cells from the data while retaining all other cells and applied our SEACells algorithm to compute metacells. Specifically, we retained all endothelial cells (1,084, or 0.7% of total cells) or subsampled the endothelial cells such that they are 0.5% or 0.2% of total cells.

After the application of SEACells, we examined all metacells in which endothelial cells constituted at least 50% of the cells that define that metacell (Extended Data Fig. 2b). The recovery of the rare cell type is contingent on specifying the appropriate number of metacells to be recovered. To ensure that we detect rare populations at frequency 0.002, for example, we run SEACells with the parameter that each metacell contains, on average, 0.002 of the total cells in the population. Therefore, for the rarest population that contains approximately 230 cells, we search for at least 500 SEACells, or one metacell for every 230 cells.

#### Comparison of RNA metacells surface protein cell states

After application of SEACells, cell type purity was measured for each metacell using annotations from antibody-derived tag (ADT) data. Cell type purity is defined as the frequency of the most represented cell type in the metacell. SEACells metacell purities were compared to the metacells derived from the updated MetaCell-2 (ref. ^10^) algorithm for both coarse and fine resolution cell types using the Wilcoxon rank-sum test (Extended Data Fig. 6).

#### Comparison of different metacell approaches using benchmarking metrics

We developed several metrics to evaluate the quality of identified metacells and quantify the differences between alternative metacell approaches. Given that metacells represent distinct cell states of the biological system under consideration, inferred metacells should (1) be compact, meaning that they exhibit low variability among aggregated cells, and that most of this variability is a result of measurement noise, and (2) be well separated from neighboring metacells. Metrics that we developed to quantify these features are described in Supplementary Note 3.1.

We benchmarked SEACells against MetaCell^8^, MetaCell-2 (ref. ^10^) and SuperCell^69^, in addition to data imputation as an alternative approach to overcome data sparsity. For each dataset, MetaCell automatically infers the number of metacells and discards outliers. To compare faithfully across methods, we used the same number of partitions as input to SEACells and SuperCell on the same subset of data. MetaCell-2 also automatically determines the number of metacells, and we, therefore, used this number, which differed markedly from the number determined by MetaCell, to run a separate comparison to MetaCell-2. Details about the different metacell methods and their benchmarking are available in Supplementary Notes3.2 and 3.3.

Benchmarking metrics were computed for each metacell for all combinations of data modality, dataset and method. Cell type purity was used to assess the quality of PBMC metacells. Methods were compared using the Wilcoxon rank-sum test. We note that this test might possibly inflate significance due to the dependency between metacells, but it nonetheless provides an estimate of the direction of difference. Top-performing metacell approaches should have scores that are low on compactness, high on separation and high on cell type purity (Fig. 4c, Extended Data Fig. 7 and Supplementary Fig. 14).

We compared the metacell approaches using all metacells and separately for metacells in low-density and high-density regions to verify that all biologically relevant states are uniformly assessed. We once again used diffusion components to quantify the density of cells. Distance to the 150th neighbor in a single-cell nearest neighbor graph has been demonstrated to be a reasonable approximation for the density in the high-dimensional space^7^. We computed the distance to the 150th neighbor for each single cell using diffusion components. Single cells with densities in the upper quartile of distances were designated as ‘low-density cells’, and, similarly, those in the lower quartile of distances were designated as ‘high-density cells’. Analogously, metacells containing these low-density cells were designated as low-density metacells and vice versa for high-density metacells. The proportion of all metacells designated as either low density or high density were each capped at 30% of all metacells, and these were used as low-density and high-density regions, respectively, for comparisons (Extended Data Fig. 7).

### Transcriptional regulators of hematopoietic differentiation

#### Application of Palantir on CD34^+^ bone marrow data

Palantir^4^ with default parameters was applied to the RNA modality of CD34 bone marrow multiome data at single-cell level, with the number of informative DCs (*n* = 7) identified using the eigengap statistic. A CD34-high hematopoietic stem cell was selected as the start cell, and terminal states for erythroid, lymphoid, megakaryocyte, monocyte, conventional dendritic cell (cDC) and pDC lineages were all set manually. The pseudotime ordering of metacells was computed as the average pseudotime ordering of the constituent single cells. The metacells annotated as HSC, MEP and erythroid in the CD34^+^ bone marrow dataset were used for TF activity inference.

#### Gene expression trends

We used scanpy to identify the sets of genes that were differentially expressed among cell types in HSC, MEP or erythroid (adjusted *P* < 1 × 10^−2^, fold change > 1.5), using the union of gene sets from these cell types for TF activity inference (Extended Data Fig. 4a).

For each gene, expression trends were determined using generalized additive models (GAMs)^70^. A GAM was fit for gene accessibility trend as a function of the Palantir pseudotime for each gene. Expression of *g* in metacell *i*, *y*_*gi*_, is fit as

$$y_{gi} = \beta _o + f\left( {\tau _i} \right),$$

where *i* is a metacell along the relevant lineage, and *τ*_*i*_ is the Palantir pseudotime ordering of metacell *i*. Cubic splines are used as the smoothing functions because they are effective in capturing non-linear relationships. The fitted expression for each metacell is *z*-scored and used as input for TF activity inference.

#### TF activity inference

The TF–target matrix was constructed using the subset of peaks that are significantly correlated with the set of genes under consideration using metacells (Extended Data Fig. 4b). The TF–target matrix using single-cell data was too sparse to provide meaningful inputs (Extended Data Fig. 4c). Lasso regression was performed on a metacell-by-metacell basis to infer the TF activities (Extended Data Fig. 4d). Total activities across all erythroid metacells were used to rank the TFs.

The results of TF activity inference based on metacells was compared to the results based on the TF–target matrix using cell-type-specific peaks. DESeq2 (ref. ^71^) was used to identify cell-type-specific peaks (adjusted *P* < 0.01, fold change > 1.5) by comparing metacells of one cell type with all other metacells. We used the procedure in the ‘Inference of transcription factor activity using metacells’ subsection to construct a cell-type-specific TF–target matrix, which was used to predict gene expression per metacell (Extended Data Fig. 4e).

#### Application to T-cell-depleted bone marrow data

The procedure used for the CD34 bone marrow dataset was also used to identify TF activities in the T-cell-depleted bone marrow dataset by selecting the subset of metacells belonging to erythroid, B cell and monocyte lineages (Supplementary Fig. 10). The characterization of hematopoietic dynamics is described in Supplementary Note 4.

### SEACells application to COVID-19 samples and data integration

#### Comparison of healthy individuals and patients with critical COVID-19

##### SEACells

SEACells metacells were computed separately for each sample using approximately one metacell for every 75 single cells, following the procedure described in the ‘Note about number of metacells’ subsection. After metacell identification, an aggregated metacell-by-gene expression matrix was computed for each sample.

##### Mapping of SEACells metacells between individuals

We mapped metacells across patients to determine consistency. For each pair of patients, Harmony^45^-corrected metacell PCs were used to compute the top ten DCs, which were used for downstream analysis. For each metacell in a patient, nearest metacell neighbors from the second patient were computed. Two metacells from different patients were considered equivalent if they were mutually in each other’s top two nearest neighbors (Supplementary Fig. 18a,b). We quantified the comparison for each pair of samples by computing the proportion of mapped metacells with matching cell types (Supplementary Fig. 18c).

##### Batch correction with metacells

Combining metacells across all samples highlighted the batch effects (Extended Data Fig. 9b). Harmony^45^ was used to perform batch correction of metacells across the 40 samples using metacell-aggregated gene expression matrices with default parameters. Harmony (scanpy.external.pp.harmony_integrate) was applied to the PCs derived from the top 1,500 highly variable genes using the sample as the batch covariate.

##### GSEA to characterize differences between collection sites

After batch correction of metacells, we observed that CD4^+^ T cells continued to be separated by collection site. We performed differential expression between CD4^+^ T metacells collected at different sites and applied gene set analysis^72^ using curated T-cell-relevant gene sets to explore whether the observed differences reflect differences in underlying biology (Supplementary Table 1). The normalized enrichment scores were calculated for each T-cell-relevant pathway across collection sites (Extended Data Fig. 9d). In particular, samples from Cambridge are significantly enriched (*P* < 0.05) for genes implicated in SASP signaling (top genes include *Il1B*, *CCL2* and *IIFNG*) and TCR activation pathway (top genes include *GADD45G* and *EGR1*); samples from the Sanger site are uniquely enriched (*P* < 0.05) for hypoxia response (top genes include *SIAH2* and *PNRC1*), NOD-like receptor signaling (top genes include *NFKB1*, *MAPK14* and *CHUK*) and glutathione metabolism (top genes include *HAGH*, *GGT1* and *PRDX1*); and samples from the Newcastle site are enriched (*P* < 0.05) for genes implicated in JAK-STAT signaling (top genes include *IFNAR2*, *IL2RG*, *IRF9*, *JAK3* and *STAT3*), mitophagy (top genes include *CDC37* and *SQSTM1*) and oxidative phosphorylation (top genes include *NDUFB8*, *ATP5PO*, *FXN* and *NDUFA7*), among others (Extended Data Fig. 9d). These pathways are critical determinants of T cell state and demonstrate that samples across batches encode biological differences.

##### Single-cell batch correction

Single-cell batch correction was performed using Harmony^45^ with default parameters for the analysis in Extended Data Fig. 9 and Extended Data Fig. 10d–f. In total, 1,500 highly variable genes were used for all the analyses, and the sample was used as the batch covariate.

##### Differential abundance testing of cell states between healthy individuals and patients with COVID-19

By aggregating single cells that most likely differ due to technical noise, metacells provide a robust segmentation of the data. Metacells are, thus, more robust entities compared to single cells and provide a concrete baseline to infer altered cell state abundances across conditions (Extended Data Fig. 9).

##### Generation of aggregated meta^2^cells in COVID data

Although mapping metacells demonstrates consistency between pairs of individuals, the approach does not provide a path to identify *similarities* and *differences* between healthy individuals and patients with COVID-19. We, therefore, devised a procedure for comparison across any number of patients to identify enriched and depleted metacells in different conditions (Extended Data Fig. 10a).

We re-computed SEACells metacells using the aggregated and batch-corrected metacell count matrices for each sample. These second-level metacells, or meta^2^cells, therefore contain metacells across healthy and critical patient samples. To compute meta^2^cells, we ran the algorithm asking for approximately one meta^2^cell for every ten metacells, because the dataset was already highly summarized in the first round of aggregation.

To summarize the cell type annotations of cells in a constituent meta^2^cell, the modal cell type of constituent cells was chosen if the purity was greater than 80%; otherwise, the cell type was denoted as ‘Mixed’.

##### Differential abundance of cell states in patients with COVID-19

The meta^2^cells computed across healthy individuals and patients with critical disease define cell states, each of which may be more strongly associated with health or disease. We computed the proportion of COVID-19 metacells in each meta^2^cell, providing a measure of differential abundance of cell state in patients with COVID-19. We then devised a permutation test to assess the significance of these differential abundances.

First, the metacell-to-meta^2^cell assignments were randomly permuted. The number of metacells assigned to each meta^2^cell did not change, but the constituent metacells and their associated healthy/COVID-19 labels were permuted, providing a representative background distribution. Next, the proportion of metacells derived from COVID-19 samples assigned to each meta^2^cell was computed. This procedure was repeated for 5,000 permutation trials, and a null distribution on COVID-19-enriched metacell proportions was derived for each meta^2^cell. The null distribution was then used to compute a *P* value, and a significant enrichment threshold for cell states in COVID-19 was set at *P* < 0.1.

##### Gene signatures of enriched cell states

To assess the biological distinctions between healthy and diseased meta^2^cell states, we identified the differentially expressed genes for each meta^2^cell by comparing against other meta^2^cells of the same cell type using scanpy.tl.rank_genes_groups.

##### Comparison to single-cell data integration approaches

To compare whether the correspondence between T cell phenotypes and temporal stages of disease can be recovered using single-cell data integration, we applied Harmony^45^ and scVI^46^ batch correction at the single-cell level using sample as the batch covariate (Extended Data Fig. 10d,e). We next computed UMAPs using the batch-corrected PCA space (latent space for scVI) and highlighted the cells that constitute the three aggregated SEACells (Fig. 6) that show correspondence between T cell activation phenotypes and temporal stages of disease (Extended Data Fig. 10d,e). ‘Pseudo-metacells’ were defined using batch-corrected single-cell data to enable comparison against the metacells highlighted in Fig. 6d. We first used the Harmony-corrected PCA space (or scVI latent space) to identify the median cell among the single cells that constitute each metacell and then computed the median cellʼs neighborhood (containing 1,078, 1,161 or 1,119 cells, corresponding to metacells A, B and C, respectively) and aggregated cells within the neighborhood to define pseudo-metacells. Aggregated expression of these pseudo-metacells were compared against the meta^2^cell gene expression patterns (Extended Data Fig. 10f).

##### Comparison to single-cell differential abundance testing

We employed the extensively used Milo^55^ algorithm to perform differential abundance testing at the single-cell level and compared the results to differential abundance testing using metacells. MiloR typically accepts a SingleCellExperiment object as input. However, owing to memory constraints in passing raw counts for all 177,242 cells, we provided MiloR with the pre-computed batch-corrected PCs, annotated with the sample of origin and sample condition. Default parameters as specified in the Milo vignettes were then used to compute neighborhoods as well as their differential abundances. All neighborhoods with at least 80% CD4^+^ cell type purity were selected for downstream analysis, yielding 276 neighborhoods.

Gene signatures identified in the SEACells metacells of interest were used to compute a gene signature score for each MiloR neighborhood. The gene signature score was computed for each cell by summing across the expression *z*-scores of the signature genes. Gene signature scores at the neighborhood level were computed by averaging the scores of single cells that constitute the neighborhood. To assess whether the cell states highlighted in Fig. 6d could be identified using differential abundance testing at single-cell level, we compared the Milo neighborhood gene signature scores with the gene scores derived using SEACells meta^2^cell (Extended Data Fig. 10g,h).

##### SEACells application to the full COVID-19 atlas

As with the healthy and critical COVID-19 sample analyses, SEACells was applied to each sample separately by requesting a metacell for every 75 cells, resulting in a total of 8,092 metacells. Harmony batch correction was applied at the metacell level using the site as the batch covariate. We wanted to evaluate cell type purity and the mixing of cell types in each metacell neighborhood to assess the effectiveness of batch correction. For cell type purity, we employed the same steps as described in the ‘Metrics for metacell benchmarking’ subsection. For cell type mixing, we computed the distribution of different cell types within each of the metacell neighborhoods (defined as the ten nearest neighbors on the Harmony-corrected PCA space) to compute Shannon’s entropy for each cell, similar to the technique used in ref. ^73^. The higher the entropy, the more mixed the neighborhood of the metacell is, indicating that cell types are not grouped together after batch correction.

### Reporting summary

Further information on research design is available in the Nature Portfolio Reporting Summary linked to this article.

## Online content

Any methods, additional references, Nature Portfolio reporting summaries, source data, extended data, supplementary information, acknowledgements, peer review information; details of author contributions and competing interests; and statements of data and code availability are available at 10.1038/s41587-023-01716-9.

### Supplementary information

Supplementary Information Supplementary Figs. 1–20 and Supplementary Notes 1–4

Reporting Summary

Supplementary Table T cell gene signatures

## Data Availability

The CD34 bone marrow and T-cell-depleted bone marrow multiome datasets are available on the Gene Expression Omnibus (https://www.ncbi.nlm.nih.gov/geo/query/acc.cgi?acc=GSE200046). Filtered and processed count matrices, including cell type annotations and ATAC fragment files, are available on *Zenodo* at 10.5281/zenodo.6383269 (ref. ^74^). The following publicly available datasets were used in the manuscript: 10x PBMC Multiome^17^, 10x PBMC CITE-seq^37^, scRNA-seq of lung adenocarcinoma (https://www.ncbi.nlm.nih.gov/geo/query/acc.cgi?acc=GSE123904)^36^ and mouse gastrulation atlas (https://www.ebi.ac.uk/biostudies/arrayexpress/studies/E-MTAB-6967)^19^.

## Code availability

SEACells is available as a Python module at https://github.com/dpeerlab/SEACells (ref. ^75^). Jupyter notebooks detailing the use of SEACells, including metacell identification, aggregation and the ATAC pre-processing, and the gene regulatory toolkit are available at https://github.com/dpeerlab/SEACells/tree/main/notebooks. Jupyter notebooks to reproduce figures in the manuscript are available at https://github.com/dpeerlab/SEACellsReproducibility. Modified ArchR pipeline for peak calling using NFR fragments is available at https://github.com/dpeerlab/ArchR.
