# Supplementary material for: SEACells infers transcriptional and epigenomic cellular states from single-cell genomics data
Source: Nat Biotechnol. 2023 Mar 27;41(12):1746–57. doi: 10.1038/s41587-023-01716-9 (PMC10713451; doi:10.1038/s41587-023-01716-9)
Supplement: Supplementary file 1 — Supplementary Figs. 1–20 and Supplementary Notes 1–4 [file 41587_2023_1716_MOESM1_ESM.pdf]

nature biotechnology

Article https://doi.org/10.1038/s41587-023-01716-9

SEACells infers transcriptional and epigenomic cellular states from single-cell genomics data

In the format provided by the authors and unedited

Supplementary Note 1: SEACells algorithm

Supplementary Note 1.1: Why kernel archetype analysis best is suited for metacells

Traditionally, archetypal optimization has been applied to reconstruct the original data matrix, X, in order to study the geometry of cell distribution and to *characterize biology at the boundaries of the cellular phenotypic space*[1-3]. In such settings, the phenotypic space is described using a linear convex hull approximation technique that does not necessarily consider any non-linear structure *within* the phenotypic space. As such, the "internal" regions of the cell phenotype space are completely ignored (**Extended Fig. 1B**, highlighted region).

Archetype analysis in this kernel space is well suited to identify metacells. Kernel space is a Cartesian space, where the "phenotype" of a cell is defined by its similarity—quantified in the kernel matrix *(M)*—to every other cell. By definition, similarity scores are between 0 and 1; thus, all cells are in the positive quadrant, encapsulated by a hypersphere of radius strictly less than sqrt(*N*), where *N* is the number of cells. Intuitively, the kernel trick is casting highly similar cells into tiny clusters along a cone emanating from the origin. Within each small group of highly similar cells, the most representative cell of the group has to be most similar to every other cell in that group. In other words, the best representative of the unique cellular state of the highly similar cell group is the one that is most connected to every other cell in the group. We claim that such a best candidate is likely to exist at the extreme (corner) in the kernel space.

Consider the blue cells in the toy example in **Extended Fig. 1C**. The cell *(i)* that is furthest from the origin (i.e., the extreme) will have the highest L2-norm defined as $\sum_{j=1}^{N} M(i,j)^2$. This suggests that for a set of highly similar cells in this kernel space, which by construction lie close to each other, the most connected cell is also the most extreme cell. The most connected cell is also the best candidate for a metacell. Therefore, to identify metacells in this kernel space, we need to look for cells at the extreme, which led us to using archetype analysis.

Furthermore, the construction of the kernel utilizes the k-nearest neighbor graph to "unfold" any non-linear structure in the phenotypic space, making the interior regions of the manifold accessible for characterization (**Extended Fig. 1D**). As a result, the number of archetypes used in SEACells is substantially greater than what is used in typical archetypal analysis. The greater number of archetypes in addition to the strict definition of cell-cell similarity using adaptive Gaussian kernels creates a large number of pockets of highly similar cells, each representing a unique biological state (**Extended Fig. 1E**), making kernel archetypal analysis ideally suited to identify metacells representing biological states.

Supplementary Note 1.2: Optimization convergence

Archetypal analysis is an iterative procedure, whose termination depends on a pre-defined convergence criteria on the reconstruction error. The reconstruction error, R, is defined as the norm of the difference between the true kernel matrix, M, and the reconstructed kernel matrix, $\bar{M} = MBA$. Formally, for iteration i, the reconstruction error is

$$R^{(i)} = || M - MB^{(i)}A^{(i)}||^2 ,$$

where $A^{(i)}$ and $B^{(i)}$ are the cell assignment and archetype weight matrices on the ith iteration respectively.

A user-specified convergence percentage, typically on the order of 1e-5, is supplied to the algorithm, and used to calculate the convergence threshold, t, as the product between the user-specified convergence percentage and the initial reconstruction error, $R^{(0)}$. SEACell converges when the difference between the reconstruction error on successive iterations falls below the computed convergence threshold. Formally, SEACells converges when

$$||R^{(i)} - R^{(i-1)}|| < t .$$

Supplementary Note 2: Data Processing

CD34⁺ bone marrow multiome data - RNA modality

Count matrices for the two samples were generated using CellRanger ARC[4]. A total of 13946 barcodes were identified as cell containing barcodes by CellRanger ARC. Starting with the filtered barcode matrices from CellRanger ARC, barcodes from the bottom and top 2.5th percentile in molecule counts were excluded. Further cells with less than 0.4 fraction of reads in peaks in ATAC modality (3% of total) and greater than 25% of reads from mitochondria from the RNA modality were excluded from downstream analysis (2.7% total). The specified cutoffs were also chosen based on the respective empirical distributions to remove outliers.

Data was generated in two lanes. For each sample, scrublet[5] with default parameters was used to compute doublet scores, and a cluster of cells with high doublet scores was removed from downstream analysis (5% total; note that $CD34^+$ has a continuous nature that can lead to false doublet calls). Following the filtering steps, the two samples were concatenated and normalized for molecule counts by dividing raw counts by the total counts per cell. The normalized data was multiplied by the median of total counts across cells to avoid numerical issues and log-transformed with a pseudocount of 0.1. Feature selection was then performed to select the top 2500 most highly variable genes (using `scanpy.pp.highly_variable_genes`), which was used as input for principal component analysis with 50 components. The parameters were chosen based on prior analysis of a $CD34^+$ scRNA-seq dataset[6].

The PCs were used as input for generating UMAPs and clustering with PhenoGraph[7]. The preprocessing and analysis were undertaken using scanpy[8] Diffusion components were generated using the adaptive kernel following the functions in the Palantir package[6] and imputation of gene expression was performed using MAGIC[9]. Each cluster was annotated with specific cell types using the markers defined in ref.[6], and mature B cells were excluded from the analysis (35% of total). Highly variable gene selection, PCA, clustering, visualizations, diffusion maps and imputation were repeated following B-cell exclusion. A total of 6881 cells were retained after all the filtering steps. Note that imputation was only used to compute single-cell gene expression trends in **Supplementary Fig. 4**; it was not used for metacell computation or other downstream analyses.

$CD34^+$ bone marrow multiome data - ATAC modality

To analyze the ATAC modality, we used the ArchR pipeline[10] on the cell subset that remained after filtering in the RNA modality, employing 100k instead of the default 25k features. In ArchR, data was normalized using IterativeLSI and SVD to determine a lower-dimensional representation of the sparse data. The first SVD component showed greater than 0.97 correlation with log library size and was excluded from downstream analysis. SVD was used as input to cluster the data with PhenoGraph and visualized using UMAPs. The modified ArchR pipeline (described in Methods, “Peak calling”) was used for peak calling.

T-cell depleted multiome data

The preprocessing and analysis of RNA and ATAC modalities was performed following the steps outlined for the $CD34^+$ bone marrow data. NK cells, mature monocytes and B cells were excluded from the analysis in **Supplementary Fig. 7**. A total of 7439 cells were retained after all the filtering steps.

PBMC multiome data

Counts for the PBMC multiome data were downloaded from 10x Genomics. The preprocessing and analysis of RNA and ATAC modalities was performed following the steps outlined for the $CD34^+$ bone marrow data. Cell-type annotation was performed using the marker genes in ref.[11] and no cell types were excluded from the analysis. A total of 11543 cells were retained after all the filtering steps.

Lung adenocarcinoma

Fully annotated count matrices for single-cell profiling of lung adenocarcinoma in patient samples were downloaded from ref[12]. All non-immune cells contained in the data were used in the analyses, comprising a total of 4770 cells. Each patient sample was individually processed by performing normalization on raw counts, followed by log transformation. Following the procedure outlined in the manuscript, the 1500 most highly variable genes were identified, and principal components were computed from the expression of these genes.

Bone marrow mononuclear cells scATAC-seq dataset

Fragment files for single-cell ATAC-seq data of bone marrow mononuclear cells and $CD34^+$ cells (total of 5 samples) and the respective cell type annotations were downloaded from GEO[26]. All the cells described in the manuscript[13] were used except for T cells, since they do not differentiate in the bone marrow. The preprocessing and peak calling followed the same procedure outlined for the ATAC modality of the $CD34^+$ bone marrow multiome dataset. A total of 19438 cells were retained after all the filtering steps (**Supplementary Fig. 7**).

Mouse gastrulation atlas

Mouse gastrulation data was downloaded as scanpy anndata objects from ref.[14] and was already normalized, batch corrected and included principal components across all cells. This dataset contains approximately 116,000 single cells across a range of cell, including endothelial cells among the cell types with fewest counts (**Extended Fig. 2A**). The pre-computed principal components were used as input for SEACells.

PBMC CITE-seq data

10x PBMC CITE-seq dataset was downloaded from refs.[15,16]. This data contained manually curated coarse and fine-grained cell type annotations based on ADT data and was used to verify whether SEACells metacells identified using the RNA modality are consistent with the cell types based on ADT data. The dataset contains 8201 cells. SEACells was applied using the heuristic of one metacell for every 75 cells, for a total of 109 metacells, to recover metacells using the RNA modality (**Extended Fig. 18B,C**).

Supplementary Note 3: Metacells methods comparison

An ideal metacell is compact (it exhibits low variance amongst constituent cells), well-separated (it remains distant from cells of a neighboring metacell) and homogenous (it has high cell-type purity). Below, we develop metrics to measure these properties, introduce currently available algorithms for metacell specification, and benchmark methods on $CD34^+$ bone marrow and PMBC data from both RNA and ATAC modalities. We also compare SEACells with imputation for the purposes of finding gene-accessibility peak correlations.

Supplementary Note 3.1: Metrics for meta-cell benchmarking and comparison

We used diffusion components (DCs)[6,17] to quantify the compactness and separation of metacells. Each DC represents a key axis of biological variance in both continuous trajectories and discrete states and thus provides an ideal platform to quantify metacell qualities. We first compute DCs using single-cell data. For scRNA-seq, DCs are computed based on principal components, and for scATAC-seq, based on the singular value decomposition following preprocessing of single-cell data (described in **Supplementary Note 2**). The number of components can be chosen by the eigengap statistic. We noted that across datasets and modalities, the number of DCs ranged from 6–8. For consistency and simplicity, we fixed the number of DCs as 10 for all evaluations.

Compactness

Compactness provides a measure of how cell homogeneity within a metacell.

For each metacell, the variance in each DC dimension is computed across the cells that constitute that metacell. The average variance across components is reported as the compactness. Since DCs are orthonormal, we can compute the variance of each component separately. The average variance ensures that the homogeneity of cells that constitute the metacell are measured across all axes of biological variance.

For a metacell, s, the compactness, $Compactness(s)$ is formally defined as follows.

$$Compactness(S) \; = \frac{1}{d}\sum_{i=1}^{d} variance_{cells \in S}\,(DC_i)$$

where $DC \in R^{n \times d}$ where is the matrix of diffusion components computed using single-cell data.

A high quality metacell should have a low compactness score indicating low variability or equivalently high homogeneity amongst the cells that constitute the metacell.

Separation

To assess whether metacells are distinct from each other, we evaluated the separation between neighboring metacells using diffusion components. For each metacell, diffusion embedding is determined as the average of the cells that constitute the metacell. Euclidean distance in the diffusion embedding between the metacell and its nearest neighbor is reported as the separation of the metacell. A greater distance between metacells determined in diffusion space indicates a better separation between them.

Cell-type purity

Cell-type purity is a measure of the consistency of cell types amongst cells that constitute a metacell, and was introduced to assess the quality of SuperCell[18]. Cell-type purity is computed as the proportion of cells which belong to the modal cell type in a metacell. Note that purity metric is applicable and valid when the biological system under consideration comprises distinct cell types with distinct functions, such as PBMCs. Cell-type purity is not a reliable metric for continuous trajectories since the different compartments are merely a partitioning of the trajectory and do not necessarily represent well-separated cell types.

Supplementary Note 3.2: Alternative methods and their application

MetaCell (Baran et al.)

The MetaCell[19] approach uses a non-parametric graph algorithm to partition scRNA-seq data into distinct metacells. This algorithm constructs a balanced kNN graph, which is subsampled multiple times into dense subgraphs in order to determine metacell partitions. Outlier cells are identified, and the final output is the assignment of cells to metacells. MetaCell was run using the default processing steps outlined in https://tanaylab.github.io/metacell/articles/a-basic_pbmc8k.html with raw count data.

We ran MetaCell with default parameters on three scRNA-seq datasets—$CD34^+$ bone marrow, PBMC and lung adenocarcinoma, to evaluate performance in the contexts of continuous differentiation, discrete cell states and cancer, respectively. For each dataset, MetaCell automatically infers the number of metacells and discards a subset of the data as outliers. To compare faithfully across methods, we used the same number of partitions as input to SEACells and SuperCell on the same subset of data.

To apply MetaCell to scATAC-seq data, the peak count matrices were modified by mapping peaks to the nearest gene and aggregating all peaks within each gene to create a pseudo cell-by-gene count matrix as input. Following this representation, we ran MetaCell on the $CD34^+$ bone marrow and PBMC scATAC-seq datasets with default parameters.

SuperCell (Bilous et al.)

SuperCell[18] uses the walktrap algorithm, a community detection approach to partition nodes in a single-cell graph into a predefined number of metacells using random walks. The walktrap algorithm constructs a single-cell graph, placing edges between cells with similar transcriptomic profiles, and merges nodes which are highly connected. In essence, SuperCell is thus a community detection algorithm with higher resolution, and performs better than typically used algorithms such as louvain for metacell identification.

The number of metacells is a parameter of the SuperCell algorithm, similar to SEACells. SuperCell was run using the default parameters specified in https://github.com/GfellerLab/SuperCell, with the graining level chosen to obtain the same number of partitions as those obtained by Baran et al. MetaCell, in order to compare methods across similar levels of granularity. We ran SuperCell on the $CD34^+$ bone marrow and PBMC scRNA-seq datasets using default parameters.

Since SuperCell requires a gene expression matrix as input, we applied it to $CD34^+$ bone marrow and PBMC scATAC-seq data using the same aggregation approach we used for running MetaCell.

Metacell-2 (Ben-kiki et al.)

Metacell-2 is an update of the MetaCell algorithm, that uses a recursive divide-and-conquer approach to scale the identification of metacells to atlas-scale data[20]. We ran the Metacell-2 algorithm with the default parameters specified at https://github.com/tanaylab/metacells.

Supplementary Note 3.3: Benchmarking and comparison

The number of metacells in MetaCell and Metacell-2 are automatically determined and cannot be controlled by a user-defined parameter. In our core benchmarking dataset, we observed that Metacell-2 led to a substantially higher number of metacells in each dataset.

Dataset	*No. metacells – MetaCell (Baran et al.)*	*No. metacells – Metacell-2 (Ben-kiki et al.)*
PBMC ATAC	98	408
PBMC RNA	98	424
CD34 ATAC	86	285
CD34 RNA	65	297

Therefore, to ensure a fair comparison of methods, we ran separate benchmarks using the number of metacells identified by each of these two methods. In both bone marrow and peripheral blood ATAC datasets, we found that SEACells metacells are substantially more compact and better separated than MetaCell and SuperCell metacells (**Fig. 4C**, **Extended Fig. 7A,B**), especially in low cell-density regions. SuperCell does show marginally better separation in high density regions, since it creates a few very large partitions containing hundreds of cells in these regions (**Supplementary Fig. 11C**).

While the different approaches are qualitatively similar using the RNA modality (**Supplementary Fig. 12B,C**), SEACells metacells are significantly more compact than SuperCell and Metacell-2 ($P < 1e\text{-}5$, Wilcoxon rank-sum test) and marginally more compact than MetaCell (**Extended Fig. 7C**). Conversely, SEACells RNA metacells are significantly better separated than MetaCell and Metacell-2 metacells ($P < 1e\text{-}2$, Wilcoxon rank-sum test), whereas the separation of SuperCell is artificially boosted by the large partition size in high density regions (**Extended Fig. 7D**). Similar to ATAC, SEACells metacells have greater cell-type purity in the PBMC RNA data, which comprises distinct cell types (**Extended Fig. 6A**).

Comparison to imputation

scVI[21] and peakVI[22] were used for imputation of RNA and ATAC-seq data, respectively, to determine their ability to identify strong gene-peak correlations in the $CD34^+$ bone marrow dataset. By default, peakVI requires a peak to be accessible in at least 5% of cells, which led to the filtering of 85% of peaks in the data (~35k peaks out ~240k were retained). We determined gene-peak associations using the same procedure we employed for SEACells. Specifically, peaks within +/-100kb of the gene were identified, and the correlations between peak accessibility and gene expression were computed using imputed single-cell data. Since a measure of significance of correlation is unavailable, peaks with correlation > 0.1 were nominated as candidate regulatory regions. ~20k out of the ~35k peaks were identified as candidate regulatory regions. By comparison, SEACells analysis identified ~75k peaks of the ~240k peaks as significantly correlated. Imputed accessibility of correlated peaks was aggregated to derive gene scores and compared with imputed gene expression (**Extended Fig. 3D**).

Supplementary Note 4: Characterization of hematopoietic dynamics

Accessibility distributions for metacells in **Fig. 5D** were computed separately for each metacell using the fraction of open peaks in highly variable genes. For the single-cell pseudotime bins in **Fig. 5D**, cells were categorized into one of forty bins based on their Palantir pseudotime order, which ranged from 0.0–0.82 for the erythroid lineage. We then created 40 equal sized bins with a bin size of 0.02 and assigned each cell to the respective bin. Fragments that belong to all cells in a bin were pooled and open peaks identified using the Poisson procedure.

Accessibility trends

Gene accessibility trends were determined using generalized additive models (GAMs)[23]. A GAM was fit for gene accessibility trend as a function of the Palantir pseudotime for each gene. Gene accessibility of g in cell i, y_{gi} is fit as

$$y_{gi} = \beta_o + f(\tau_i) \ ,$$

where i is a cell along the relevant lineage, τ_i is the Palantir pseudo-temporal ordering of cell i. Cubic splines were used as the smoothing functions, since they are effective at capturing non-linear relationships. The pseudotime was then divided into 150 equally sized bins, and the smooth trend was derived by using the fit from the GAM to predict the accessibility of the gene at each bin.

Gene ontology analysis

Gene ontology analysis (**Fig. 5E**) was performed to identify enriched ontologies in genes with increasing or decreasing accessibility, measuring enrichment using the hypergeometric test. The “c7: immunologic signature” gene sets from Molecular Signature Database (MSigDB) were used (http://software.broadinstitute.org/gsea/msigdb/index.jsp).

Motif enrichments in genes with changing accessibility

Predicted TF binding sites in each peak were determined using FIMO[24] using default parameters and the cisBP v2 motif database[25]. Hypergeometric tests were used to identify the most enriched motifs in peaks with increasing or decreasing accessibility using all the peaks as the background.

Supplementary Figures

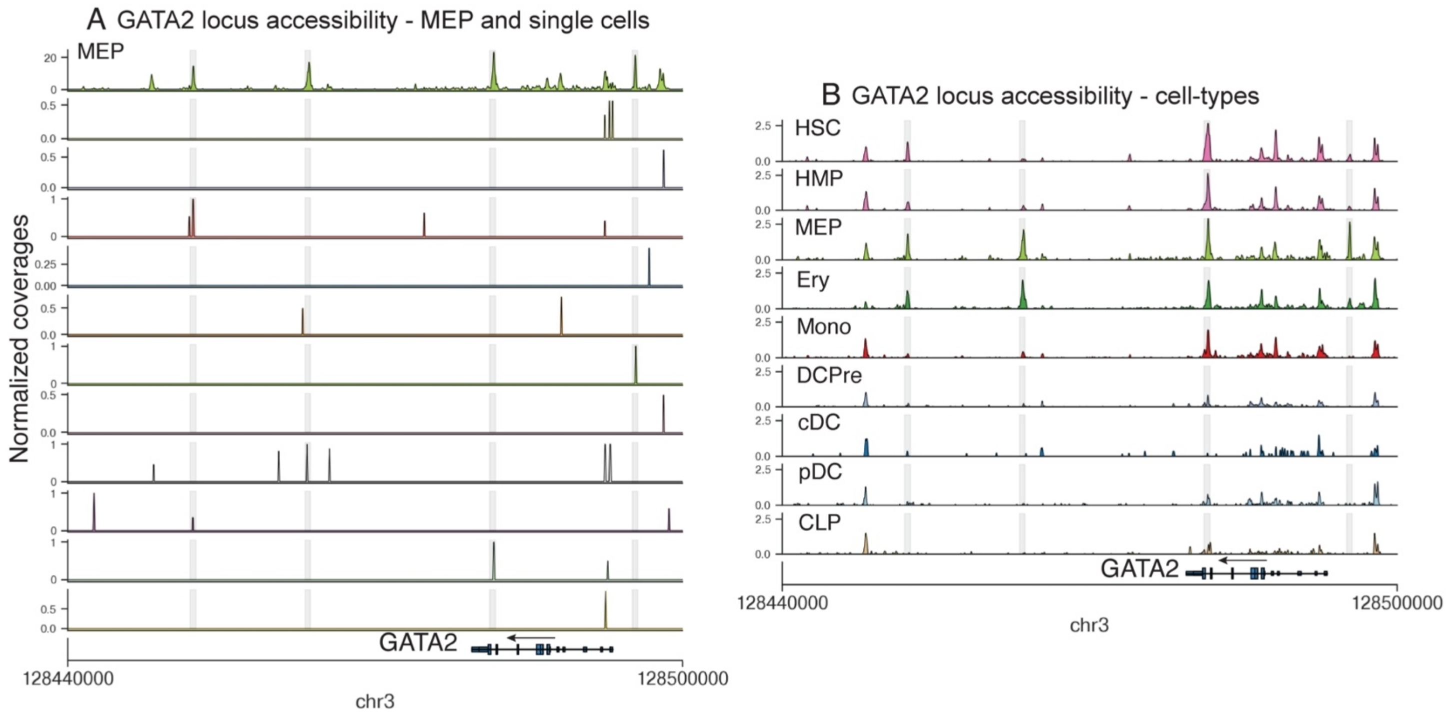

Supplementary Fig. 1: *GATA2* locus accessibility.

A. *GATA2* locus accessibility profiles of a random sample of single MEP cells, highlighting the noise and sparsity of single-cell ATAC-seq data. Top row represents an aggregate of 384 MEP cells; each other row represents an individual cell.

B. Accessibility landscape of the *GATA2* locus, across all hematopoietic cell types.

Highlighted peaks correlate significantly with *GATA2* expression (Two-sided nominal $P < 0.1$, Empirical null distribution).

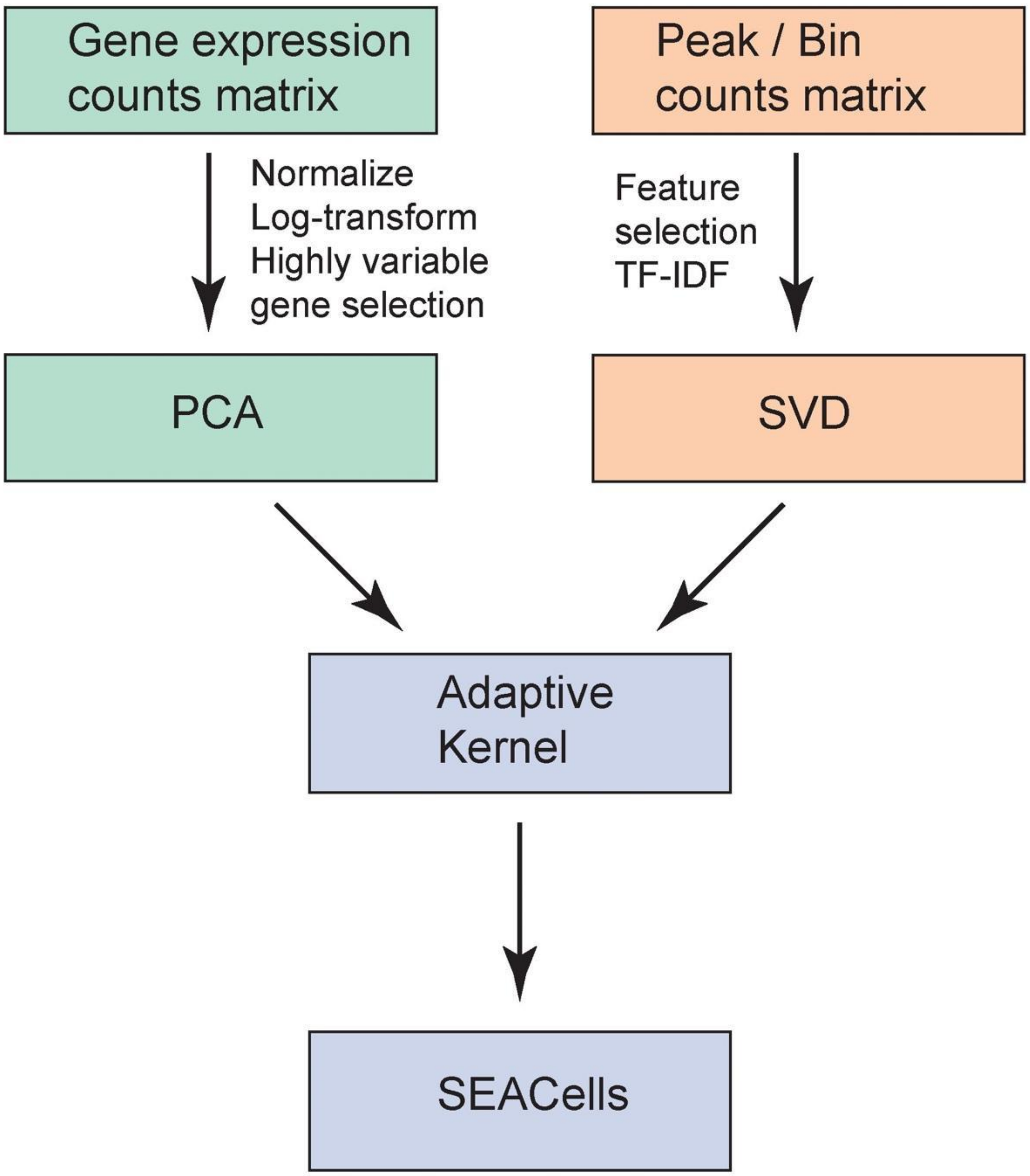

Supplementary Fig. 2: SEACells workflow for scRNA-seq and scATAC-seq data.

scRNA-seq and scATAC-seq count matrices are preprocessed differently, using procedures appropriate for the noise and biases of each data type. Principal component analysis (PCA) and singular value decomposition (SVD) are then used to generate a lower-dimensional representation for RNA and ATAC data, respectively. Next, a density adaptive kernel is constructed using the reduced dimensional representations, and input to the SEACells algorithm for metacell construction.

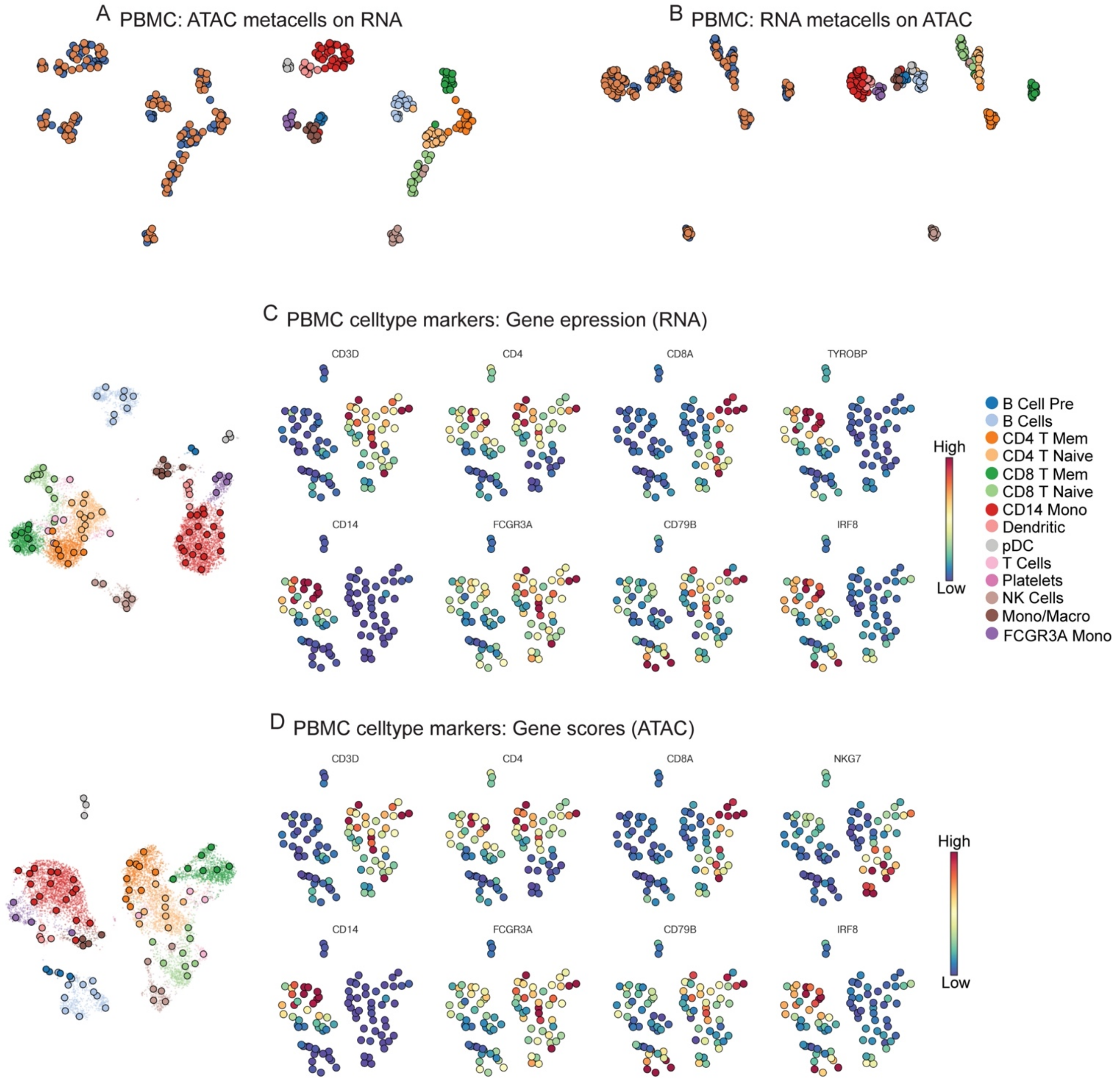

Supplementary Fig. 3: Metacells are consistent across data modalities and enable cell type identification in PBMC data.

A,B. UMAPs based on combined ATAC and RNA metacells derived from the PBMC multiome dataset. ATAC metacells were projected on RNA metacells (A) or RNA metacells were projected on ATAC metacells (B) (Methods).

C. Expression of key PBMC cell type markers per RNA metacell.

D. Gene scores of key PBMC cell type markers per ATAC metacell

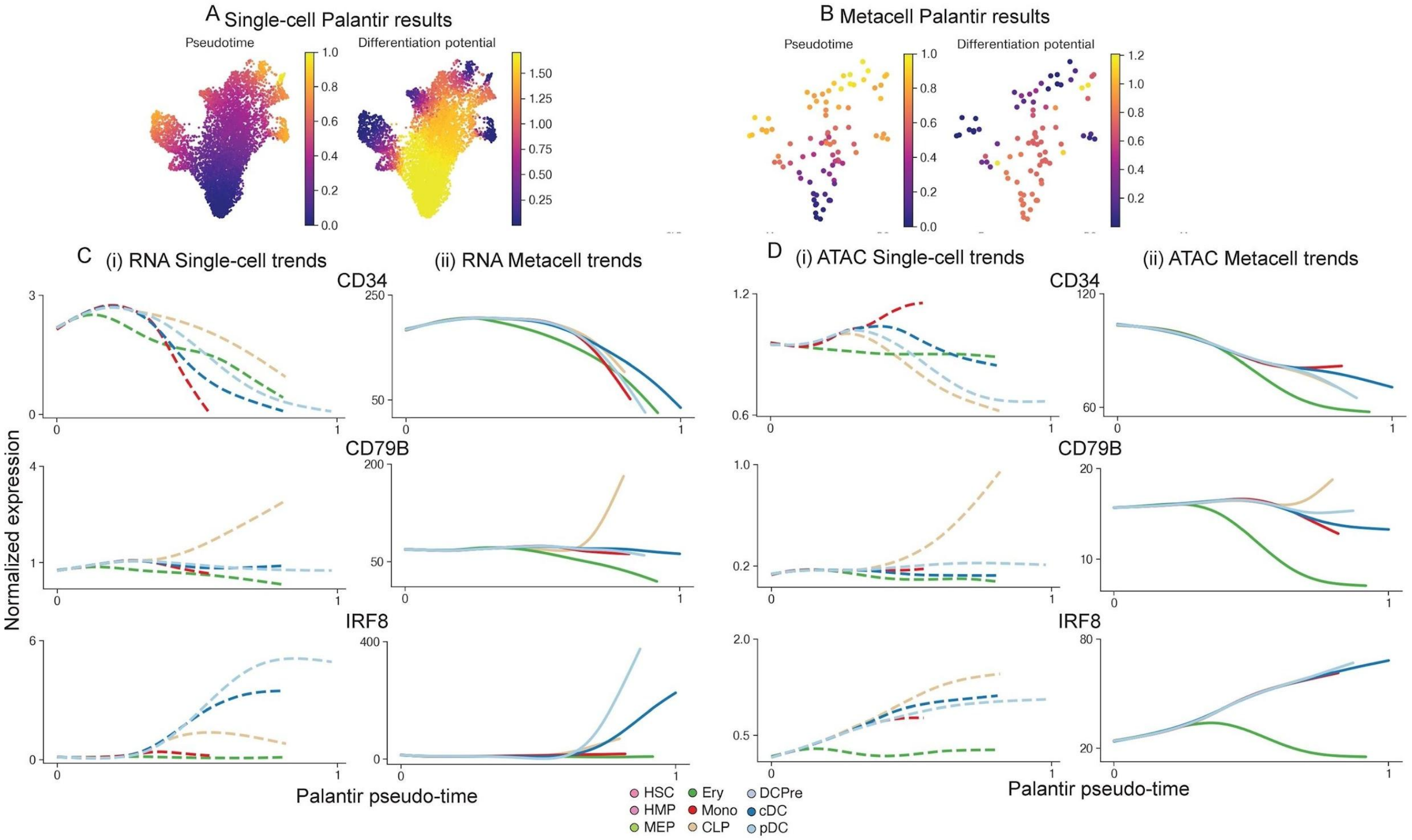

Supplementary Fig. 4: Gene expression and accessibility trends during human hematopoiesis.

A. Single-cell RNA UMAP of a CD34+ bone marrow multiome dataset. Cells colored by pseudotime and differentiation potential values, computed by Palantir on single cells using RNA data.

B. Metacell RNA UMAP of dataset in (A). Cells colored by pseudotime and differentiation potential values, computed by Palantir on RNA data corresponding to ATAC-derived metacells.

C. Gene expression trends at single-cell (left) and metacell (right) resolutions. MAGIC-imputed data was used for plotting single-cell trends.

D. Same as (C), for gene accessibility. CD34 accessibility trends from single-cell data do not monotonically decrease across all lineages due to noise in single-cell ATAC measurements.

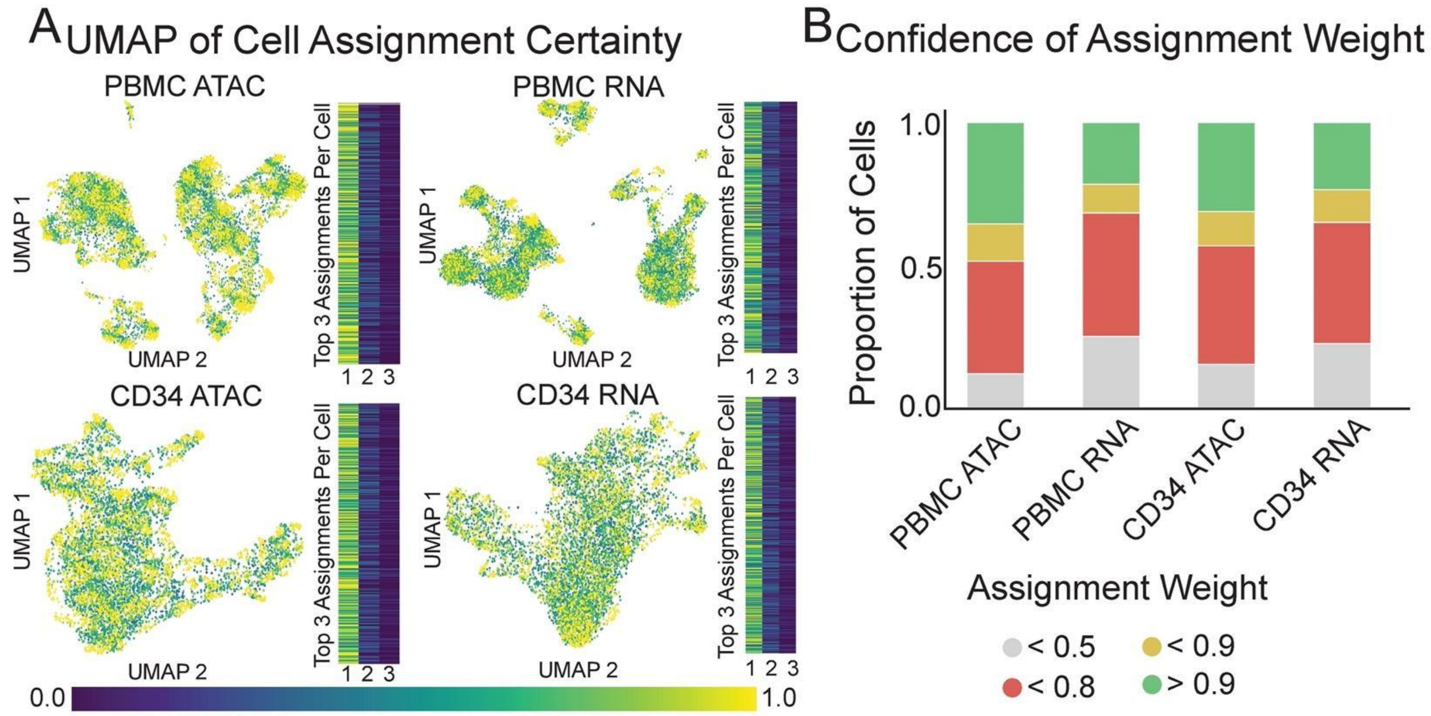

Supplementary Fig. 5: Metacell identification from archetypal analysis

A. UMAPs of the four core benchmarking datasets colored by the maximal assignment weight for each cell. The weights are determined using the matrix A in kernel archetypal analysis (**Fig. 1G**).
B. Stacked bar plots to visualize the proportion of cells with the respective assignment weights.

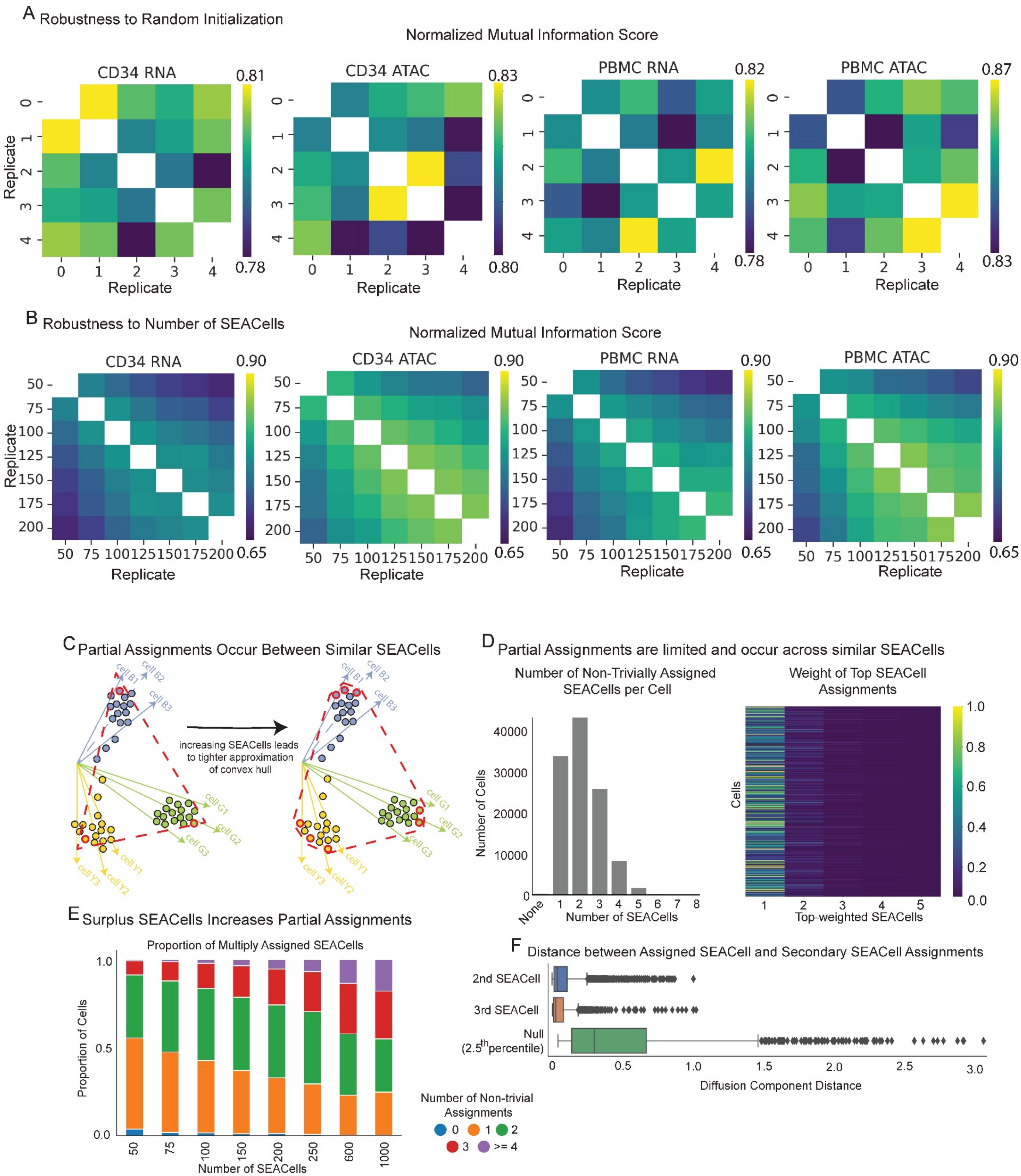
A Robustness to Random Initialization
Normalized Mutual Information Score
CD34 RNA
CD34 ATAC
PBMC RNA
PBMC ATAC
Replicate
B Robustness to Number of SEACells
Normalized Mutual Information Score
C Partial Assignments Occur Between Similar SEACells
increasing SEACells leads to tighter approximation of convex hull
D Partial Assignments are limited and occur across similar SEACells
Number of Non-Trivially Assigned SEACells per Cell
Weight of Top SEACell Assignments
Number of Cells
Number of SEACells
Cells
Top-weighted SEACells
E Surplus SEACells Increases Partial Assignments
Proportion of Multiply Assigned SEACells
Proportion of Cells
Number of SEACells
Number of Non-trivial Assignments
F Distance between Assigned SEACell and Secondary SEACell Assignments
2nd SEACell
3rd SEACell
Null (2.5th percentile)
Diffusion Component Distance

Supplementary Fig. 6: Robustness analysis of SEACells metacells

A. Heatmaps showing the normalized mutual information (NMI) score for different initializations of the SEACells algorithm for the four core benchmarking datasets. NMI is a qualitative measure to compare the consistency of clustering algorithms
B. Heatmaps showing NMI scores for different number of metacells for the core benchmarking datasets.
C. Left: Illustration of kernel archetypal analysis partitioning the cells into three archetypes. The most representative cell for each archetype is highlighted in red. The convex hull spanned by the archetypes is shown as in dotted red lines. Right: Archetypal analysis with greater number of archetypes as the parameter on the same dataset leads to tighter approximation of the convex hull but will also lead to cells being assigned non-trivially to multiple archetypes based on proximity.
D. Left: Histograms showing the distribution of the number of non-trivial SEACell assignments per cell for the CD34+ bone marrow RNA dataset. Right: Heatmap showing the top 5 assignments for each cell. Results are shown for CD34+ bone marrow RNA dataset.
E. Stacked bar plots showing the number of non-trivial assignments per cell with an increasing number of metacells. Results are shown for CD34+ bone marrow RNA dataset.
F. Comparison of diffusion distance between primary and 2nd, 3rd non-trivial SEACell assignments. Null is derived as the 2.5th percentile of distance SEACell distances. All illustrations were generated using the CD34+ bone marrow RNA dataset. Box plots display median, 25^{th}(Q1) and 75th (Q3) percentiles; whiskers extend to the furthest datapoint within the range 1.5 *(Q3-Q1); points beyond that are denoted as outliers. Number of SEACells = 500.

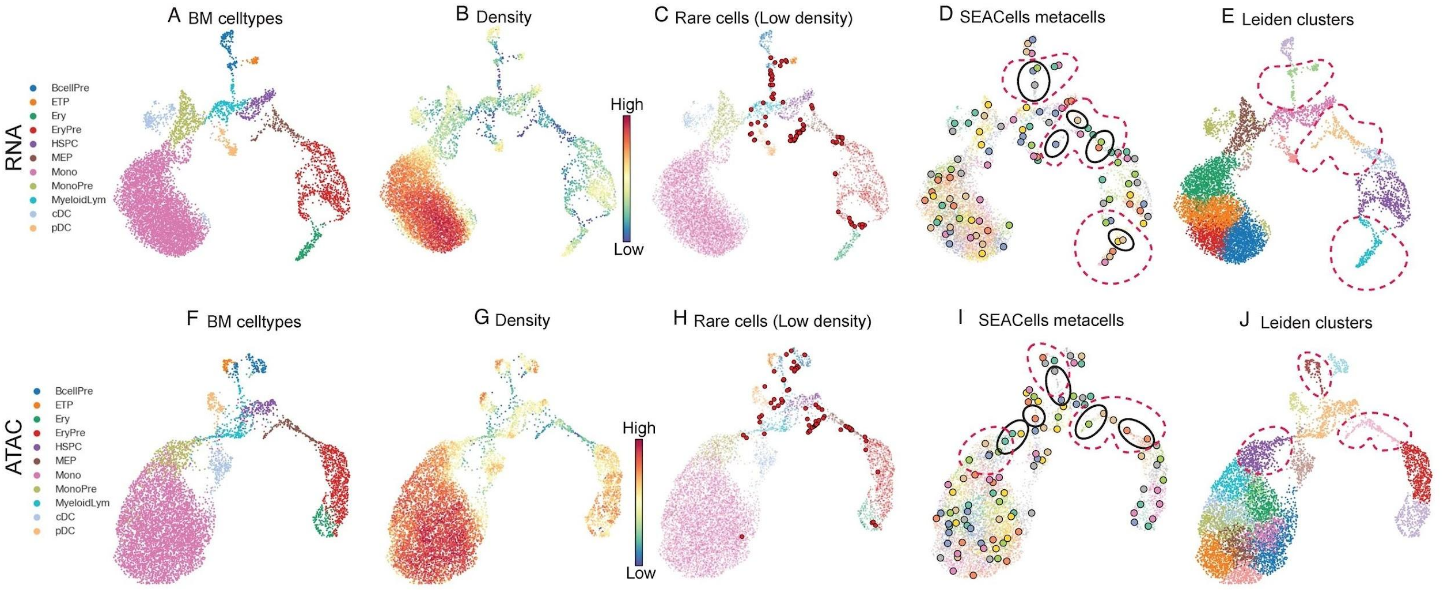

Supplementary Fig. 7: SEACells identify rare intermediate cell states in continuous trajectories

A. Single-cell RNA UMAP of T-cell depleted bone marrow cells from a healthy human donor colored by cell types.

B. UMAP colored by cell density.

C. Cells in low-density regions (bottom percentile of density) are highlighted in red

D. SEACells metacells are highlighted in some low-density regions in black. Dotted red lines represent the Leiden clusters that encompass all the underlying metacells.

E. UMAP colored by Leiden clusters

F–I. Same as (A–E), for ATAC modality of T-cell depleted bone marrow cells from a healthy human donor.

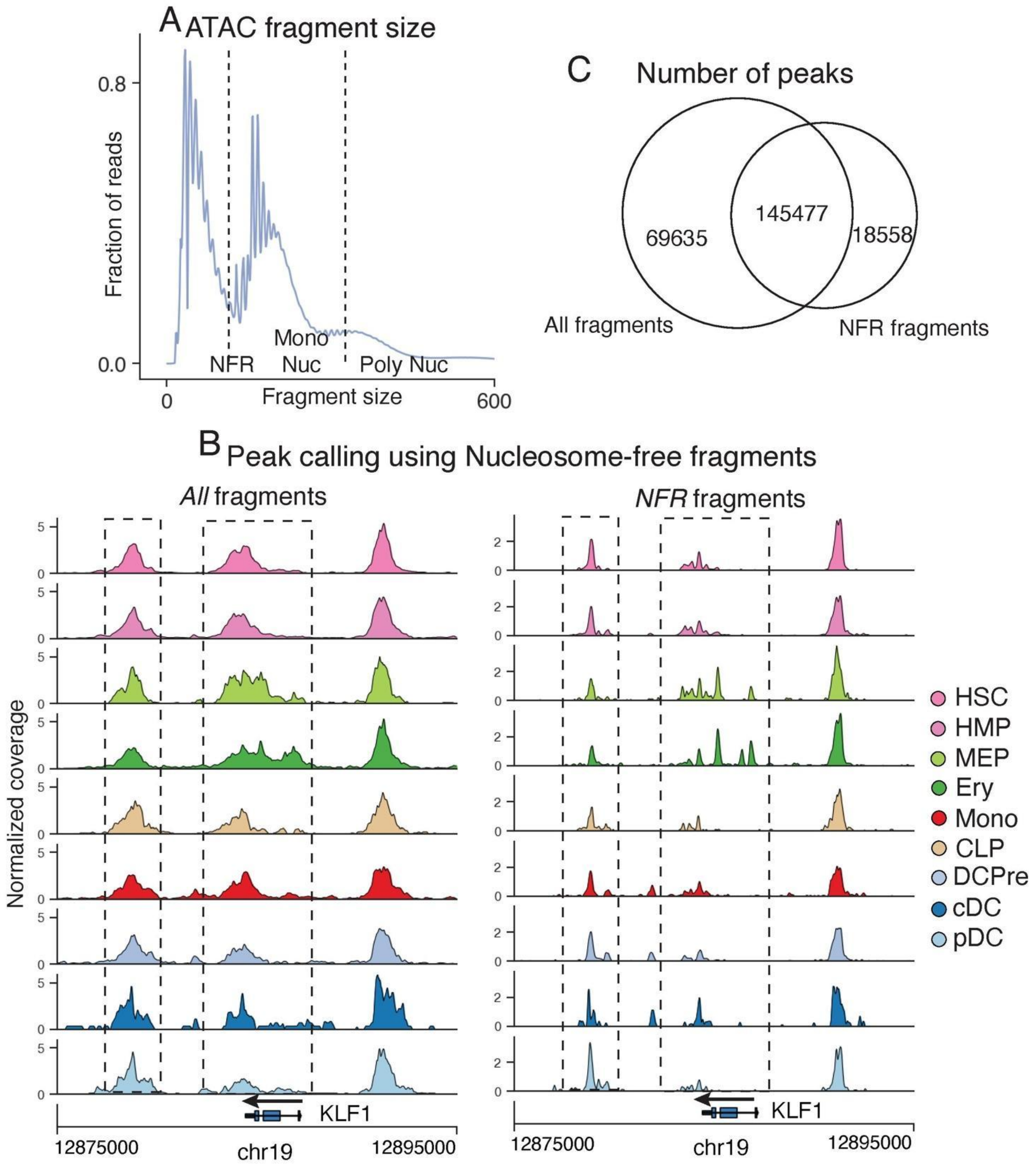

Supplementary Fig. 8: scATAC-seq peak calling

A. ATAC-seq fragments follow a characteristic distribution with well-defined modes. The first mode (<147 bp) represents nucleosome-free (NFR) fragments, and subsequent modes represent mono- and poly-nucleosomes.

B. Accessibility landscape of erythroid factor *KLF1* by cell type, using all ATAC fragments (left) or NFR fragments <147 bp (right) to determine coverage. Using NFR fragments substantially improves the resolution of peaks and inferred regulatory elements (dotted boxes).

C. Venn diagram of number of peaks called using NFR or all ATAC fragments in CD34+ multiome data.

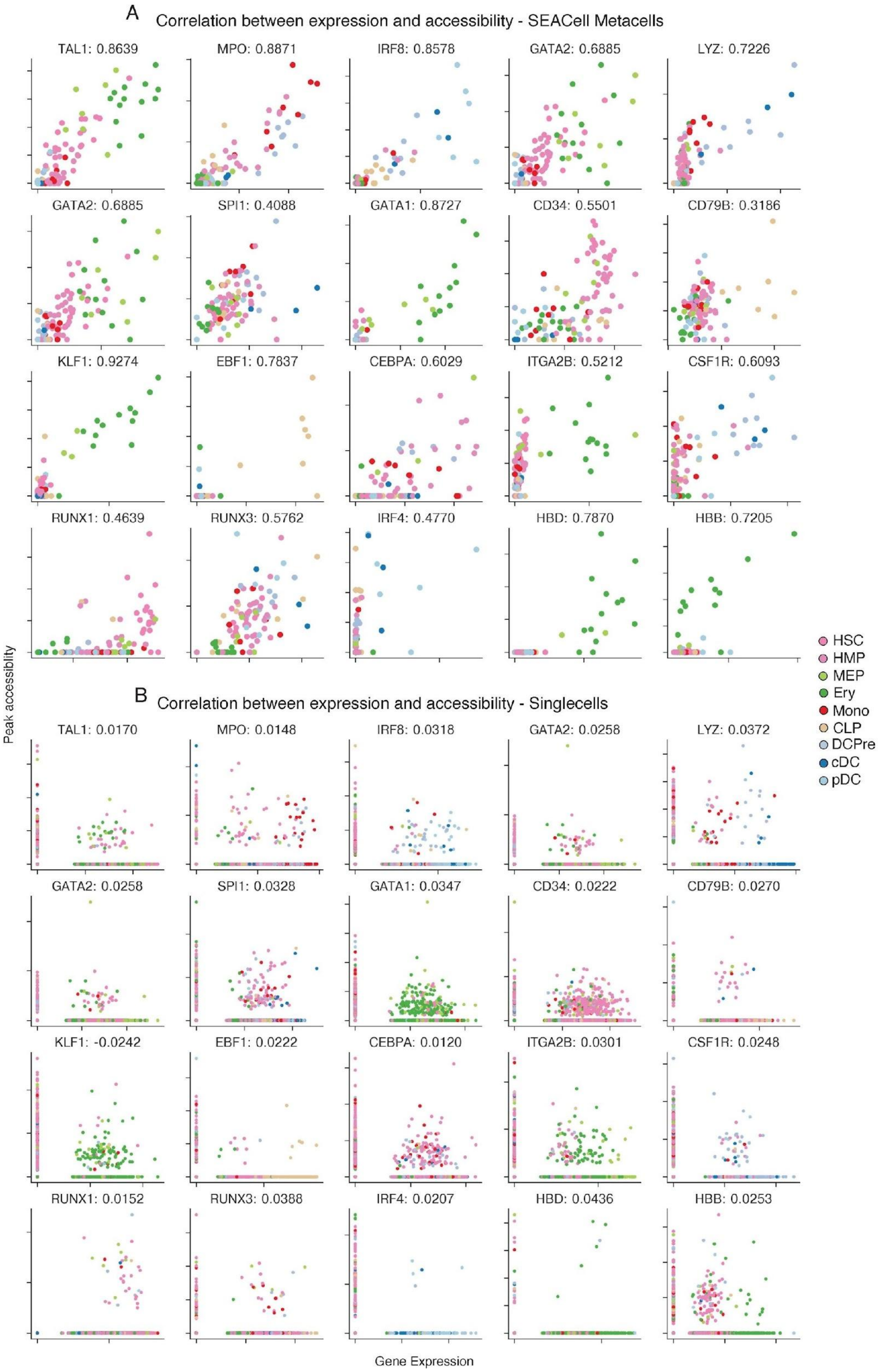
A
Correlation between expression and accessibility - SEACell Metacells
TAL1: 0.8639
MPO: 0.8871
IRF8: 0.8578
GATA2: 0.6885
LYZ: 0.7226
GATA2: 0.6885
SPI1: 0.4088
GATA1: 0.8727
CD34: 0.5501
CD79B: 0.3186
KLF1: 0.9274
EBF1: 0.7837
CEBPA: 0.6029
ITGA2B: 0.5212
CSF1R: 0.6093
RUNX1: 0.4639
RUNX3: 0.5762
IRF4: 0.4770
HBD: 0.7870
HBB: 0.7205
B
Correlation between expression and accessibility - Singlecells
TAL1: 0.0170
MPO: 0.0148
IRF8: 0.0318
GATA2: 0.0258
LYZ: 0.0372
GATA2: 0.0258
SPI1: 0.0328
GATA1: 0.0347
CD34: 0.0222
CD79B: 0.0270
KLF1: -0.0242
EBF1: 0.0222
CEBPA: 0.0120
ITGA2B: 0.0301
CSF1R: 0.0248
RUNX1: 0.0152
RUNX3: 0.0388
IRF4: 0.0207
HBD: 0.0436
HBB: 0.0253
Peak accessibility
Gene Expression
HSC
HMP
MEP
Ery
Mono
CLP
DCPre
cDC
pDC

Supplementary Fig. 9: Comparison of peak-gene correlations using SEACells metacells and single cells

A. Relationship between metacell-aggregated gene expression and accessibility of the most correlated peak for a selection of key hematopoietic genes, computed on the CD34+ multiome data. Meta-cells were derived using the ATAC data modality. Spearman correlations appear next to the gene symbol.
B. Same as in (A), but at the single-cell level.

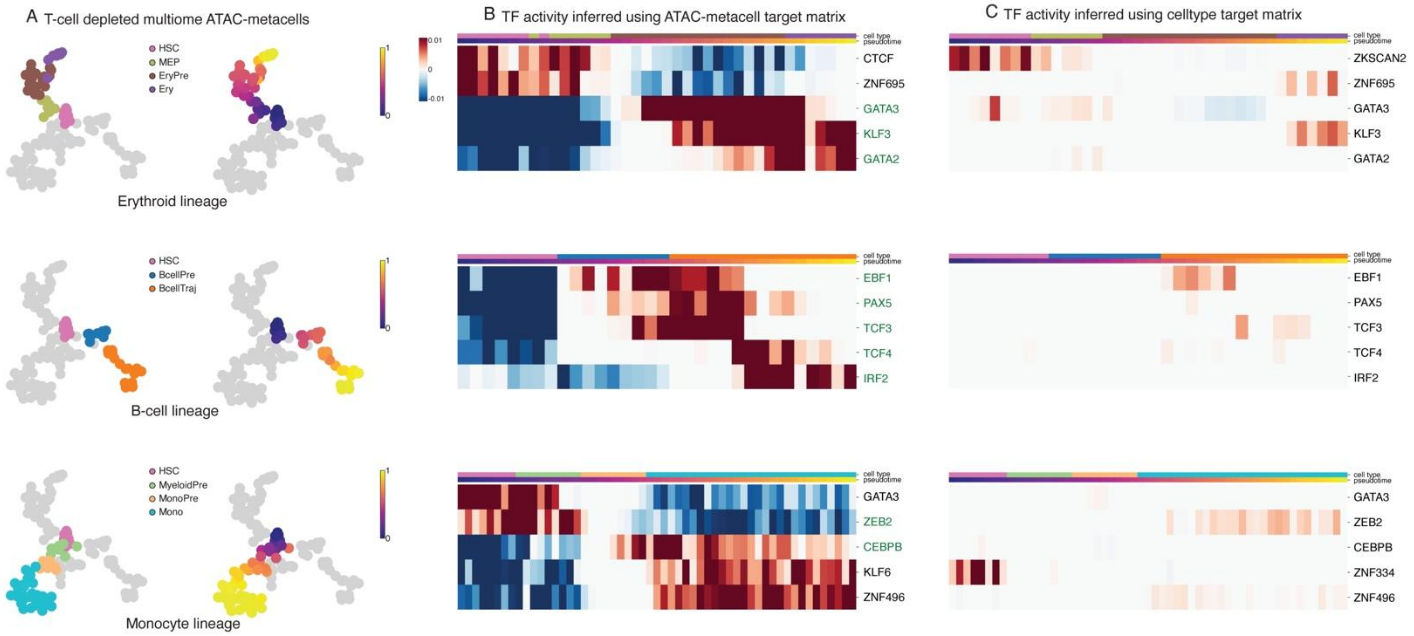

Supplementary Fig. 10: Inference of TF activities across hematopoietic lineages

A. UMAPs highlighting the metacells along erythroid (top), B-cell (middle) and monocyte (bottom) lineages derived using the T-cell depleted bone marrow data. UMAPs on the right are colored by Palantir pseudo-time.
B. Left: Heatmaps showing the top TFs identified for the corresponding lineage using the lasso regression approach (Methods). Known regulators are shown in green. Right: TF activities for the same TFs on the left derived using TF target matrix from cell-type specific peaks.

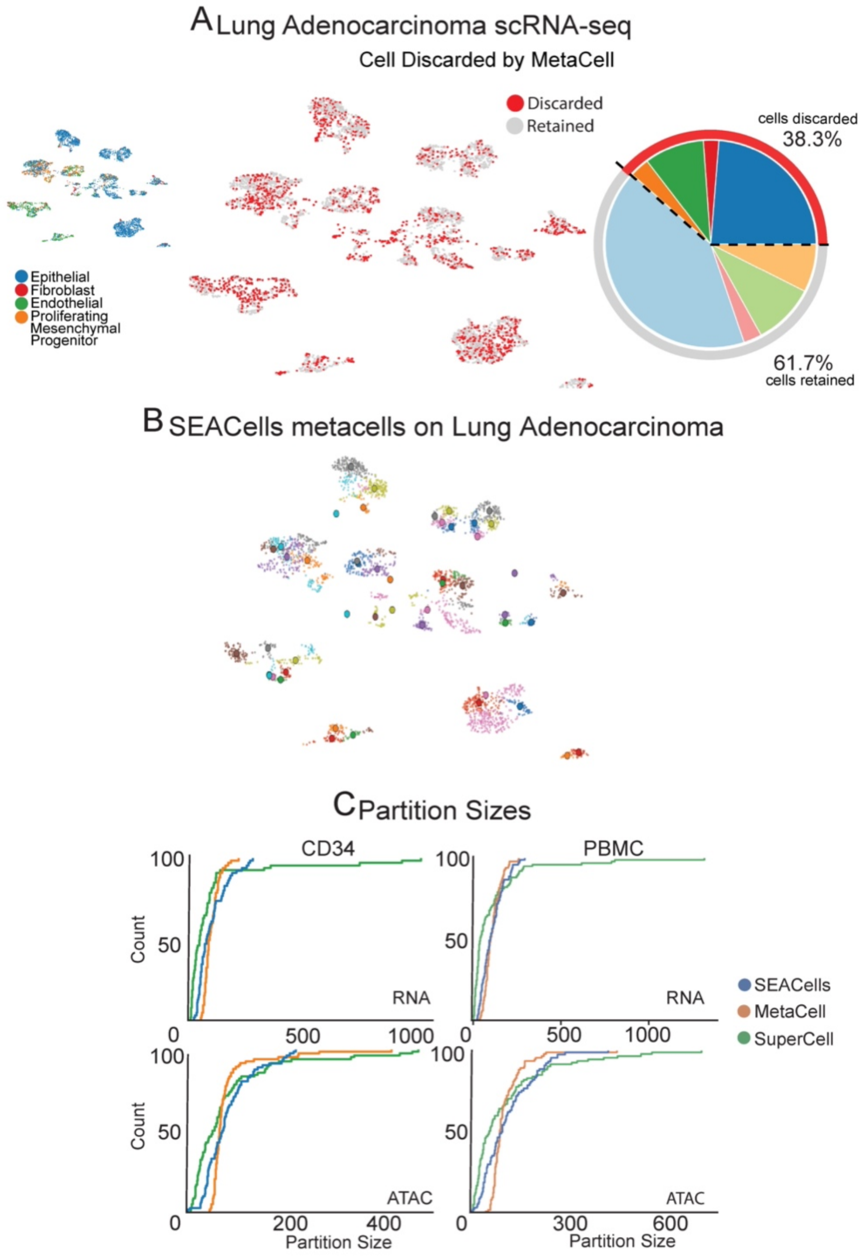

Supplementary Fig. 11: Key characteristics of different metacell approaches

A. UMAP of lung adenocarcinoma scRNA-seq data[12]. A large fraction of cells are pre-filtered by the MetaCell method[2] in this perturbed setting. Cells colored by cell type as derived by [12].

B. SEACells metacells on the same lung adenocarcinoma dataset. No cells were discarded in this analysis.

C. Cumulative distribution plots showing the partition size or number of cells in each metacell. Super-cells produces metacells that are very large and span a large proportion of the high density regions.

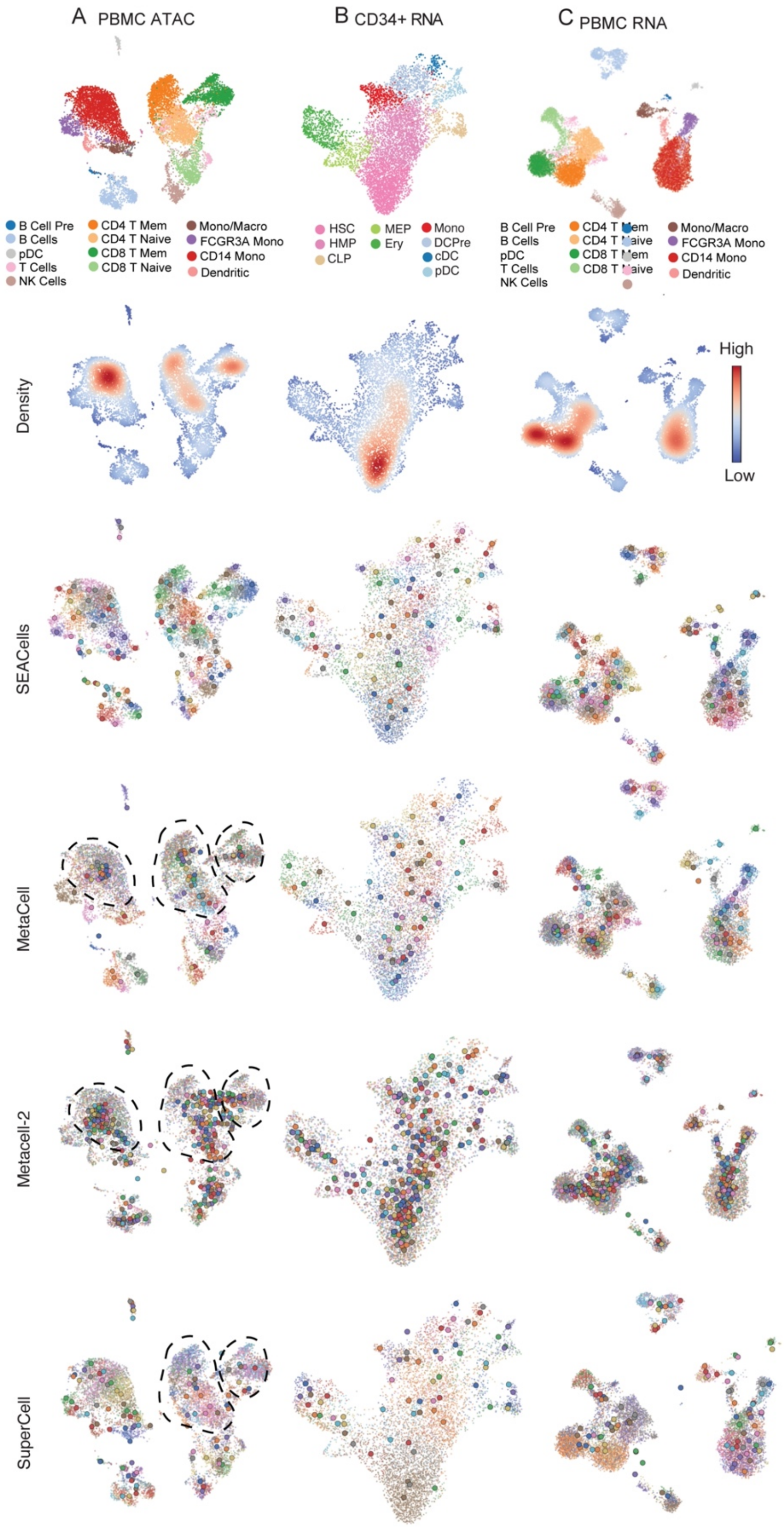
A PBMC ATAC
B CD34+ RNA
C PBMC RNA
B Cell Pre
B Cells
pDC
T Cells
NK Cells
CD4 T Mem
CD4 T Naive
CD8 T Mem
CD8 T Naive
Mono/Macro
FCGR3A Mono
CD14 Mono
Dendritic
HSC
HMP
CLP
MEP
Ery
Mono
DCPre
cDC
pDC
Density
High
Low
SEACells
MetaCell
Metacell-2
SuperCell

Supplementary Fig. 12: Performance of different metacell approaches

A. ATAC modality UMAPs of PBMCs (as in **Fig. 2B**), colored by metacells identified by the specified method or colored by cell density. Dots, cells; circles, metacells. Highlighted regions indicate a lack of well-defined metacells.
B. RNA modality UMAPs of $CD34^+$ bone marrow (as in **Fig. 2D**), colored by metacells or cell density.
C. RNA modality UMAPs of PBMCs (as in **Fig. 2A**), colored by metacells or cell density.

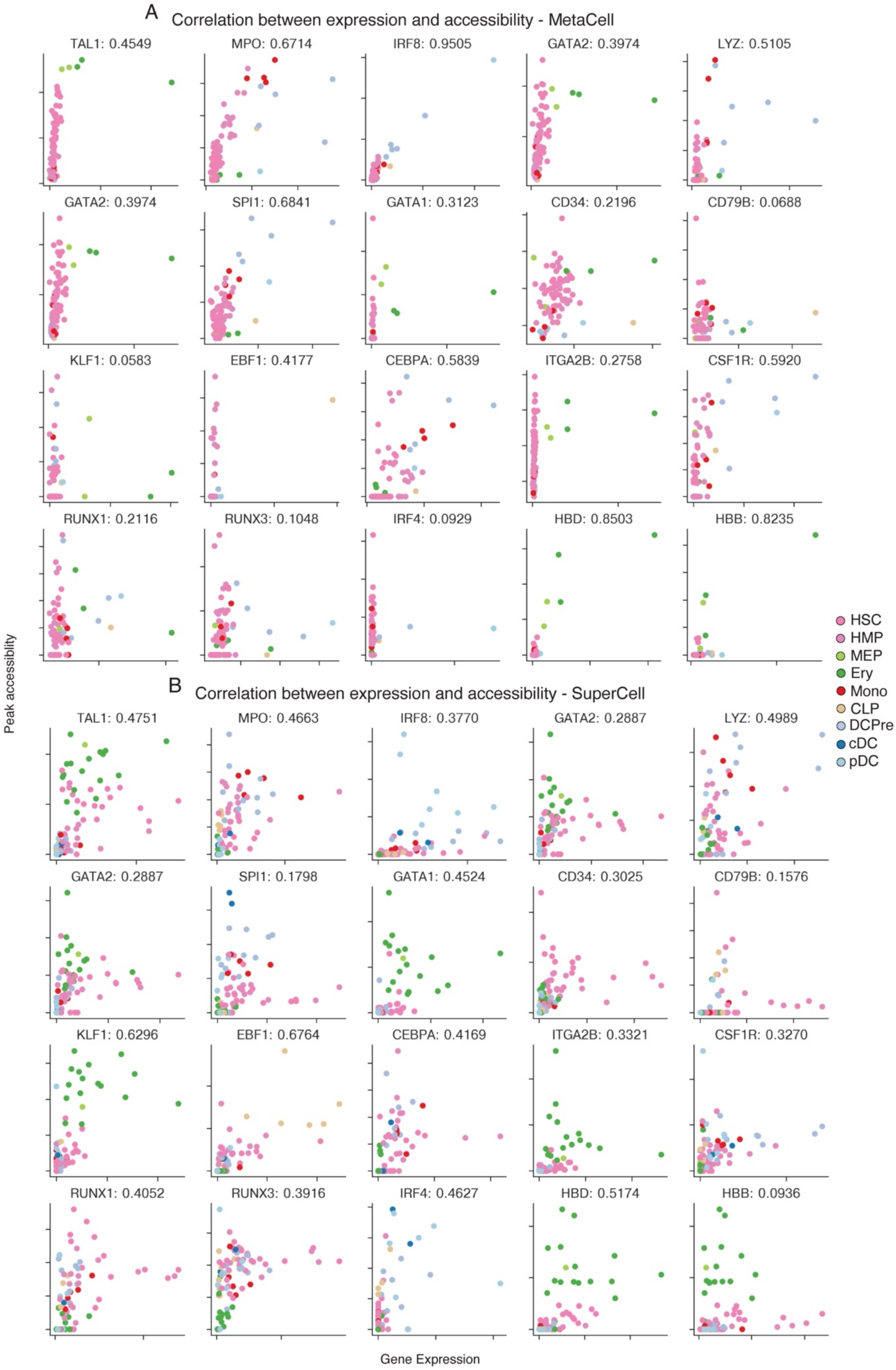

Supplementary Fig. 13: Accessibility peaks and gene expression are not well correlated in MetaCell and SuperCell metacells

A. Relationship between MetaCell[19] aggregated-gene expression and accessibility of the most correlated peak for key hematopoietic genes.
B. Same as (A), computed using SuperCell[18].

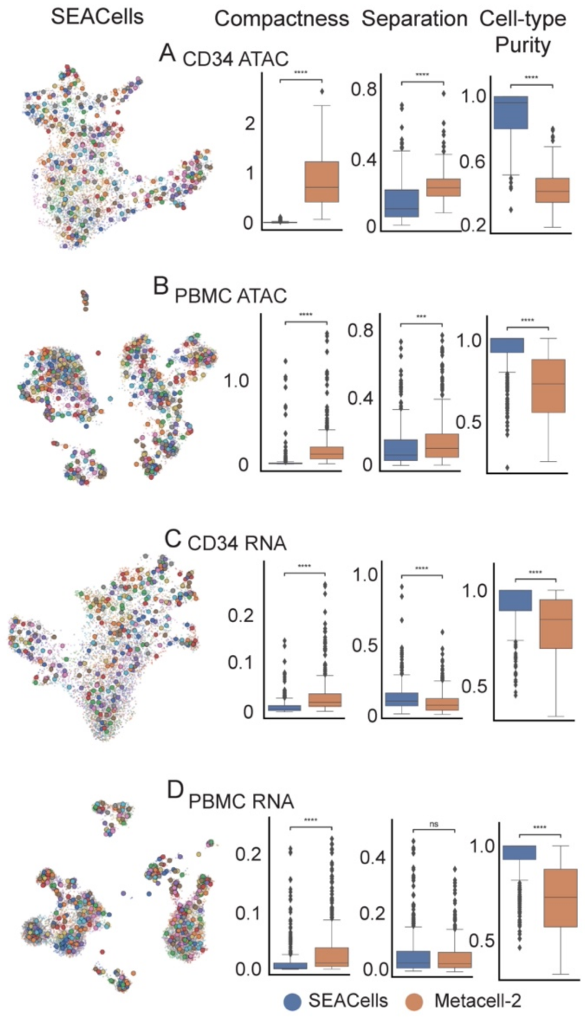

Supplementary Fig. 14: Performance of Metacell-2 in achieving metacell compactness and separation.

A. Left: UMAPs of the CD34+ bone marrow RNA dataset colored by metacells identified SEACells. Metacell-2 automatically determines the number of metacells. SEACells was run using the same number of metacells as Metacell-2 for a fair comparison. Right: Bar plots comparing the compactness, separation and cell-type purity of metacells determined using the two approaches. Two-sided Wilcoxon rank-sum test; ns: P > 0.05, * 0.01 < P < 0.05, ** 0.001 < P < 0.01, *** 0.0001 < P < 0.0001, **** P < 0.0001. Number of metacells = 285.
B. Same as (A), for CD34+ bone marrow ATAC data. Number of metacells = 408.
C. Same as (A), for PBMC RNA data. Number of metacells = 295.
D. Same as (A), for PBMC RNA data. Number of metacells = 424.

Box plots display median, 25th(Q1) and 75th (Q3) percentiles; whiskers extend to the furthest datapoint within the range 1.5 *(Q3-Q1); points beyond that are denoted as outliers.

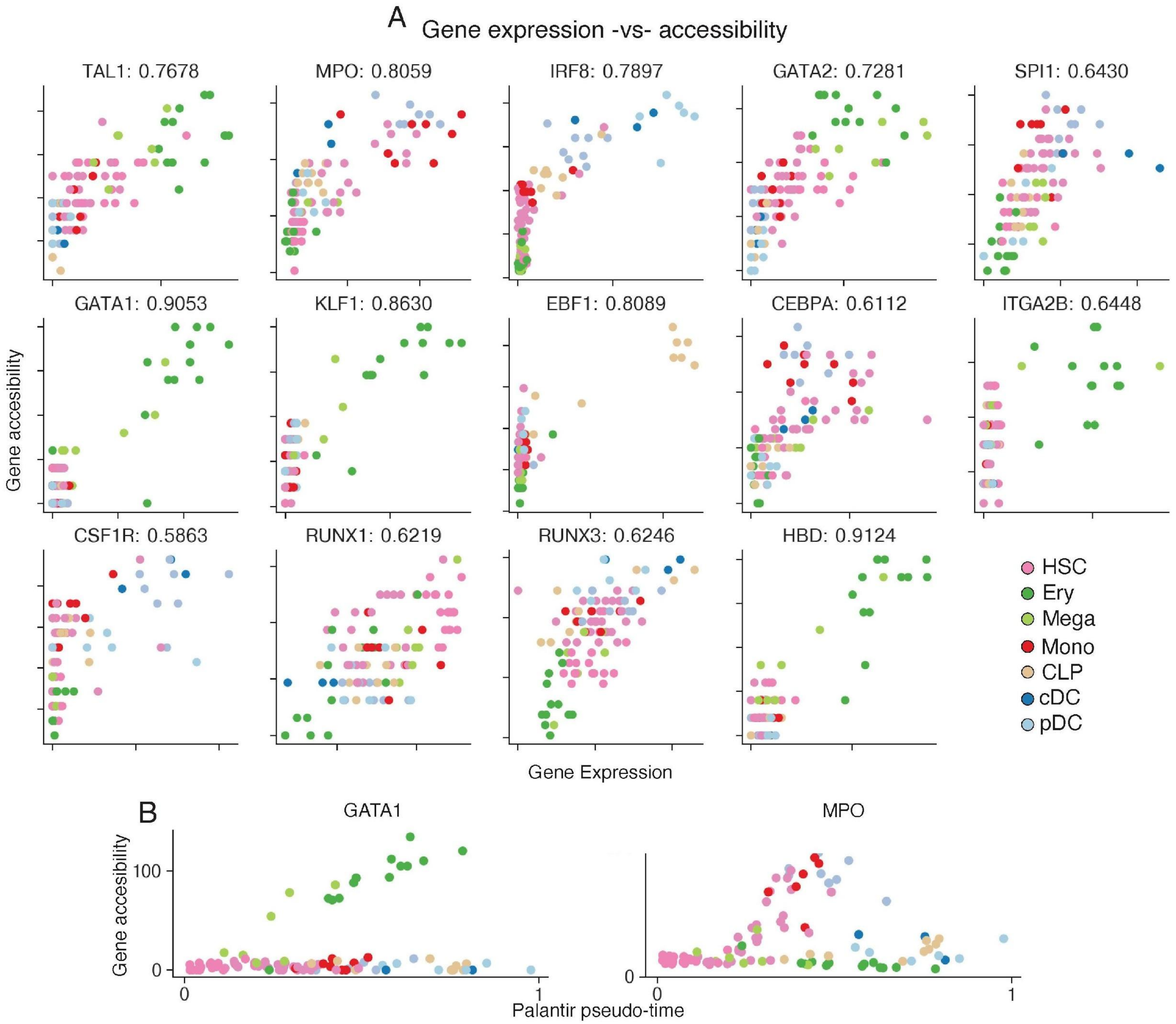

Supplementary Fig. 15: Gene accessibility in CD34⁺ bone marrow

A. Correlation between metacell-aggregated gene expression and gene accessibility scores for a selection of key hematopoietic genes. Spearman correlations computed using the $CD34^+$ bone marrow multiome data are provided next to gene names.

B. Dynamics of gene accessibility scores for *GATA1* (left) and *MPO* (right). Each dot represents a metacell plotted along pseudotime (x-axis).

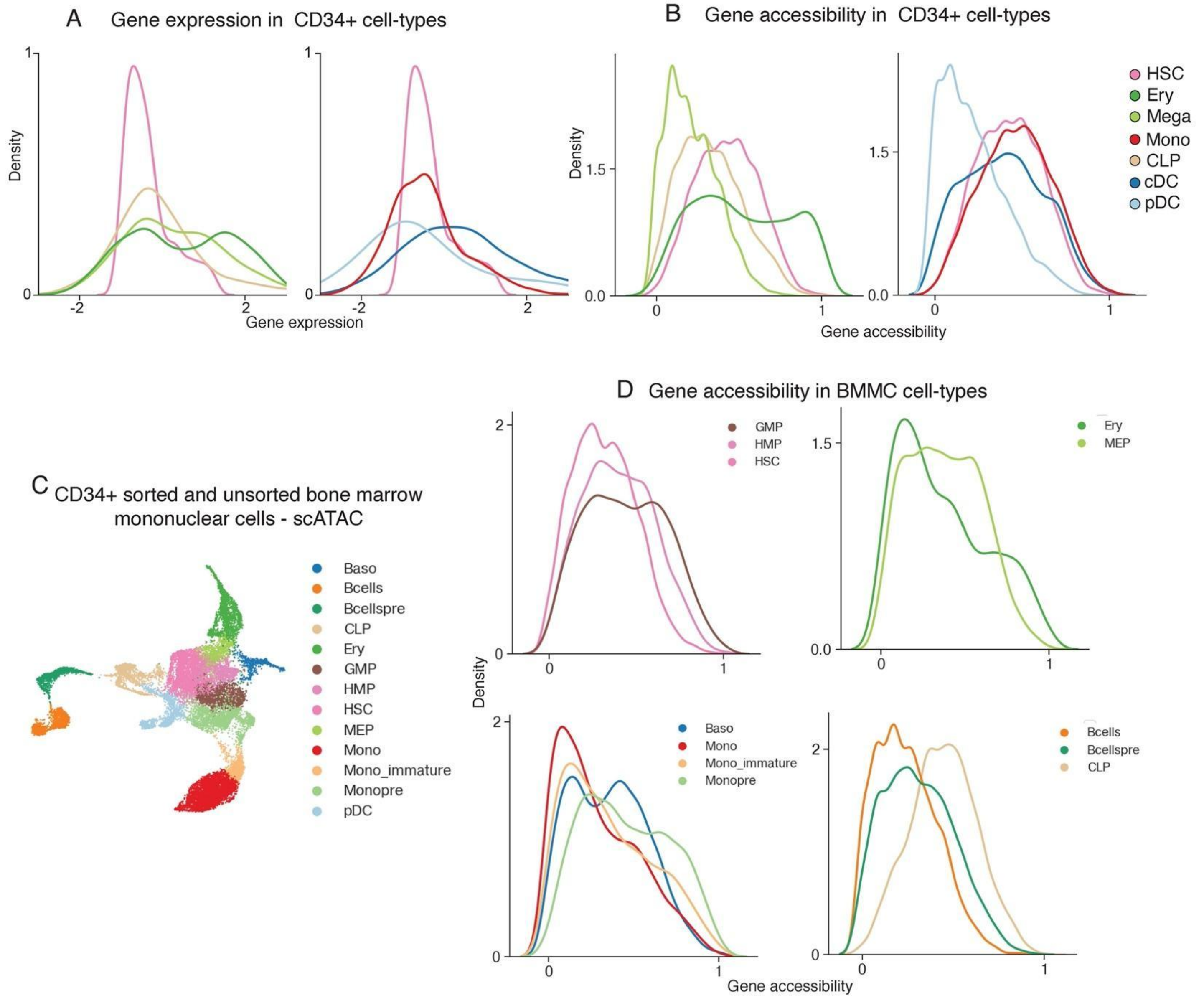

Supplementary Fig. 16: Accessibility dynamics during hematopoietic differentiation

A. Distribution of gene expression for all hematopoietic cell types, using CD34$^+$ bone marrow multiome data.
B. Distribution of gene accessibility for all hematopoietic cell types using CD34$^+$ bone marrow multiome data.
C. UMAP of an scATAC-seq dataset of CD34$^+$-sorted and unsorted bone marrow mononuclear cells (BMMCs).
D. Gene accessibility distributions for highly regulated genes in the BMMC dataset. Peak gene correlations were determined using the CD34$^+$ bone marrow multiome data, since only ATAC modality is available for the BMMC dataset.

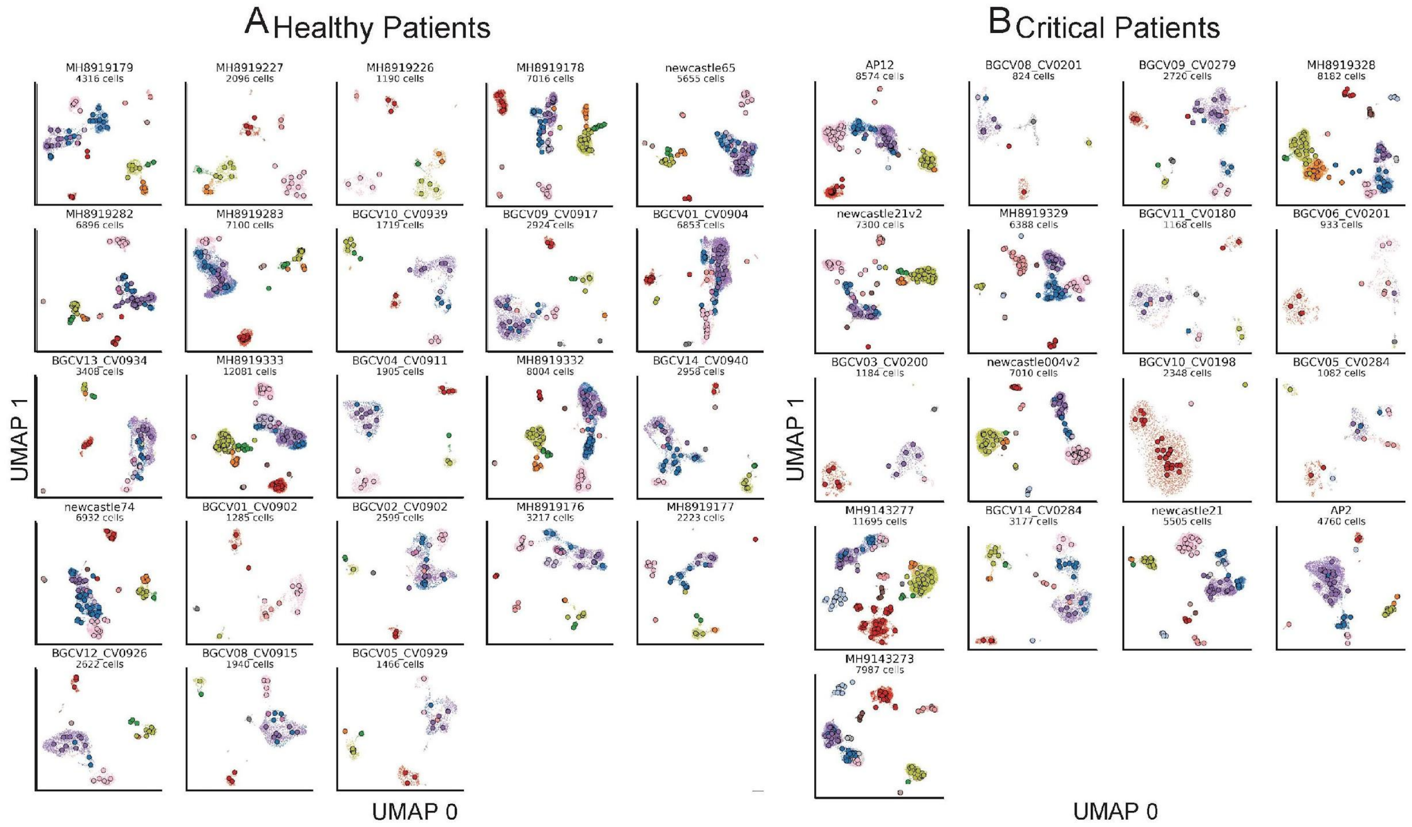

Supplementary Fig. 17: SEACells metacells in a COVID-19 cohort

UMAPs showing SEACells results for healthy donors (A) and COVID-19 patients (B). Each plot represents a single individual.

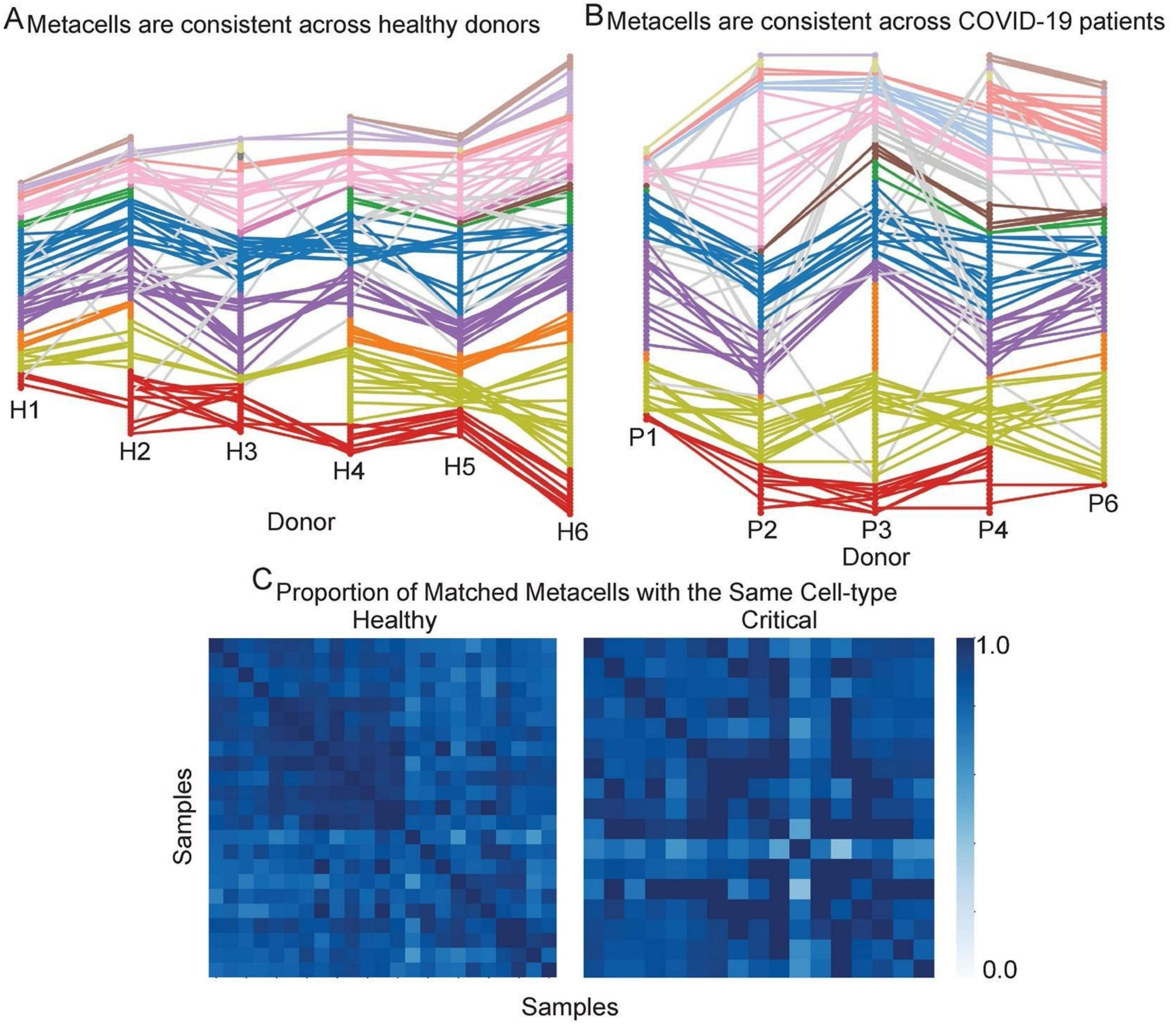

Supplementary Fig. 18: Consistency of SEACells metacells among healthy and COVID-19 patients

A. Metacell states are consistent and reproducible between pairs of healthy individuals. Mutually neighboring metacells between different pairs of healthy donors are connected by edges.

B. Same as (A), for COVID-19 patients.

C. Proportion of mutually neighboring edges that match to the same cell-type for healthy donors (left) and COVID-19 patients (right). On average, 88% of edges between healthy donors and 87% of edges between COVID-19 patients are consistent.

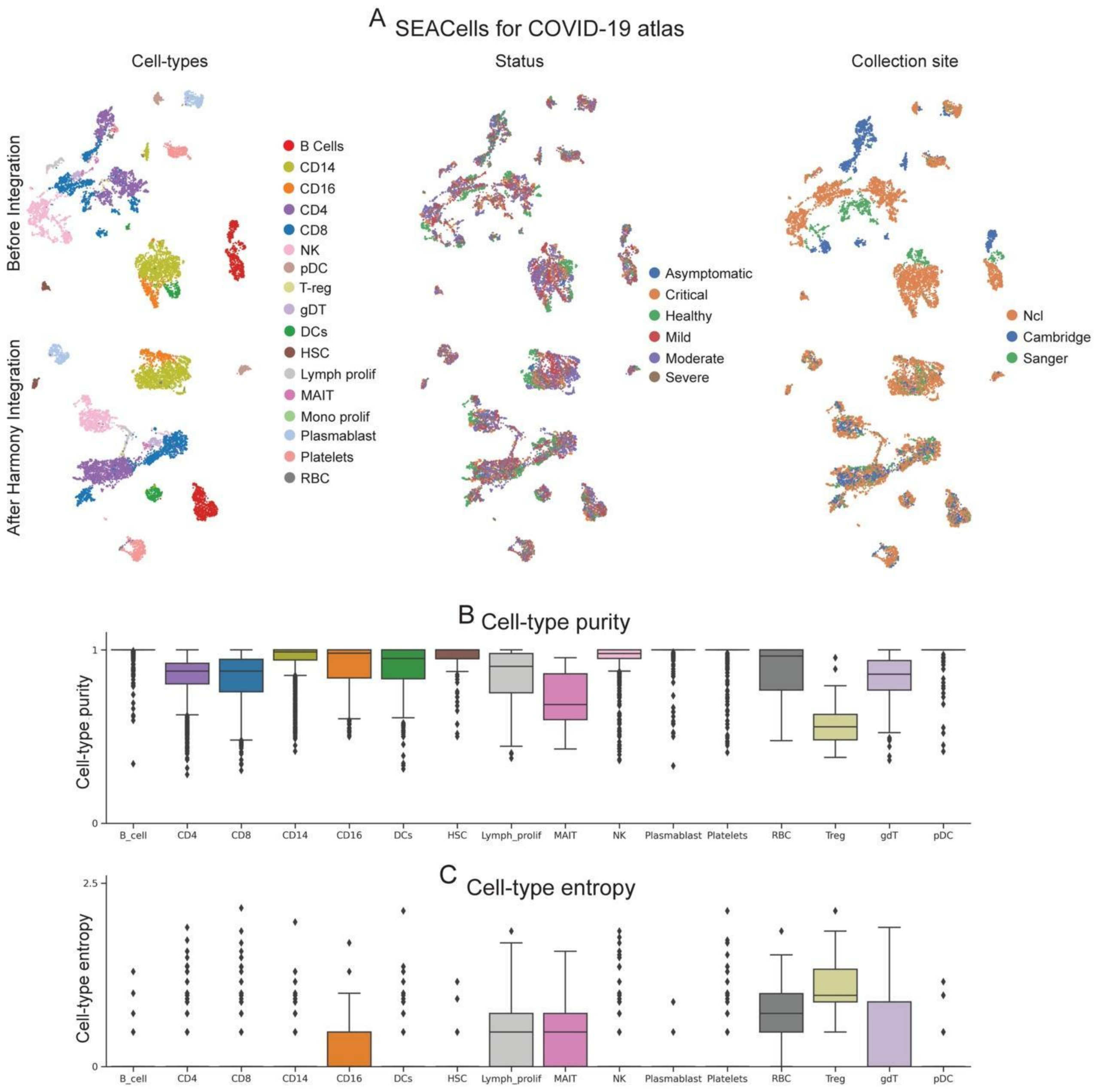

Supplementary Fig. 19: Scalability of SEACells to atlas-scale single-cell datasets

A. UMAPs showing metacells from ~120 samples spanning >600k cells demonstrating the scalability of SEACells to atlas-scale data. Top: UMAPs before batch correction. Bottom: UMAPs after batch corrections using Harmony[26].
B. Bar plots showing the cell-type purity of metacells in (A). Number of metacells = 8092.
C. Bar plots comparing the entropy of cell-types in metacell neighborhoods following batch correction.

Box plots display median, 25th(Q1) and 75th (Q3) percentiles; whiskers extend to the furthest datapoint within the range 1.5 *(Q3-Q1); points beyond that are denoted as outliers.

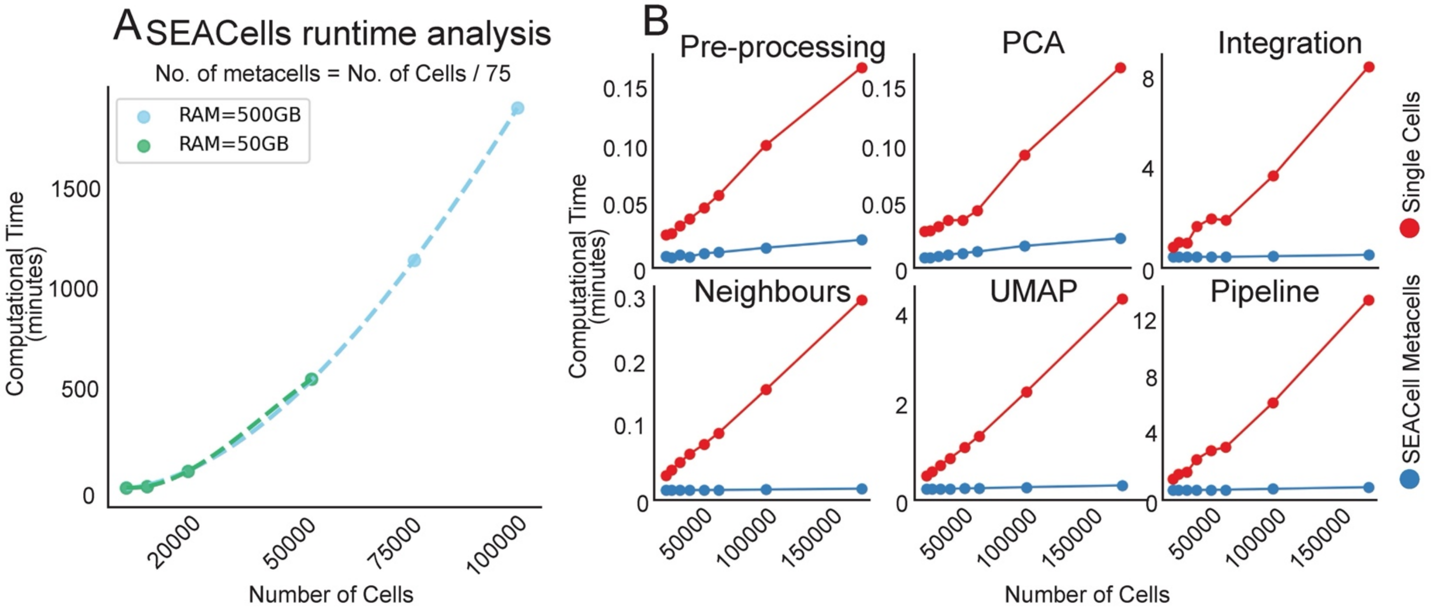

Supplementary Fig. 20: SEACells runtime analysis

A. Time to compute SEACells metacells, as a function of single-cell dataset size. The number of metacells was fixed as number of cells / 75.

B. Runtime comparison of SEACells metacells and single cells for different single-cell tasks. Preprocessing involves normalization and batch correction. Pipeline refers to runtime for all tasks shown.

References

1 Hart, Y. *et al.* Inferring biological tasks using Pareto analysis of high-dimensional data. *Nat Methods* **12**, 233-235, 233 p following 235 (2015). https://doi.org:10.1038/nmeth.3254

2 Wei, S. C. *et al.* Negative Co-stimulation Constrains T Cell Differentiation by Imposing Boundaries on Possible Cell States. *Immunity* **50**, 1084-1098 e1010 (2019). https://doi.org:10.1016/j.immuni.2019.03.004

3 Mohammadi, S., Davila-Velderrain, J. & Kellis, M. A multiresolution framework to characterize single-cell state landscapes. *Nat Commun* **11**, 5399 (2020). https://doi.org:10.1038/s41467-020-18416-6

4 Genomics, X. *CellRanger ARC*, <https://support.10xgenomics.com/single-cell-multiome-atac-gex/software/pipelines/latest/what-is-cell-ranger-arc?src=social&lss=linkedin&cnm=soc-li-ra_g-program-li-ra_g-program&cid=7011P000000y072> (2021).

5 Wolock, S. L., Lopez, R. & Klein, A. M. Scrublet: Computational Identification of Cell Doublets in Single-Cell Transcriptomic Data. *Cell Syst* **8**, 281-291 e289 (2019). https://doi.org:10.1016/j.cels.2018.11.005

6 Setty, M. *et al.* Characterization of cell fate probabilities in single-cell data with Palantir. *Nat Biotechnol* **37**, 451-460 (2019). https://doi.org:10.1038/s41587-019-0068-4

7 Levine, J. H. *et al.* Data-Driven Phenotypic Dissection of AML Reveals Progenitor-like Cells that Correlate with Prognosis. *Cell* **162**, 184-197 (2015). https://doi.org:10.1016/j.cell.2015.05.047

8 Wolf, F. A., Angerer, P. & Theis, F. J. SCANPY: large-scale single-cell gene expression data analysis. *Genome Biol* **19**, 15 (2018). https://doi.org:10.1186/s13059-017-1382-0

9 van Dijk, D. *et al.* Recovering Gene Interactions from Single-Cell Data Using Data Diffusion. *Cell* **174**, 716-729 e727 (2018). https://doi.org:10.1016/j.cell.2018.05.061

10 Granja, J. M. *et al.* ArchR is a scalable software package for integrative single-cell chromatin accessibility analysis. *Nat Genet* **53**, 403-411 (2021). https://doi.org:10.1038/s41588-021-00790-6

11 Stuart, T. *et al.* Comprehensive Integration of Single-Cell Data. *Cell* **177**, 1888-1902 e1821 (2019). https://doi.org:10.1016/j.cell.2019.05.031

12 Laughney, A. M. *et al.* Regenerative lineages and immune-mediated pruning in lung cancer metastasis. *Nat Med* **26**, 259-269 (2020). https://doi.org:10.1038/s41591-019-0750-6

13 Granja, J. M. *et al.* Single-cell multiomic analysis identifies regulatory programs in mixed-phenotype acute leukemia. *Nat Biotechnol* **37**, 1458-1465 (2019). https://doi.org:10.1038/s41587-019-0332-7

14 Pijuan-Sala, B. *et al.* A single-cell molecular map of mouse gastrulation and early organogenesis. *Nature* **566**, 490-495 (2019). https://doi.org:10.1038/s41586-019-0933-9

15 Genomics, X. *PBMC CITE-seq from a Healthy Donor*, <https://www.10xgenomics.com/resources/datasets/pbmc-from-a-healthy-donor-granulocytes-removed-through-cell-sorting-10-k-1-standard-2-0-0> (

16 Shasha, C., Tian, Y., Mair, F., Miller, H. & Gottardo, R. Superscan: Supervised Single-Cell Annotation. *bioRxiv* (2021). https://doi.org:https://doi.org/10.1101/2021.05.20.445014

17 Coifman, R. R. *et al.* Geometric diffusions as a tool for harmonic analysis and structure definition of data: diffusion maps. *Proc Natl Acad Sci U S A* **102**, 7426-7431 (2005). https://doi.org:10.1073/pnas.0500334102

18 Bilous, M. *et al.* Metacells untangle large and complex single-cell transcriptome networks. *BMC Bioinformatics* **23** (2022). https://doi.org:https://doi.org/10.1186/s12859-022-04861-1

19 Baran, Y. *et al.* MetaCell: analysis of single-cell RNA-seq data using K-nn graph partitions. *Genome Biol* **20**, 206 (2019). https://doi.org:10.1186/s13059-019-1812-2

20 Ben-Kiki, O., Bercovich, A., Lifshitz, A. & Tanay, A. Metacell-2: a divide-and-conquer metacell algorithm for scalable scRNA-seq analysis. *Genome Biol* **23**, 100 (2022). https://doi.org:10.1186/s13059-022-02667-1

21 Lopez, R., Regier, J., Cole, M. B., Jordan, M. I. & Yosef, N. Deep generative modeling for single-cell transcriptomics. *Nat Methods* **15**, 1053-1058 (2018). https://doi.org:10.1038/s41592-018-0229-2

22 Ashuach, T., Reidenbach, D. A., Gayoso, A. & Yosef, N. PeakVI: A deep generative model for single-cell chromatin accessibility analysis. *Cell Rep Methods* **2**, 100182 (2022). https://doi.org:10.1016/j.crmeth.2022.100182

23 Hastie, T. & Tibshirani, R. Generalized Additive-Models - Some Applications. *J Am Stat Assoc* **82**, 371-386 (1987). https://doi.org:Doi 10.2307/2289439

24 Grant, C. E., Bailey, T. L. & Noble, W. S. FIMO: scanning for occurrences of a given motif. *Bioinformatics* **27**, 1017-1018 (2011). https://doi.org:10.1093/bioinformatics/btr064

25 Weirauch, M. T. *et al.* Determination and inference of eukaryotic transcription factor sequence specificity. *Cell* **158**, 1431-1443 (2014). https://doi.org:10.1016/j.cell.2014.08.009
26 Korsunsky, I. *et al.* Fast, sensitive and accurate integration of single-cell data with Harmony. *Nat Methods* **16**, 1289-1296 (2019). https://doi.org:10.1038/s41592-019-0619-0
