# Supplementary material for: SEACells infers transcriptional and epigenomic cellular states from single-cell genomics data
Source: Nat Biotechnol. 2023 Mar 27;41(12):1746–57. doi: 10.1038/s41587-023-01716-9 (PMC10713451; doi:10.1038/s41587-023-01716-9)
Supplement: Supplementary file 2 — Reporting Summary [file 41587_2023_1716_MOESM2_ESM.pdf]

nature research

Corresponding author(s): Dana Pe'er
Manu Setty

Last updated by author(s): 2/5/2023

Reporting Summary

Nature Research wishes to improve the reproducibility of the work that we publish. This form provides structure for consistency and transparency in reporting. For further information on Nature Research policies, see our Editorial Policies and the Editorial Policy Checklist.

Statistics

For all statistical analyses, confirm that the following items are present in the figure legend, table legend, main text, or Methods section.

n/a	Confirmed	
☐	☒	The exact sample size (*n*) for each experimental group/condition, given as a discrete number and unit of measurement
☐	☒	A statement on whether measurements were taken from distinct samples or whether the same sample was measured repeatedly
☐	☒	The statistical test(s) used AND whether they are one- or two-sided<br>*Only common tests should be described solely by name; describe more complex techniques in the Methods section.*
☐	☒	A description of all covariates tested
☐	☒	A description of any assumptions or corrections, such as tests of normality and adjustment for multiple comparisons
☐	☒	A full description of the statistical parameters including central tendency (e.g. means) or other basic estimates (e.g. regression coefficient) AND variation (e.g. standard deviation) or associated estimates of uncertainty (e.g. confidence intervals)
☐	☒	For null hypothesis testing, the test statistic (e.g. F, t, r) with confidence intervals, effect sizes, degrees of freedom and P value noted<br>*Give P values as exact values whenever suitable.*
☒	☐	For Bayesian analysis, information on the choice of priors and Markov chain Monte Carlo settings
☒	☐	For hierarchical and complex designs, identification of the appropriate level for tests and full reporting of outcomes
☐	☒	Estimates of effect sizes (e.g. Cohen's *d*, Pearson's *r*), indicating how they were calculated

Our web collection on statistics for biologists contains articles on many of the points above.

Software and code

Policy information about availability of computer code

Data collection	10X Cellranger arc v2.0.0 was used for count matrix and fragment file generation for the Multiome data
Data analysis	The following tools and version were used for analysis<br>1. SEACells: Algorithm developed in the manuscript. https://github.com/dpeerlab/SEACells. Version v0.2.0<br>2. scanpy for analysis of single-cell RNA-seq data. scanpy v1.7.2<br>3. Palantir for trajectory analysis palantir. v1.0.0<br>4. ArchR for ATAC analysis: v0.9.5<br>5. R version 3.6.3<br>6. FIMO for peak calling: v5.1.1<br>7. chromVAR: v1.16.0<br>8. phenograph: v1.5.7

For manuscripts utilizing custom algorithms or software that are central to the research but not yet described in published literature, software must be made available to editors and reviewers. We strongly encourage code deposition in a community repository (e.g. GitHub). See the Nature Research guidelines for submitting code & software for further information.

Data

Policy information about availability of data

All manuscripts must include a data availability statement. This statement should provide the following information, where applicable:

- Accession codes, unique identifiers, or web links for publicly available datasets
- A list of figures that have associated raw data
- A description of any restrictions on data availability

The newly generated CD34+ bone marrow and T-cell depleted bone marrow multiome datasets are available on GEO (https://www.ncbi.nlm.nih.gov/geo/query/acc.cgi?acc=GSE200046). Filtered and processed count matrices including cell-type annotations and ATAC fragment files are available on Zenodo at 10.5281/zenodo.638326974. The following publicly available datasets were used in the manuscript: 10X PBMC Multiome18, 10X PBMC CITE-seq75, scRNA-seq of lung adenocarcinoma (https://www.ncbi.nlm.nih.gov/geo/query/acc.cgi?acc=GSE123904)37, mouse gastrulation atlas (https://www.ebi.ac.uk/biostudies/arrayexpress/studies/E-MTAB-6967) 20.

Field-specific reporting

Please select the one below that is the best fit for your research. If you are not sure, read the appropriate sections before making your selection.

- [x] Life sciences
- [ ] Behavioural & social sciences
- [ ] Ecological, evolutionary & environmental sciences

For a reference copy of the document with all sections, see nature.com/documents/nr-reporting-summary-flat.pdf

Life sciences study design

All studies must disclose on these points even when the disclosure is negative.

Sample size	Two technical replicates each of CD34+ sorted bone marrow and T-cell depleted bone marrow were used for multiomic profiling. No sample size calculation was performed. The number of replicates were chosen to ensure sufficient number of cells for the analysis.
Data exclusions	Mature B cells and other cell types were excluded from analysis of both the bone marrow datasets to enrich for the hematopoietic trajectories. NK cells were excluded from analysis of T-cell depleted bone marrow sample since NK cells do not differentiate in the bone marrow.
Replication	The robustness of SEACells was verified for different initializations and different number of number of metacells. Each analysis was run independently.
Randomization	Not Applicable since the study did not involve human trials
Blinding	Not Applicable since the cells were purchased from blinded donors.

Reporting for specific materials, systems and methods

We require information from authors about some types of materials, experimental systems and methods used in many studies. Here, indicate whether each material, system or method listed is relevant to your study. If you are not sure if a list item applies to your research, read the appropriate section before selecting a response.

Materials & experimental systems

n/a	Involved in the study	
☐	☒	Antibodies
☒	☐	Eukaryotic cell lines
☒	☐	Palaeontology and archaeology
☒	☐	Animals and other organisms
☒	☐	Human research participants
☒	☐	Clinical data
☒	☐	Dual use research of concern

Methods

n/a	Involved in the study	
☒	☐	ChIP-seq
☒	☐	Flow cytometry
☒	☐	MRI-based neuroimaging

Antibodies

Antibodies used	CD3 Monoclonal Antibody (UCHT1), PE-Cyanine7, eBioscience™ (25-0038-42)
Validation	This Antibody was verified by Relative expression to ensure that the antibody binds to the antigen stated.
